# How do emotions respond to outcome values and influence choice?

**DOI:** 10.1007/s00426-024-02001-3

**Published:** 2024-07-10

**Authors:** Aikaterini Grimani, Ayse Yemiscigil, Qing Wang, Georgi Kirilov, Laura Kudrna, Ivo Vlaev

**Affiliations:** 1 Warwick Business School, University of Warwick, Scarman Rd, Coventry, CV4 7AL UK; 2 Human Flourishing Program, Harvard University, Boston, US; 3 Sofia University, Sofia, Bulgaria; 4 University of Birmingham, Birmingham, UK

**Keywords:** Anticipatory emotions, Anticipated emotions, Risk, Decision making, Outcome values’ effect

## Abstract

Emotions powerfully, predictably, and pervasively influence decision making. The risk-as-feelings hypothesis states that two kinds of emotions are important in decision-making, anticipatory emotions and anticipated emotions. We empirically investigated whether and how anticipatory and anticipated emotions may change as a function of outcome values and whether anticipatory or anticipated emotions may explain the influence of outcome values on risky choice. To study the effects of value on emotions and choice, we offered people hypothetical large amounts ($100, $200, $300, $400) and incentivized moderate amounts ($10, $20, $30, $40) as prospects in gambles over two consecutive studies. Using a representative sample from the US to ensure the generalizability of the findings, each participant in our two studies made choices in gain and loss domains. Overall, anticipatory and anticipated emotions responded very similarly to changes in value for the sure gains in both studies. The findings also indicated that both anticipatory and anticipated emotions explained the effects of the value on choice for the sure gain and sure losses, while both mediated the effect of framing on choice towards the sure and the gamble option. Although anticipatory emotions mediated a larger portion of the effect, anticipated emotions also show some mediation.

**Supplementary Information:**

The online version contains supplementary material available at 10.1007/s00426-024-02001-3.

## Introduction

Research in decision-making has predominantly adopted a consequentialist view; individuals are assumed to choose the course of action with the most beneficial expected outcome. In standard economic models, for instance, choices are conceived to be determined by the weighted probability of monetary outcomes (Loewenstein & Lerner, 2003; Von Neumann & Morgenstern, 1944).

Tversky and Kahneman (1981) suggested that the selection between varying gains and losses reflects an individual’s emotional response to these alternatives, with losses evoking stronger negative emotions than equivalent gains evoke positive emotions. More recent studies that integrated emotions into these models have also remained consequential in their approach (Cheng et al., 2022). These studies have showed that the emotions people expect to feel as a consequence of their decisions, the so-called anticipated emotions (Bagozzi et al., 2003; Loewenstein & Lerner, 2003; Mellers et al., 1999), play a significant role in decision-making (Bell, 1982, 1985; Dorison et al., 2020; Duxbury et al., 2020; George & Dane, 2016; Hillebrandt & Barclay, 2017; Mellers et al., 1997; Rutledge et al., 2014; Zaleskiewicz & Traczyk, 2020). Regret Theory, developed by Loomes and Sugden (1982) suggests that people make decisions not just based on the outcomes but also on the regret they anticipate feeling if they make the wrong choice. This theory highlights the powerful role of anticipated regret in driving individuals toward options that minimize potential future regret, even if those options involve more risk. In addition, Psychological Expected Utility Theory provides a comprehensive framework, incorporating both anticipatory and anticipated emotions into the evaluation of choices, explaining how these emotions mediate the framing effect (Caplin & Leahy, 2001). The goal of this study is to add to our understanding of ways that emotions influence decision-making by considering how people feel in the moment about the possible outcomes of their choices and not only how they expect to feel in the future because of their decisions. According to Simon (1983), “*in order to have anything like a complete theory of human rationality*,*we have to understand what role emotion plays in it.*” (Simon, 1983, p. 29).

To fully understand emotions and decision-making, we must move beyond consequentialist frameworks. Consequentialist frameworks allow us to uncover crucial processes underlying decision-making, however, they do not capture all the ways emotions influence decisions. Existing literature (Loewenstein & Lerner, 2003; Loewenstein et al., 2001) highlights that decisions would also be impacted by “anticipatory emotions” which are immediate emotions that arise while contemplating about the decision alternatives. Both anticipatory and anticipated emotions are about the decisions in hand, strongly and routinely shaping decision making, so they are together called “integral emotions” (Damasio & Sutherland, 1994; Dorison et al., 2020; Duxbury et al., 2020; George & Dane, 2016; Hillebrandt & Barclay, 2017; Lerner et al., 2015; Zaleskiewicz & Traczyk, 2020). For example, a person who feels anxious about the potential outcome of a risky choice may choose a safer option rather than a potentially more lucrative option. In particular, a few philosophers pioneered the idea that integral emotion could be a beneficial guide, as, for example, anticipation of regret provides a reason to avoid excessive risk-taking (George & Dane, 2016; Lerner et al., 2015). However, integral emotions can also bias decision making. For example, one may feel afraid to fly and decide to drive instead, even though flying on a plane is overwhelmingly safer than driving a car (Gigerenzer, 2004).

Yet, while anticipated emotions are judgments about some future experiential state that will happen as a consequence of the decision (e.g., anticipated joy or regret), anticipatory emotions are actually experienced in the moment as people are contemplating decision alternatives (e.g., hope or fear), so these are conceptually and empirically distinct in terms of uncertainty, range and phenomenology of the emotion (Baumgartner et al., 2008; Loewenstein & Lerner, 2003). A few studies have compared anticipatory and anticipated emotions and found that anticipated emotions have a stronger effect in avoiding negative outcomes of the millennium transition than anticipatory emotions (Baumgartner et al., 2008; Xu & Guo, 2019). However, due to a limited number of studies, we do not know whether anticipatory emotions or anticipated emotions have different effects in the decision-making process and whether they have different levels of impacts on various kinds of behaviours.

It is important to understand the relationship between anticipatory and anticipated emotions on decision-making because these relationships underlie theories of behaviour change and influence. Such theories contribute to shaping behaviour in ways that may generate more health and wellbeing. Prominent approaches to behaviour change include the behaviour change wheel, which focusses on people’s capabilities, opportunities, and motivations to change; emotions are an aspect of motivation (Michie et al., 2011). Emotions respond to outcomes in ways that are not consistent or proportional to the outcome, as people react more strongly to losses than gains (Kahneman & Tversky, 1979; Dolan et al., 2012; Vlaev et al., 2019). Understanding the interplay of emotions with decisions about losses can significantly improve behavioural interventions. Overall, the purpose this study is to examine if anticipatory and anticipated emotions have differential relationships with the decision-making process according to outcome values.

Current studies have examined whether anticipatory emotions have predictive power in financial decision-making above and beyond anticipated emotions. Schlösser et al. (2013) asked samples of students “How do you feel right now about choosing alternative X?” before they made a choice between a $5 and a 50% chance to win $10 with a coin flip (with the roll of a die in a second study). Results showed anticipatory emotions predicted choice independent of the anticipated emotions (“How would you feel when the decision for alternative X leads to consequence Y?”) and the subjective probability attached to outcomes. This was the first empirical study to demonstrate the unique predictive ability of anticipatory emotions in decision-making under risk, but since the outcomes were held constant, it didn’t offer an understanding of how the predictive power of emotions holds under different outcome values and alternative framings of the outcomes as gain and loss.

In an additional study, Young et al. (2019) have researched the comparative role of anticipatory and anticipated emotions when outcomes are framed as gain and loss. They endowed students with money ($25 to $100) and asked each participant to make choices between a sure (riskless) gain/loss of money and a gamble option where the expected outcome is equivalent to the sure money (e.g., 80% chance of keeping all and 20% chance of losing all). In within-subjects’ conditions, the sure option was framed as a gain or a loss. In line with earlier studies (Cheung & Mikels, 2011; Stark et al., 2017), the authors find that framing affected anticipatory emotions and these emotions in turn mediated the effect of framing on choice. Anticipated emotions, on the other hand, didn’t play a significant role in explaining the relationship between framing and choice.

Existing literature provides suggestive evidence that anticipatory emotions have a unique predictive power in risky decision-making (Baumgartner et al., 2008; Schlösser et al., (2013). However, it is not yet clear what gives rise to anticipatory emotions. Baumgartner et al. (2008) described anticipatory emotions as a direct product of risk and raise the possibility that these emotions would arise in response to the probabilities of the events whereas anticipated emotions would be more closely linked to outcomes as they correspond to a time where the risk is resolved and outcomes are experienced. Similar to this, Schlösser et al. (2013) found that changes in objective risk affect anticipatory emotions. Another important component of decisions, outcome values, have not yet received empirical attention as a determinant of anticipatory emotions. Loewenstein and Lerner (2003) stressed that outcomes (as opposed to risk) would be important determinant of anticipatory emotions in comparison to anticipated emotions. The study also refers to Damasio and Sutherland (1994), who proposed that emotions arise in response to the mental images of an outcome. Indeed, studies have shown that anticipated emotions change as a function of outcome values (Charpentier, 2016; Charpentier et al., 2016). However, no study, to our knowledge, have examined the influence of outcome values on anticipatory emotions.

### Present research

In the current study, we empirically investigated whether and how anticipatory and anticipated emotions may change as a function of outcome values and whether anticipatory or anticipated emotions may explain the influence of outcome values on risky choice. While there is no standard definition, the term “risky choice” in the current experiment is empirically defined as a choice between two options, a guaranteed “sure option” and a risky “gamble option, exemplified by Prospect-Theory (Kahneman & Tversky, 1979). Risky-choice behaviour has garnered significant attention from researchers in various fields, particularly in behavioural psychology and economics, where individuals must select between a certain option and an uncertain one (Cheng et al., 2022). It is important to emphasize the differentiation between decision-making under uncertainty and decision-making under risk within psychology and related fields (De Groot & Thurik, 2018). Neglecting to make this distinction appropriately could potentially result in researchers drawing misleading or inaccurate conclusions. In economics, the differentiation between uncertainty and risk initially proposed by Knight (1921). Under the concept of risk, the outcome is unknown, but the probability distribution governing that outcome is known. In contrast, uncertainty is characterized by both an unknown outcome and an unknown probability distribution. In both scenarios, preferences are established based on the probability distributions of outcomes. For risk, these probabilities are considered objective, while for uncertainty, they are subjective. Furthermore, behavioural economics literature underscores a prevailing aversion to uncertainty as compared to risky choices, a phenomenon often termed “ambiguity aversion.” Individuals tend to favour known probabilities over unknown ones, even when the known probability is low, and the unknown probability could potentially result in a guaranteed win, as demonstrated by Ellsberg (1961). The psychological literature also supports the empirical distinction between uncertainty and risk. For instance, Buckert et al. (2014) illustrated that the cortisol response to stress influences decision-making under risk but not under uncertainty.

To study the effects of value on emotions and choice, we offered people hypothetical large amounts ($100, $200, $300, and $400) and incentivized moderate amounts ($10, $20, $30, $40) as prospects in gambles over two consecutive studies. We conducted the two studies to ensure our results were incentive-compatible and assess any differences according to monetary amount. The sure option was calculated using a utility function that accounts for different levels of risk preferences. Given prior evidence that stressed the importance of anticipatory emotions in gain and loss domain (Cheung & Mikels, 2011; Stark et al., 2017; Young et al., 2019), we had each participant in our study to make choices under gain and loss domains. Finally, prior studies employed only student samples (Davis et al., 2009; Kocher et al., 2014; Schlösser et al., 2013), whereas we used a nationally representative sample to test the generalizability of the findings. Both studies were approved by the University of Warwick’s ethics committee.

## Study 1 – hypothetical large choices

### Method

#### Participants

**Fig. 1:**
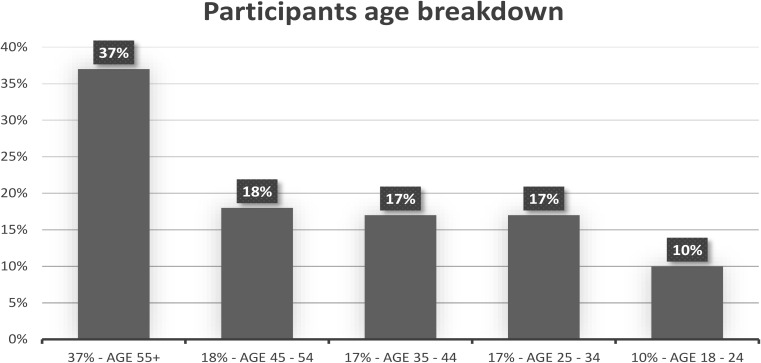
Visual depiction of the participants age breakdown

**Fig. 2:**
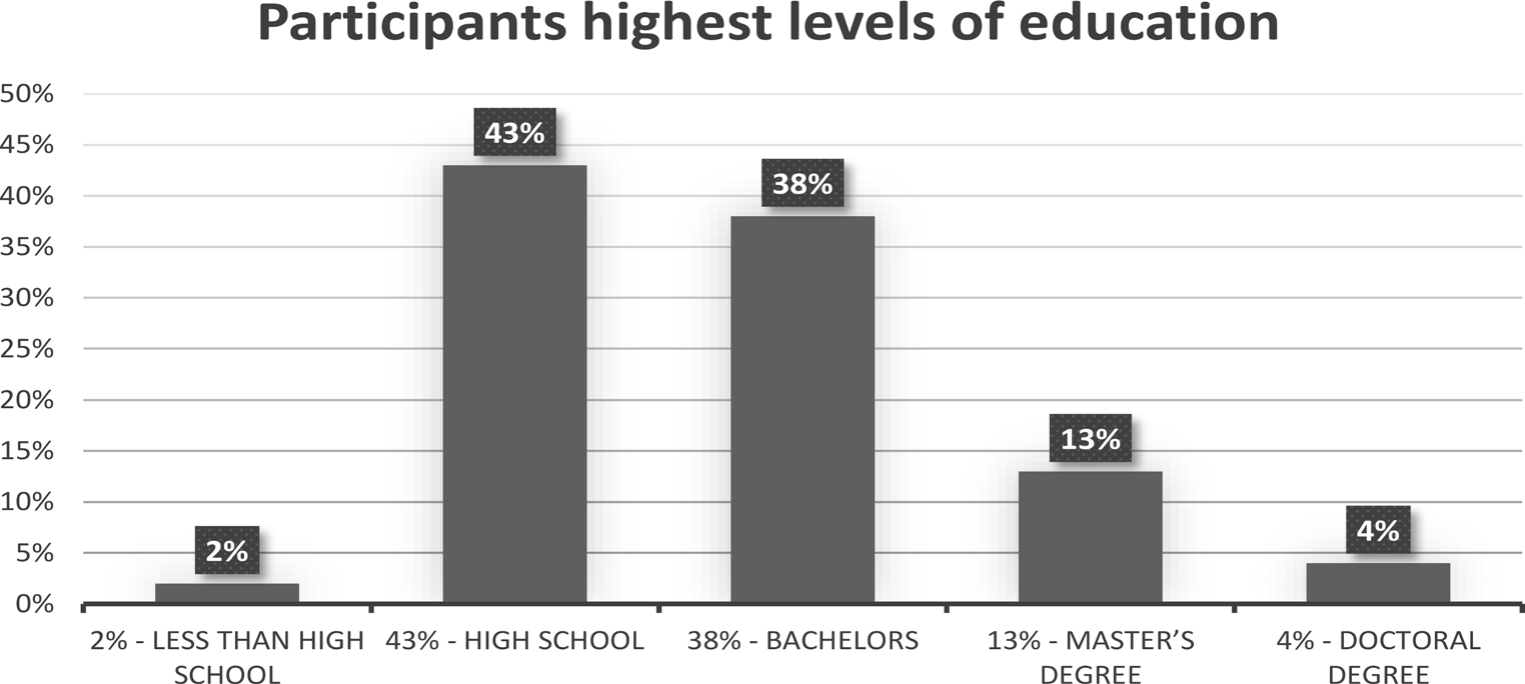
Visual depiction of the highest levels of education

In total, 311 adults from the US took part in the study. The recruitment of research participants was conducted by Qualtrics. Participants were screened based on age, gender and education to maintain representativeness of the population. Gender was equally split in our sample. Age breakdown was 37% for ages 55+, 18% for ages between 45 and 54, 17% for ages between 35 and 44, 17% for ages between 25 and 34, and 10% for ages between 18 and 24 (see Fig. 1). The highest levels of education completed were less than high school for 2% of the sample, high school for 43%, bachelors for 38%, Master’s degree for 13% and doctoral degree for 4% (see Fig. 2).

#### Design

**Fig. 3:**
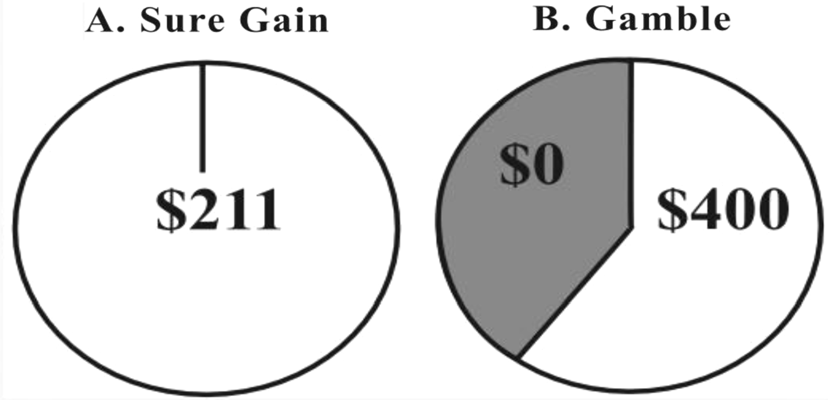
Visual depiction of the risky bet indicating the two probabilities for gain versus nothing, respectively

An online survey was designed, in which participants made choices between a sure thing and a risky option (p chance of x). Each pair of options was presented as two pie charts. The two regions of the pie chart represented the risky bet indicating the two probabilities for gain versus nothing, respectively (see Fig. 3). Such gambles are widely used to measure risk aversion in most laboratory settings (Charness et al., 2013).

The risky option was constructed by crossing four levels of probability (0.2, 0.4, 0.6, and 0.8) and four levels of prospect ($100, $200, $300, $400) to create 16 choices. The sure amount was generated using a function with four levels of power γ (gamma) (0.35, 0.50, 0.65, 0.80) as they were observed in previous studies (Vlaev et al., 2010). A person with power γ would be indifferent between the sure thing and the risk. In particular, we used the following equation:

y = x p^1/γ^, (1)

where y is the sure amount and the prospect is a “p chance of x.” γ describes the curvature of a hypothetical power law utility function, u(x) = xγ. Gamma is equal to one for a risk-neutral person. Smaller values of γ denote greater risk aversion. It is selected for its theoretical underpinning in Constant Relative Risk Aversion (CRRA), a fundamental concept in economic decision-making under uncertainty. CRRA utility functions assume that individuals’ risk preferences remain proportional to their wealth levels, providing a structured framework to analyze risk-taking behaviour across different economic contexts. Empirical studies frequently support the validity of CRRA functions in explaining how individuals balance risks and rewards, particularly in financial decision-making. The parameter 𝛾 determines the curvature of the utility function, influencing the sensitivity of individuals to changes in wealth or outcomes. This mathematical form allows researchers to simulate and predict behaviour in uncertain environments, offering insights into how risk preferences shape economic outcomes.

Four levels of γ were used (0.35, 0.50, 0.65, and 0.80) so that participants in the middle of the risk-aversion continuum will choose a mixture of sure amounts and risky prospects. As might be expected, very risk-averse individuals will choose only the sure amounts and very risk-seeking persons would choose only the prospects. Levels of γ were randomly assigned to gambles with the constraint that each level of γ occurred once for each amount and once for each probability. A set of 64 gambles (4 × 4 × 4) would be needed to map the whole surface of possible combinations between the four levels of probability, prospect amount, and γ. However, asking 64 questions to each person would induce a significant respondent burden so we randomly assigned the combinations to 4 groups that constitute 16 combinations each. Participants in our study were randomly allocated to these 4 groups. We made sure that all four levels of γ paired with every monetary amount and probability. We also used four different orders of the four γ levels across the 16 gambles.

#### Procedure

The same 16 gambles framed as gains and losses were presented to the participants. Participants saw gain and loss blocks in a random order. “Gain” gambles asked the participants to make imaginary choices between a sure gain and an option that gave a chance to gain another amount, and “loss” gambles involved imaginary choices between a sure loss and an option that gave a chance to lose another amount.

After each decision, participants answered questions in blocks related to anticipatory emotions and anticipated emotions and the order of presentation for these blocks were randomized. The anticipatory emotions block consisted of 2 questions: “How do you feel right now about choosing the sure option?” and “How do you feel right now about choosing the gamble?”. Anticipated emotions block consisted of 3 questions: “How would you feel if you have chosen the “sure option” and received (or lost) $50?” “How you would you feel if you have chosen the “gamble” and got $0?” and “How you would you feel if you have chosen the gamble and won (or lost) $10?” After each emotion question, respondents rated first the valence of their feelings from − 4 to 4 and the intensity of their feelings from 1 to 7. Lastly, the participants were instructed to answer as they would answer if they were making these decisions for real. The lotteries are presented in Supplemental Materials.

### Results

#### The effects of outcome values on emotions

A within-person fixed effects linear regression model was used for estimating each effect. This model enables us to compare within-person emotion ratings as a function of values. When we are estimating the effects of sure dollars on emotions to the sure option, we control for gamble dollars and risk, and when estimating the effects of gamble dollars on the emotions to the gamble option, we control for sure dollars and risk. Errors are clustered at the individual level to account for the correlations in errors for repeated observations per person.

##### Anticipatory emotions

**Fig. 4:**
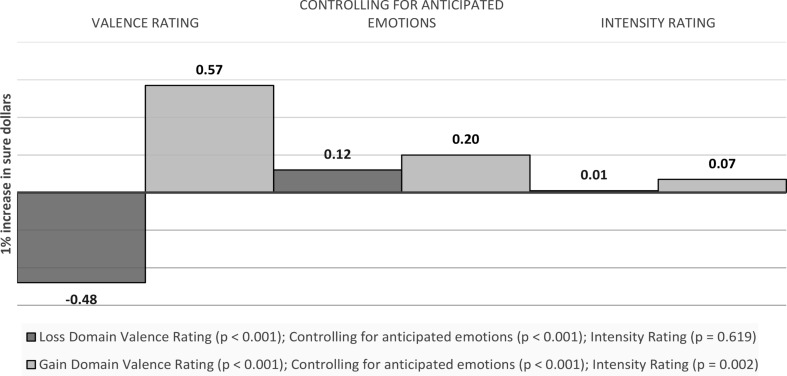
Visual depiction of the effects of outcome values on emotions: Anticipatory emotions - Sure Money

Sure money: A 1% increase in sure dollars is associated with a 0.48 decrease in the valence ratings of anticipatory emotions towards the sure option in the loss domain (*p* < 0.001), and a 0.57 increase in the gain domain (*p* < 0.001).^1^ Controlling for anticipated emotions towards the sure option, the effect becomes 0.12 (*p* < 0.001) and 0.20 (*p* < 0.001) respectively (around three quarters and two thirds smaller respectively). For intensity ratings, the effect is not significant in the loss domain (b = 0.01, *p* = 0.619) and point to a 0.07 (*p* = 0.002) increase in the gain domain (see Fig. 4).

**Fig. 5:**
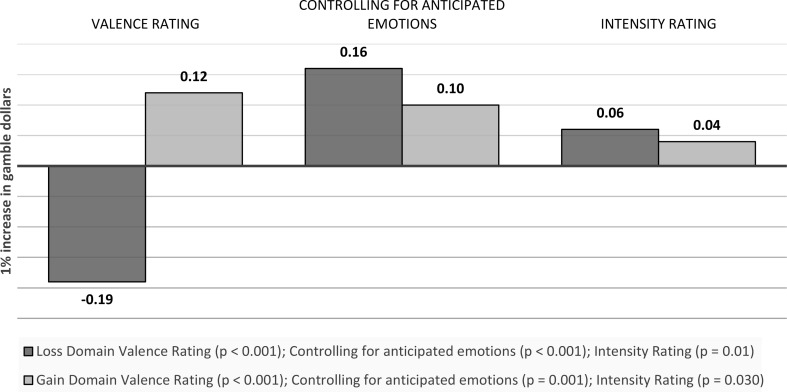
Visual depiction of the effects of outcome values on emotions: *Anticipatory emotions - Gamble Money*

Gamble money: A one unit increase in gamble dollars is associated with a 0.19 (*p* < 0.001) decrease in the valence ratings of anticipatory emotions towards the gamble option in the loss domain, and a 0.12 (*p* < 0.001) increase in the gain domain. Controlling for anticipated emotions, the effect becomes 0.16 (*p* < 0.001) and 0.10 (*p* = 0.001) respectively. For intensity ratings, the effect is 0.06 in the loss domain (*p* = 0.01) and 0.04 in the gain domain (*p* = 0.030) (see Fig. 5).

##### Anticipated emotions

**Fig. 6:**
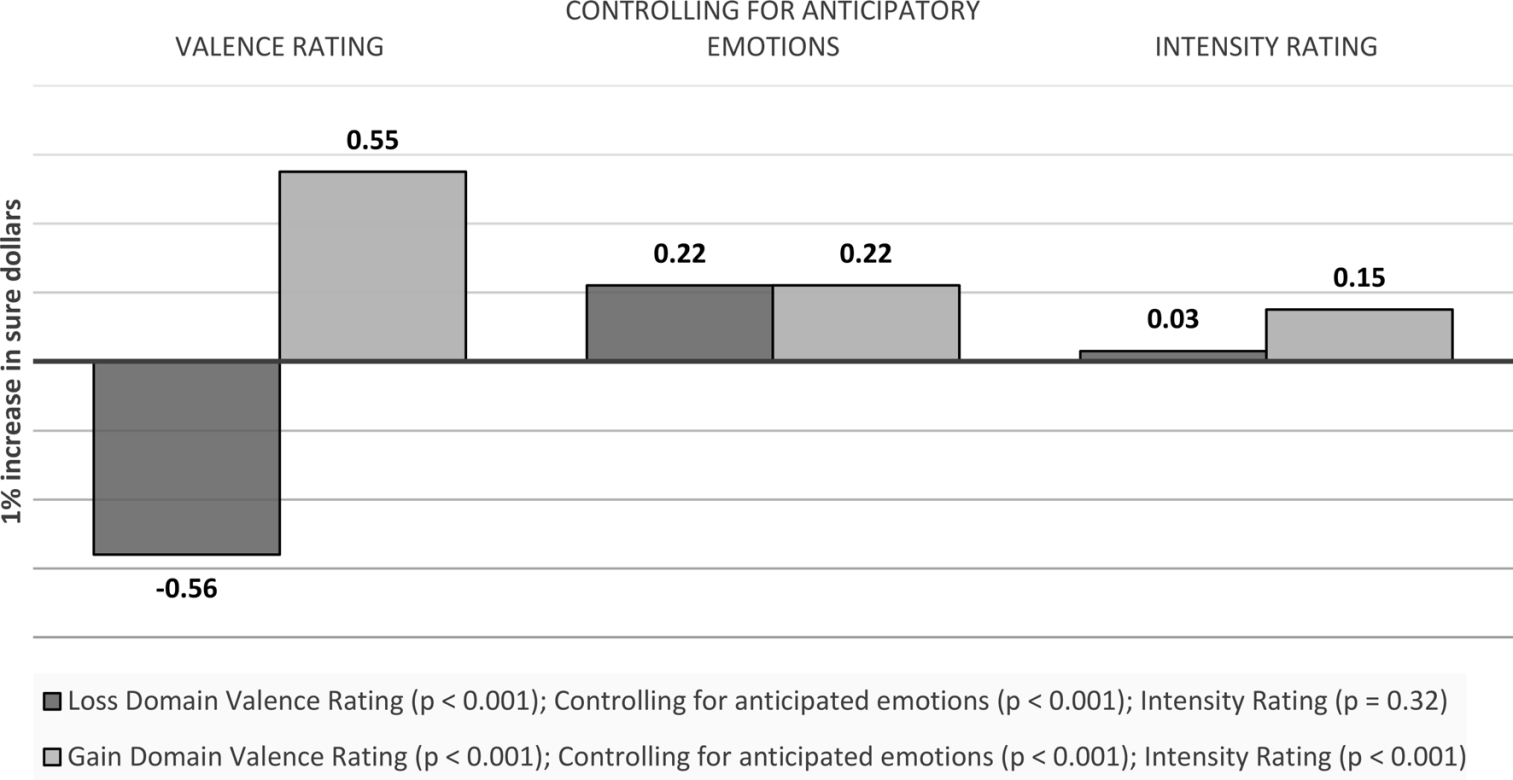
Visual depiction of the effects of outcome values on emotions: Anticipated emotions - Sure Money

Sure money: A 1% increase in sure dollars is associated with a 0.56 decrease in the valence ratings of anticipated emotions towards the sure option in the loss domain (*p* < 0.001), and a 0.55 increase in the gain domain (*p* < 0.001). Controlling for anticipatory emotions, the effect becomes 0.22 (*p* < 0.001) and 0.22 (*p* < 0.001) respectively (around one fifth and two thirds smaller respectively). For intensity ratings, the effect is not significant in the loss domain (b = 0.03, *p* = 0.32) and point to a 0.15 increase in the gain domain (*p* < 0.001) (see Fig. 6).

**Fig. 7:**
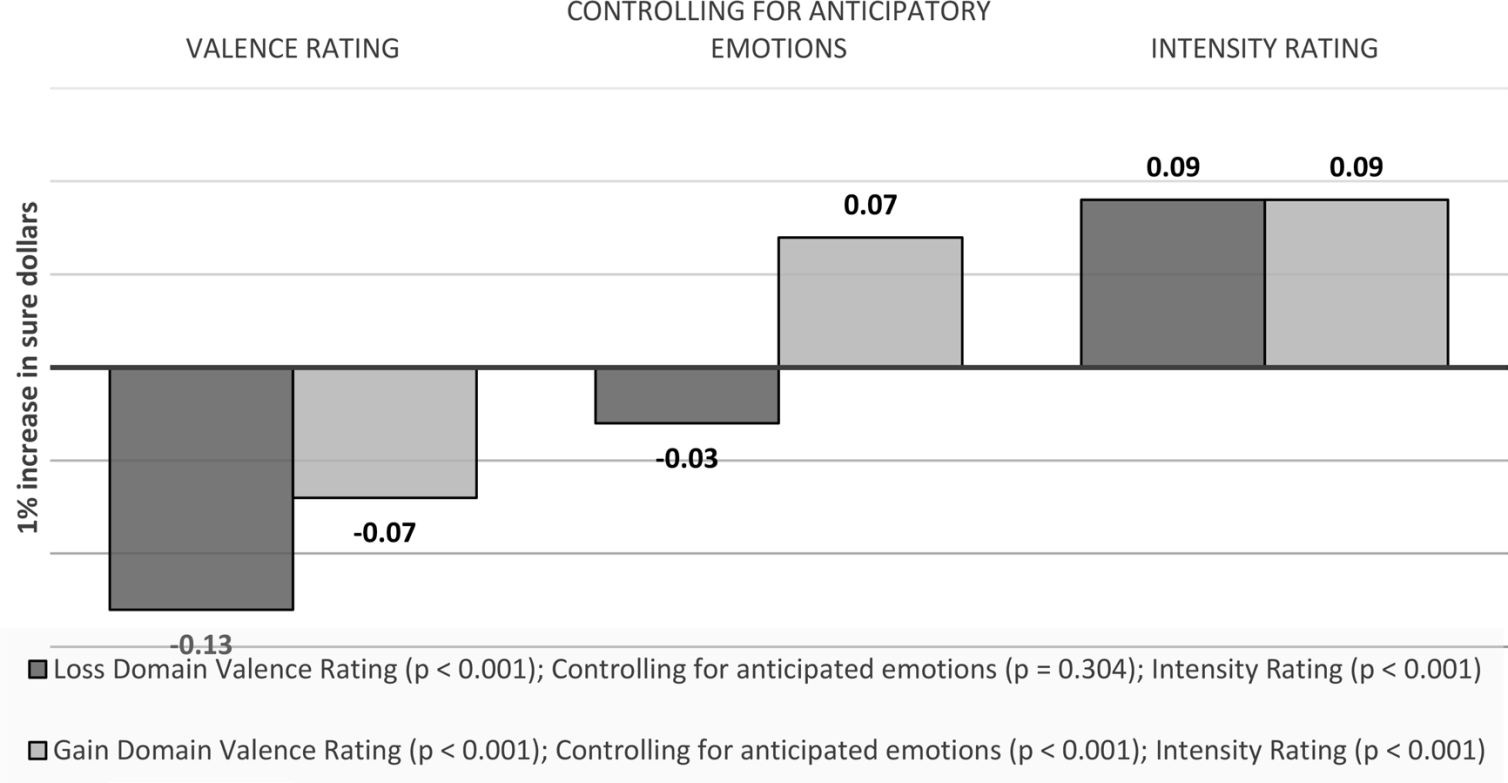
Visual depiction of the effects of outcome values on emotions: *Anticipated emotions - Gamble Money*

Gamble money. A one unit increase in gamble dollars (which equals a $100 change) is associated with a 0.13 (*p* < 0.001) decrease in the valence ratings of anticipated emotions towards the gamble option in the loss domain, and a 0.07 (*p* < 0.001) decrease in the gain domain. Controlling for anticipatory emotions, the effect becomes insignificant for loss domain (b = -0.03, *p* = 0.304) and remains 0.07 (*p* < 0.001) in the gain domain. For intensity ratings, the effect is a 0.09 increase in both gain and loss domain (*p* < 0.001) (see Fig. 7).

#### Emotions as a mediator of outcome values’ effects on choice

For mediation analyses, we first demeaned the variables in order to retrieve estimates identical to a fixed-effects model in a regression analysis. We did this in order to be able to employ a multiple mediation analysis (Preacher & Hayes, 2008) using the “sureg” command in Stata. We controlled for risk (probability) and monetary values in all models. In estimating the mediating effects on choice, the model was a within-person fixed effects linear probability model (LPM) where dependent variable measures the probability of choosing the risky gamble (0 ≤ *p* ≤ 1) and was coded 1 if the choice was the risky gamble and 0 if the choice was the sure amount. Note that the estimated effects and significance levels are practically the same in linear probability models as in a logistic regression. However, the ease of interpreting the coefficients is considerably easier with the LPM (Hellevik, 2009). For ease of interpreting the results and for being able to conduct the mediation analyses, we split the data to gain and loss for these analyses instead of using interaction terms for each predictor and covariate.

**Fig. 8:**
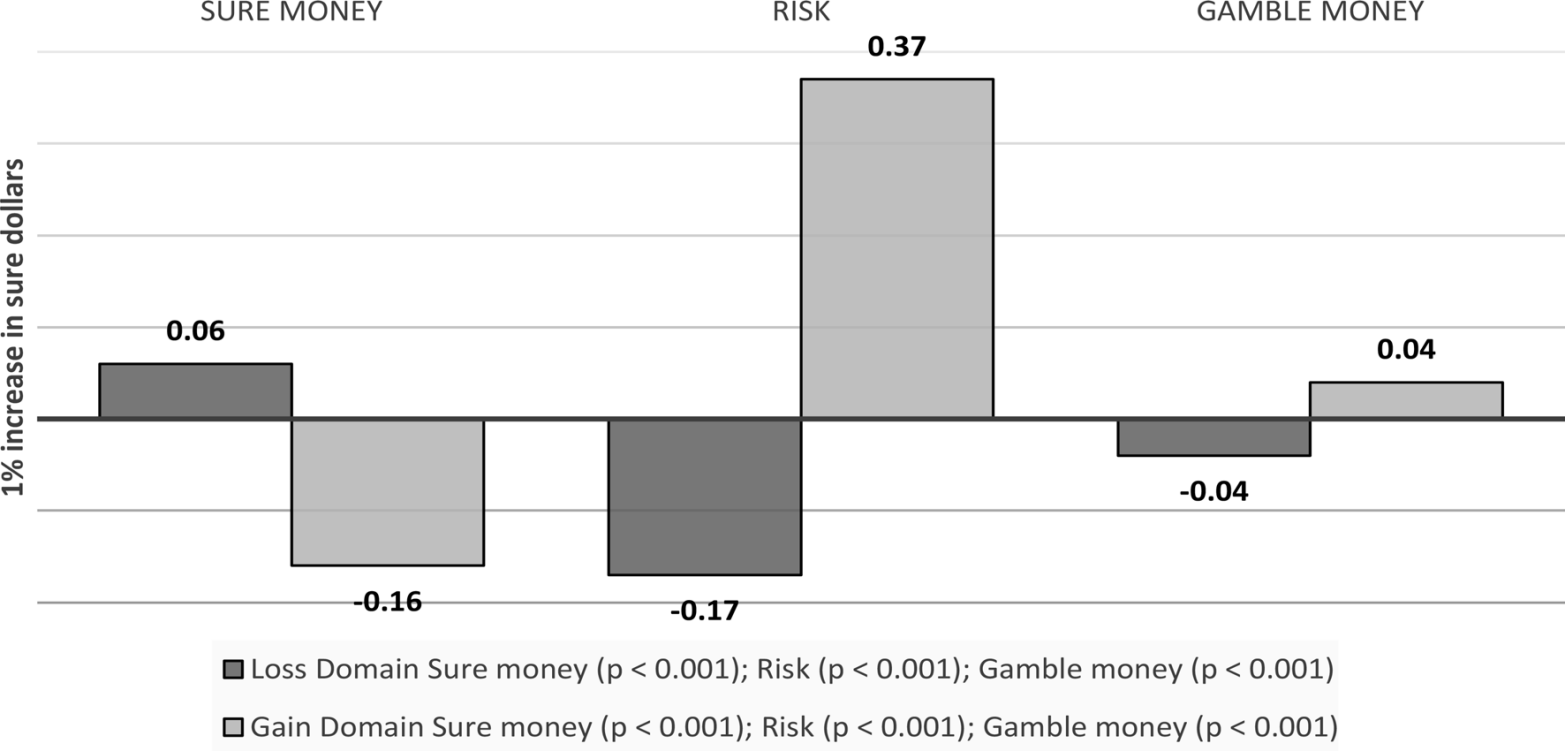
Visual depiction of the emotions as a mediator of outcome values’ effects on choice

Initial analyses confirmed that in the gain domain, (log) sure dollars (b = -0.16, *p* < 0.001), gamble dollars (b = 0.04, *p* < 0.001) and risk (b = 0.37, *p* < 0.001) were all significant predictors of risky choice. In the loss domain, (log) sure dollars (b = 0.06, *p* < 0.001), gamble dollars (b = -0.04, *p* < 0.001) and risk (b = -0.17, *p* < 0.001) were all significant predictors of risky choice (see Fig. 8).

##### Anticipatory emotions

Sure money. In multiple mediation models, we tested the mediation effects of all emotions related to the sure option simultaneously. Results revealed that, in the gain domain, anticipatory emotions towards the sure option mediated the effects of sure dollars on choice (Indirect effect: -0.04, *p* < 0.001). In the loss domain, anticipatory emotions towards the sure option mediated the effects of sure dollars on choice (Indirect effect: 0.02, *p* < 0.001).

**Fig. 9:**
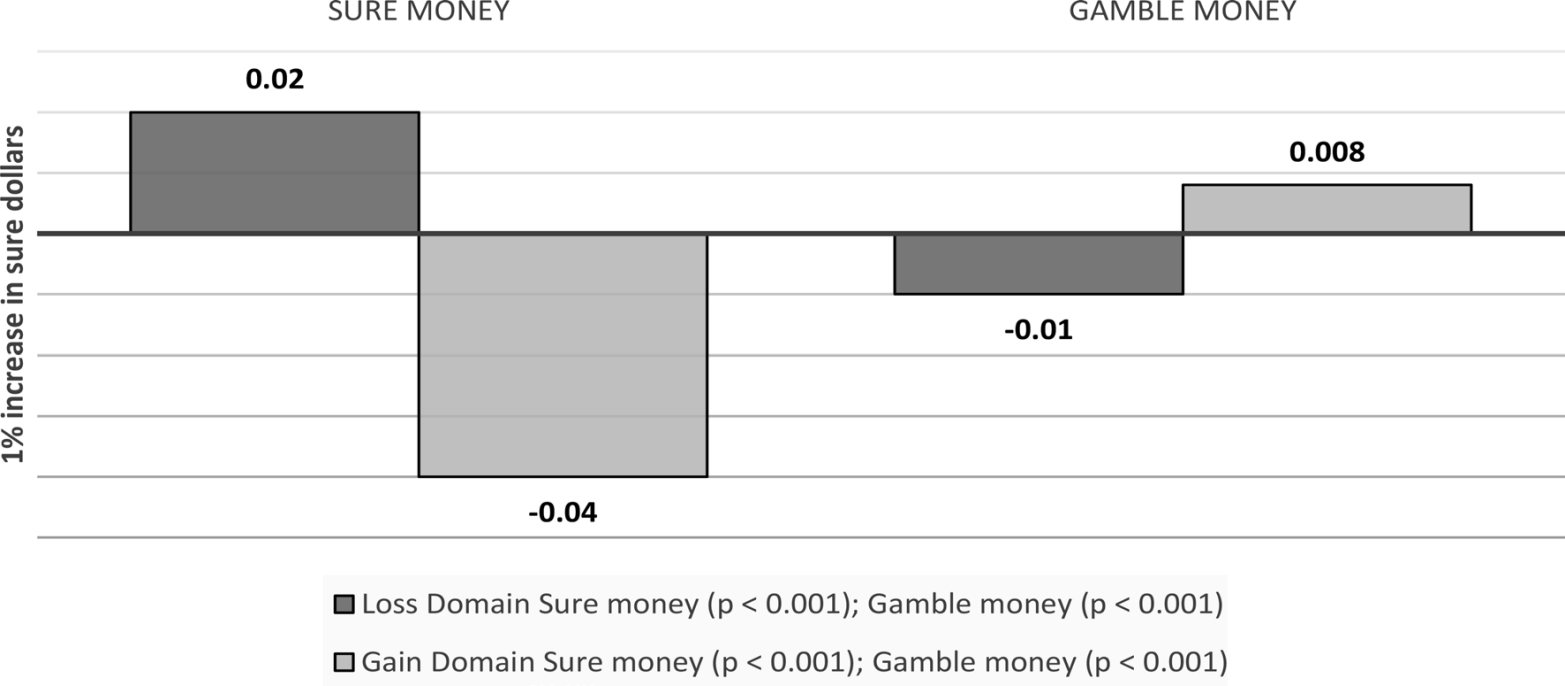
Visual depiction of the emotions as a mediator of outcome values’ effects on choice

Gamble money. In the gain domain, anticipatory emotions towards the gamble option mediated the effects of gamble dollars on choice (Indirect effect: 0.008, *p* < 0.001). In the loss domain, anticipatory emotions towards the gamble option mediated the effects of gamble dollars on choice (Indirect effect: -0.01, *p* < 0.001) (see Fig. 9).

##### Anticipated emotions

Sure money. In the gain domain, anticipated emotions towards the sure also mediated the effects of gamble dollars on choice (Indirect effect: -0.02, *p* < 0.001). In the loss domain, anticipated emotions towards the sure also mediated the effects of gamble dollars on choice (Indirect effect: 0.01, *p* < 0.001).

**Fig. 10:**
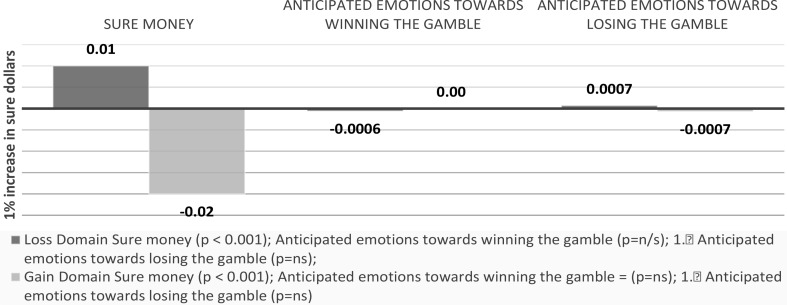
Visual depiction of the emotions as a mediator of outcome values’ effects on choice

Gamble money. In the gain domain, anticipated emotions towards winning the gamble didn’t significantly mediate the effects of gamble dollars on choice (indirect effect: 0.00, *p* = 0.600), and mediation for anticipated emotions towards losing the gamble was not statistically either (indirect effect: -0.0007, *p* = 0.069). In loss, anticipated emotions towards winning the gamble (and losing the prospect amount) didn’t significantly mediate the effects of gamble dollars on choice (indirect effect: -0.0006, *p* = 0.352), and anticipated emotions towards losing the gamble (and not losing the prospect amount) was only not significant either (indirect effect: 0.0007, *p* = 0.089) (see Fig. 10).

#### Emotions as a mediator of gain and loss domain effect on choice

We find that the same individual is more risk-seeking when making choices in the loss domain than gain (b = 0.05, *p* = 0.001). We then tested whether emotions towards the sure option could explain the effect of framing on choice using a multiple mediation model where anticipatory and anticipated emotions’ mediation effects could be examined simultaneously. The loss condition increases risk-seeking indirectly via anticipatory emotions towards the sure option (indirect effect: 0.11, *p* < 0.001) as well as anticipated emotions towards the sure option (indirect effect: 0.04, *p* < 0.001).

Emotions towards the gamble, however, yielded an opposite influence on risk-taking. Anticipatory emotions towards the gamble was lower in loss domain than gain, which in turn, decreased risk taking (indirect effect: -0.09, *p* < 0.001). The results were the same for anticipated emotions towards winning the gamble (and receiving the prospect) (indirect effect: -0.05, *p* < 0.001). Anticipated emotions towards losing the gamble did not have significant mediation effects (indirect effect: 0.02, *p* = 0.147).

## Study 2 – incentivized moderate choices

In Study 1, we looked at hypothetical large choices. In Study 2, we looked at incentivized moderate choices. Before commencing the experiment, participants were briefed on the payments for the follow-up experiment. Each participant faced a two-step process where a random question from both the gain and loss domains was selected, which had the potential to be played out for real. Another random selection determined whether the participant’s choices would be enacted from the gain or loss domain. In cases where participants chose the gamble over the sure payoff, a random number was generated based on given probabilities to determine the final outcome. If the gain domain was chosen, participants received the amount they selected in the randomly chosen question from the gain domain. In the event that a choice from the loss domain was randomly selected to be played out for real, participants were compensated with the amount they would have earned in the gain domain minus the loss amount. If the resulting difference was below 0, participants received no payment. Participants were explicitly informed that in the loss domain, they would experience a deduction from the money they could have earned in the gain domain. The purpose of study to is to investigate if our results are incentive-compatible and assess any differences according to monetary amount.

### Method

#### Participants

**Fig. 11:**
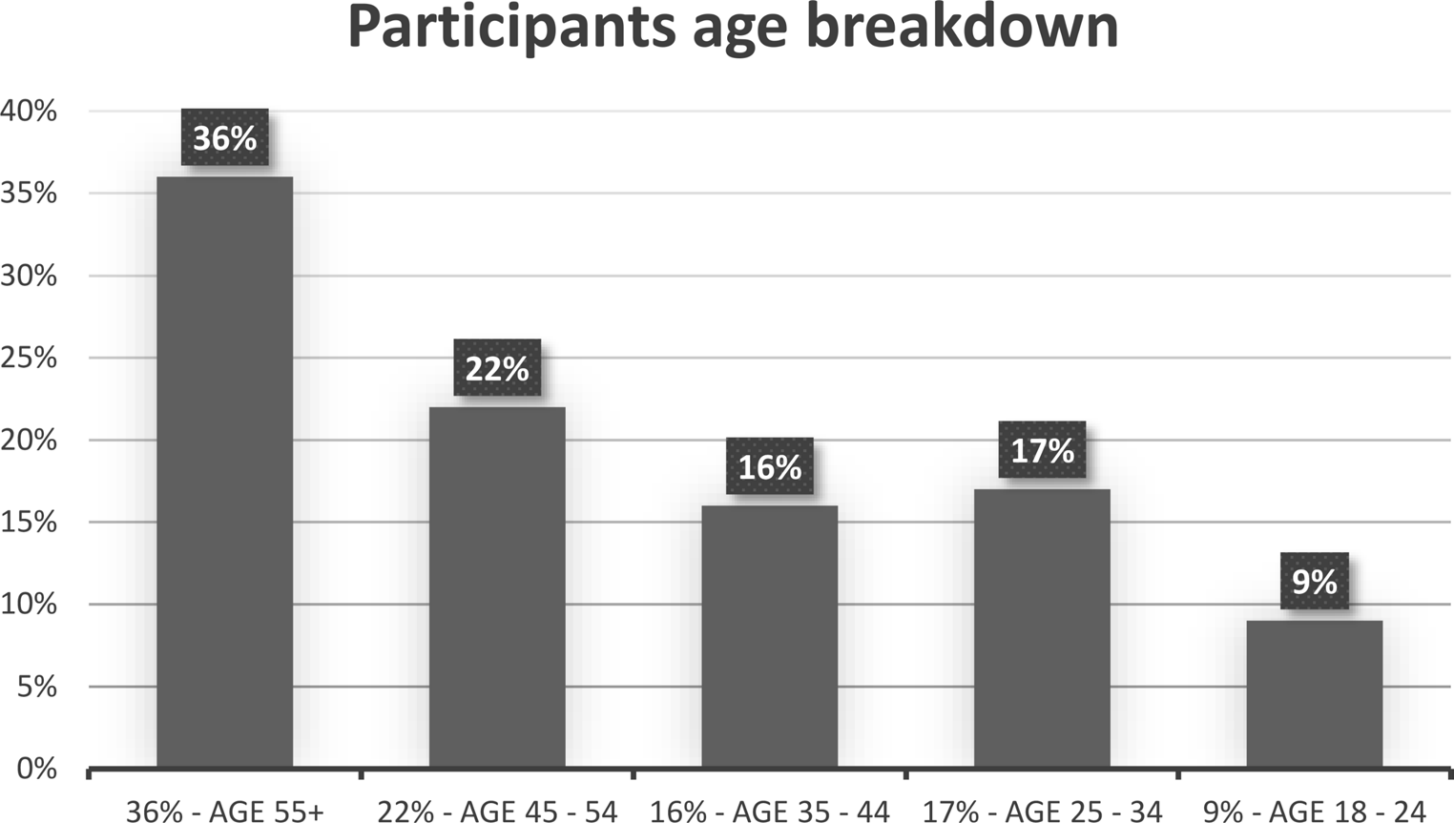
Visual depiction of the participants age breakdown

In total, 268 adults from the US took part in the study and the recruitment was conducted by Qualtrics. The sampling procedure involved screening based on age, gender and education to maintain representativeness of the population. Gender was equally split. Age breakdown was 36% for ages 55 or above, 22% for ages between 45 and 54, 16% for ages between 35 and 44, 17% for ages between 25 and 34, and 9% for ages between 18 and 24 (see Fig. 11).

**Fig. 12:**
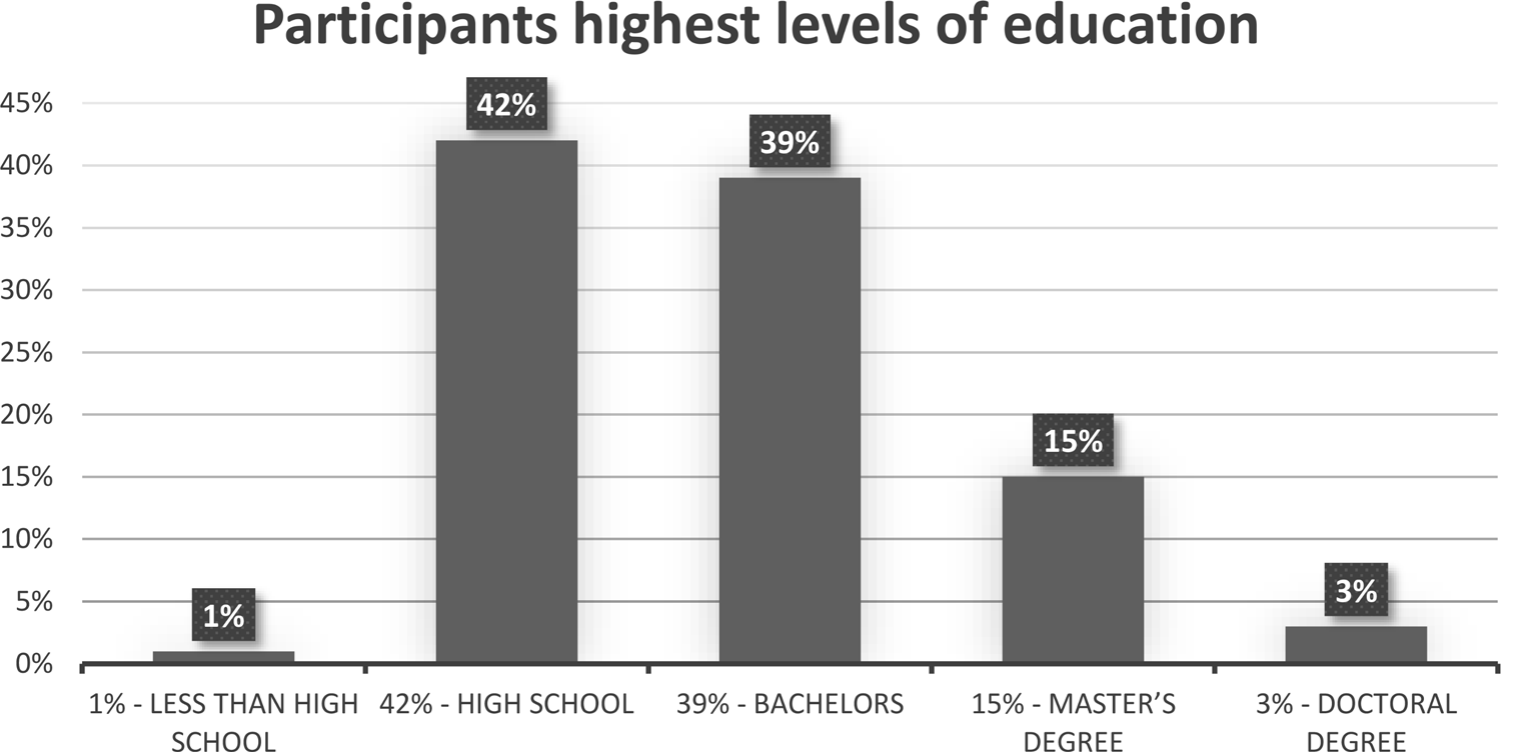
Visual depiction of the highest levels of education

The highest levels of education completed were less than high school for 1% of the sample, high school for 42%, bachelors for 39%, Master’s degree for 15% and doctoral degree for 3% (see Fig. 12).

#### Design and procedure

The design and the procedure were identical with the following slight differences. The risky option was constructed by crossing four levels of probability (0.2, 0.4, 0.6, and 0.8) and four levels of prospect ($10, $20, $30, $40) to create 16 choices. The participants were notified that one of their choices will be played out for real. We made payments to the participants accordingly. Given resource constraints, we only asked about valence and not intensity in the second study.

### Results

#### The effects of outcome values on emotions

##### Anticipatory emotions

**Fig. 13:**
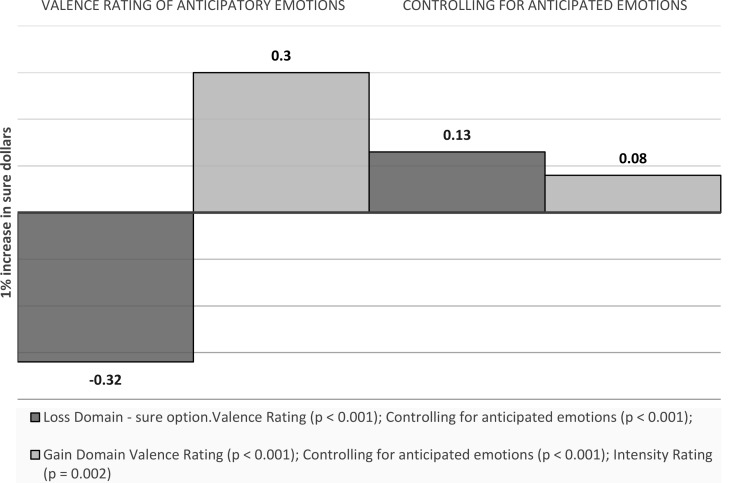
Visual depiction of the effects of outcome values on emotions

Sure money: A 1% increase in sure dollars is associated with a 0.32 decrease in the valence ratings of anticipatory emotions towards the sure option in the loss domain (*p* < 0.001), and a 0.30 increase in the gain domain (*p* < 0.001). Controlling for anticipated emotions towards the sure option, the effect becomes 0.13 (*p* < 0.001) and 0.08 (*p* < 0.001) respectively (around two thirds and three quarters smaller respectively) (see Fig. 13).

**Fig. 14:**
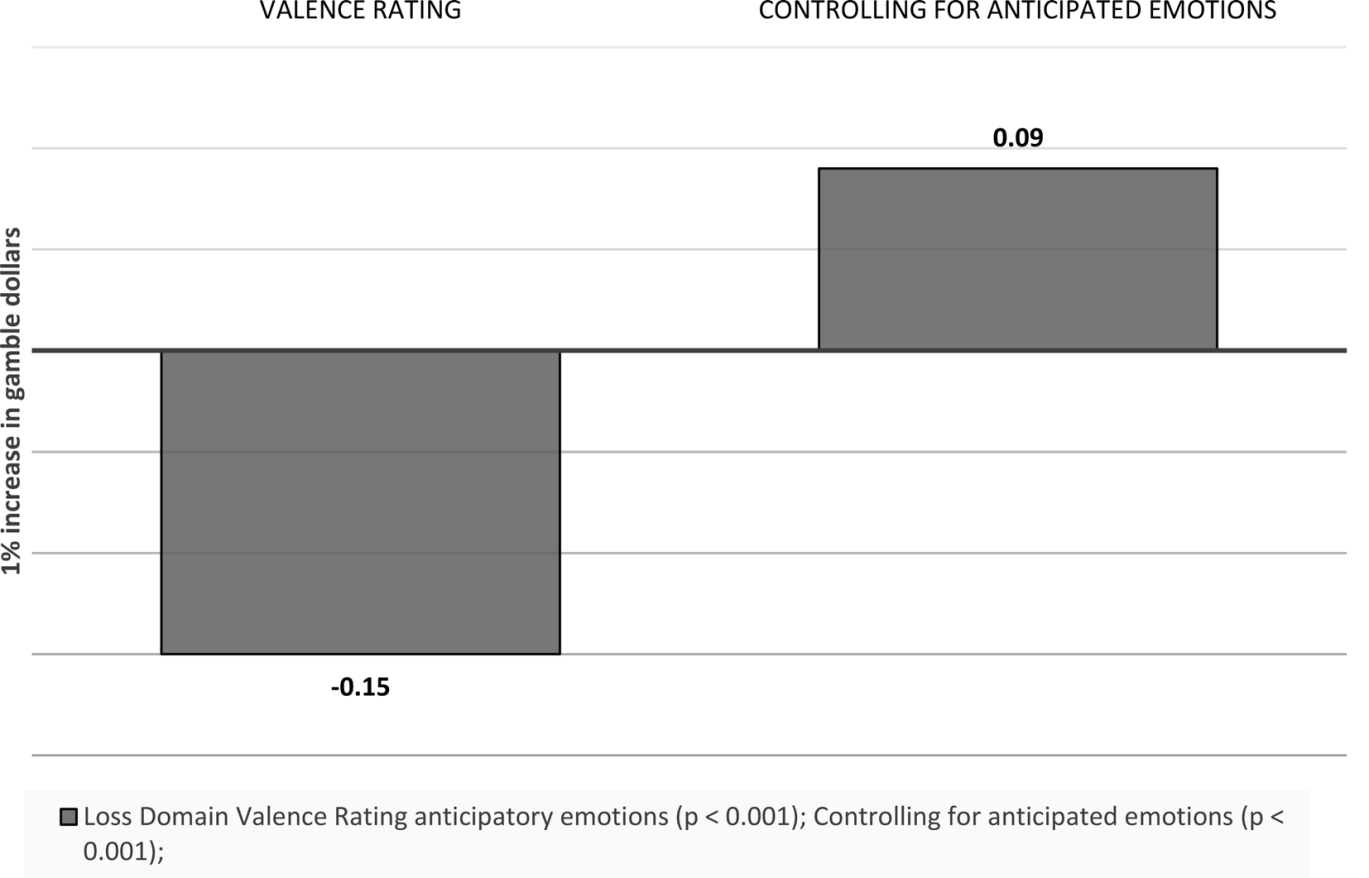
Visual depiction of the effects of outcome values on emotions

Gamble money. A one unit increase in gamble dollars (which equals a $10 change) is associated with a 0.15 (*p* < 0.001) decrease in the valence ratings of anticipatory emotions towards the gamble option in the loss domain, and controlling for anticipated emotions, the effect becomes 0.09 (*p* < 0.001). There was no statistically significant effect in the gain domain (see Fig. 14).

##### Anticipated emotions

Sure money. A 1% increase in sure dollars is associated with a 0.30 decrease in the valence ratings of anticipated emotions towards the sure option in the loss domain (*p* < 0.001), and a 0.31 increase in the gain domain (*p* < 0.001). Controlling for anticipatory emotions, the effect becomes 0.11 (*p* < 0.001) and 0.11 (*p* < 0.001) respectively (around two thirds smaller in each case).

**Fig. 15:**
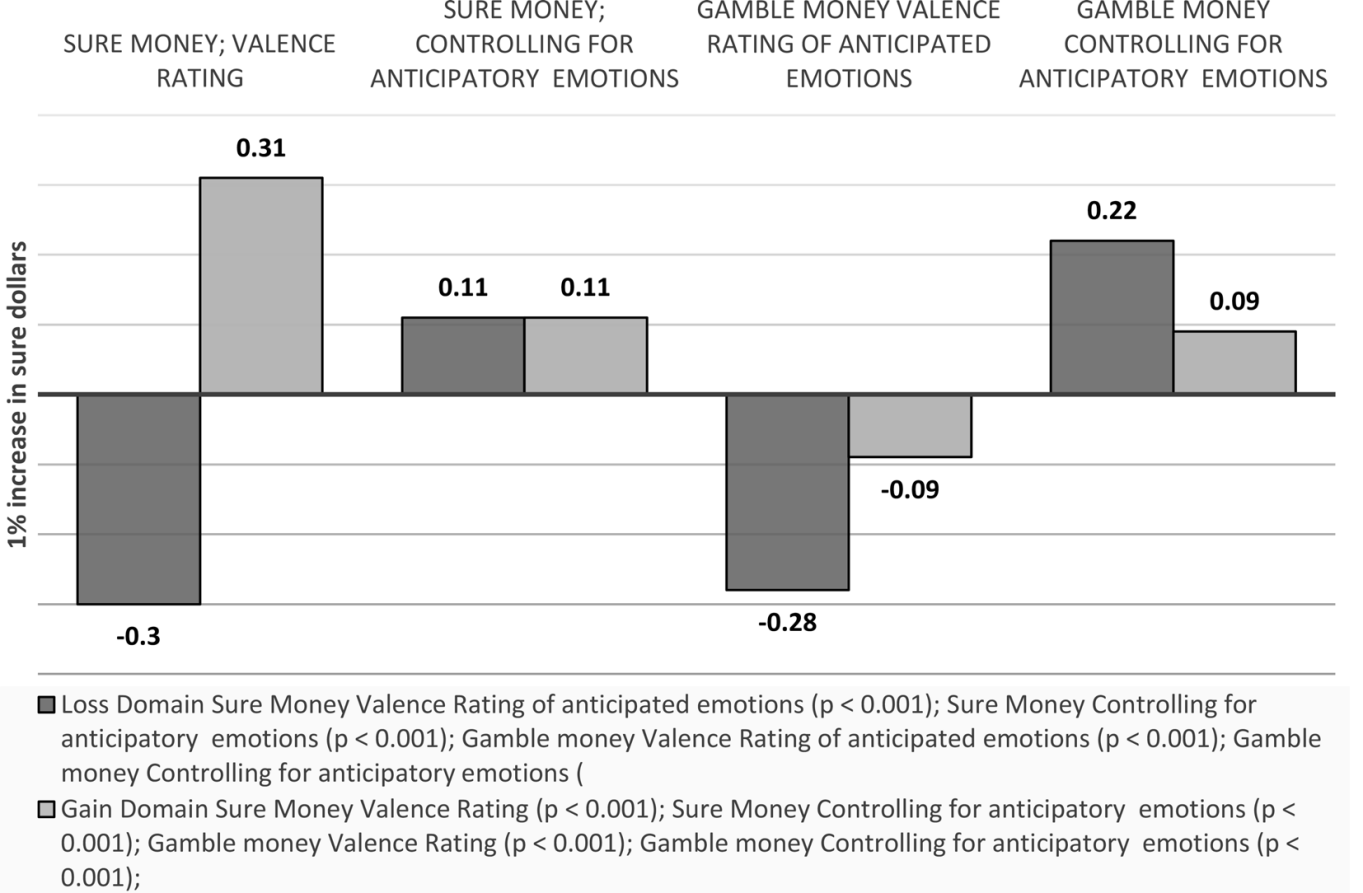
Visual depiction of the effects of outcome values on emotions

Gamble money. A one unit increase in gamble dollars (which equals a $10 change) is associated with a 0.28 (*p* < 0.001) decrease in the valence ratings of anticipated emotions towards the gamble option in the loss domain, and a 0.09 (*p* < 0.001) decrease in the gain domain. Controlling for anticipatory emotions, the effect becomes 0.22 in the loss domain (*p* < 0.001) and remains 0.09 (*p* < 0.001) in the gain domain (see Fig. 15).

#### Emotions as a mediator of outcome values’ effects on choice

**Fig. 16:**
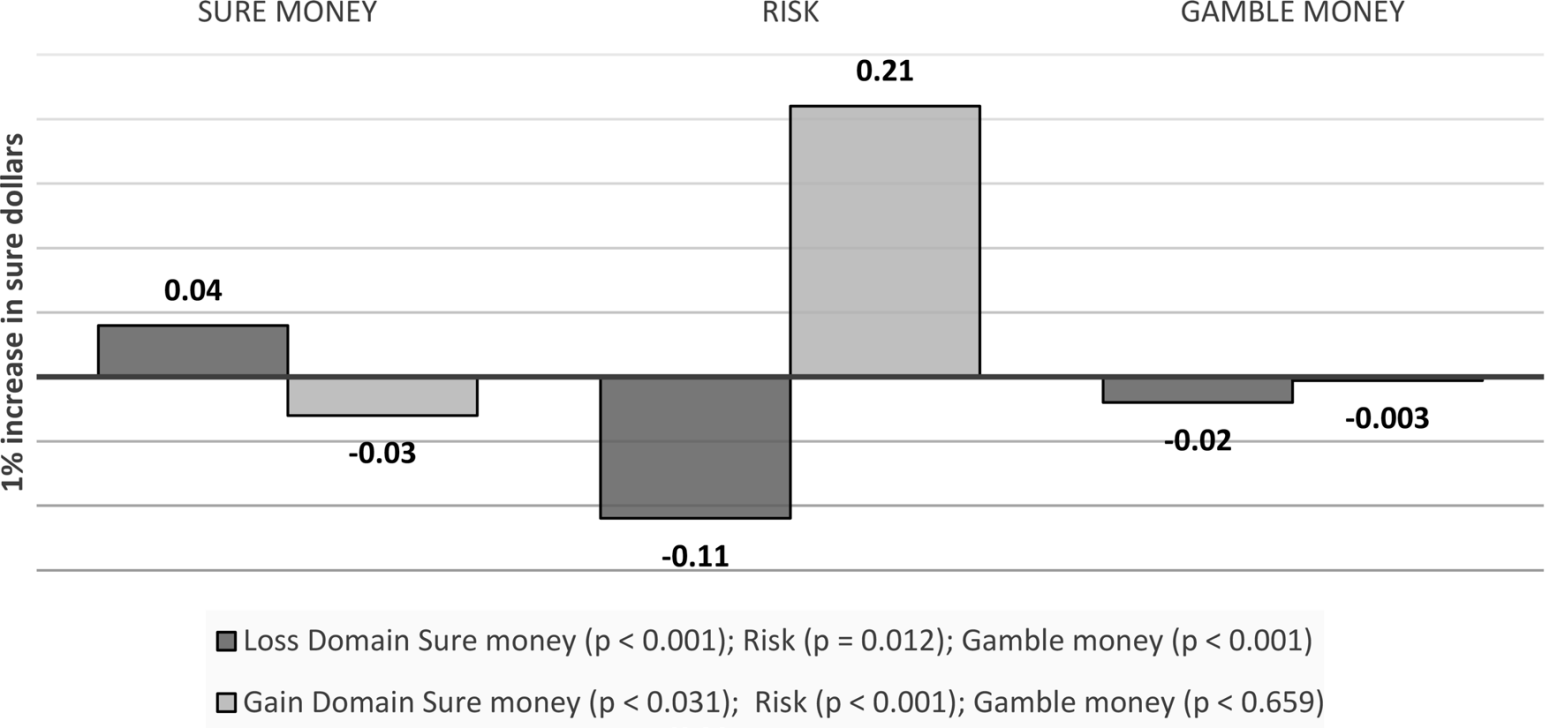
Visual depiction of the effects emotions as a mediator of outcome values effects on choice

Initial analyses confirmed that in the gain domain, (log) sure dollars (b = -0.03, *p* = 0.031) and risk (b = 0.21, *p* < 0.001) were significant predictors of risky choice. However, gamble dollars (b = -0.003, *p* = 0.659) were not significant predictors of risky choice. In the loss domain, (log) sure dollars (b = 0.04, *p* < 0.001), gamble dollars (b = -0.02, *p* < 0.001) and risk (b = -0.11, *p* = 0.012) were all significant predictors of risky choice (see Fig. 16).

##### Anticipatory emotions

Sure money. In the gain domain, anticipatory emotions towards the sure option mediated the effects of sure dollars on choice (indirect effect: -0.02, *p* < 0.001). In the loss domain, anticipatory emotions towards the sure option mediated the effects of sure dollars on choice (indirect effect: 0.01, *p* < 0.001).

**Fig. 17:**
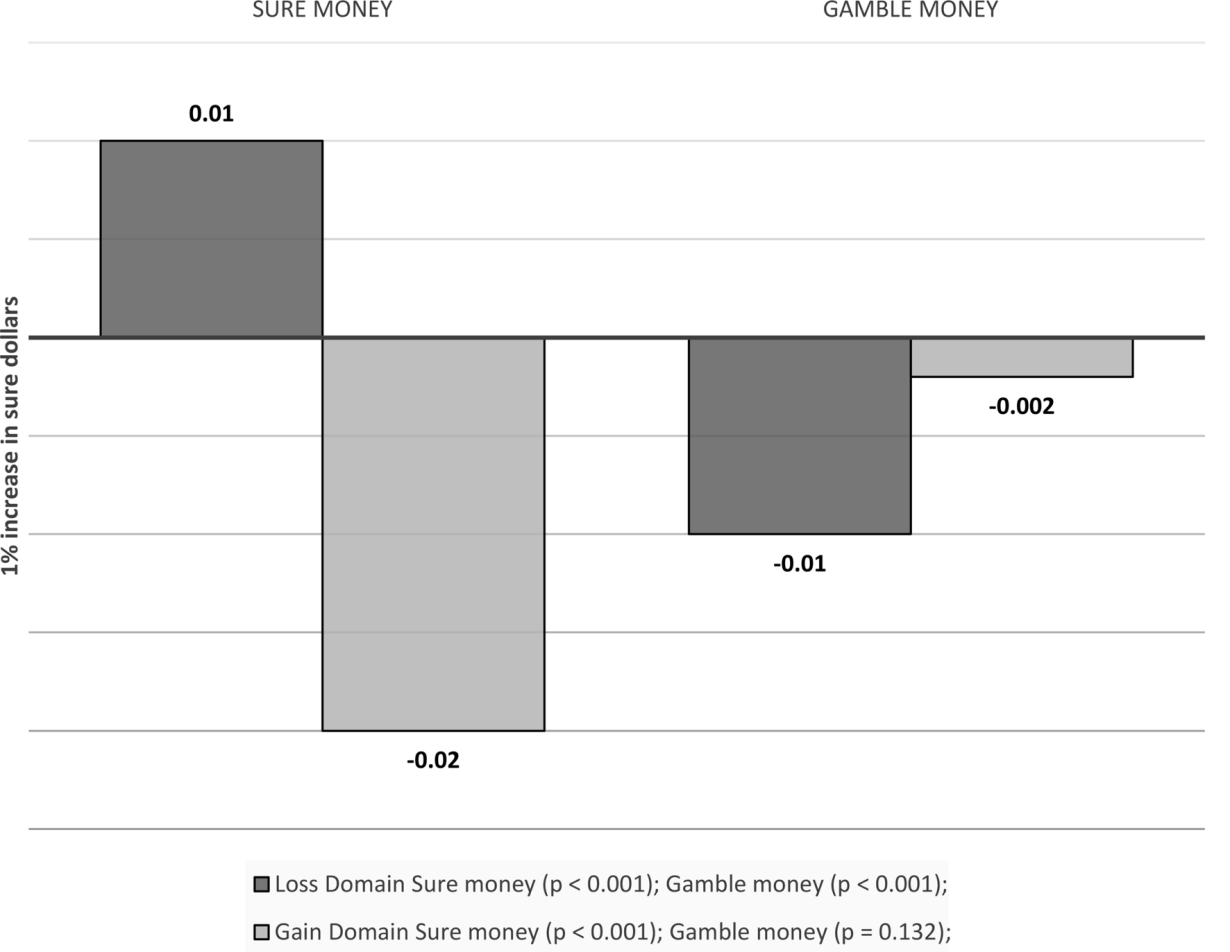
Visual depiction of the effects emotions as a mediator of outcome values effects on choice

Gamble money. In the gain domain, anticipatory emotions towards the gamble option didn’t mediate the effects of gamble dollars on choice (Indirect effect: -0.002, *p* = 0.132). In the loss domain, anticipatory emotions towards the gamble option mediated the effects of gamble dollars on choice (indirect effect: -0.01, *p* < 0.001) (see Fig. 17).

##### Anticipated emotions

**Fig. 18:**
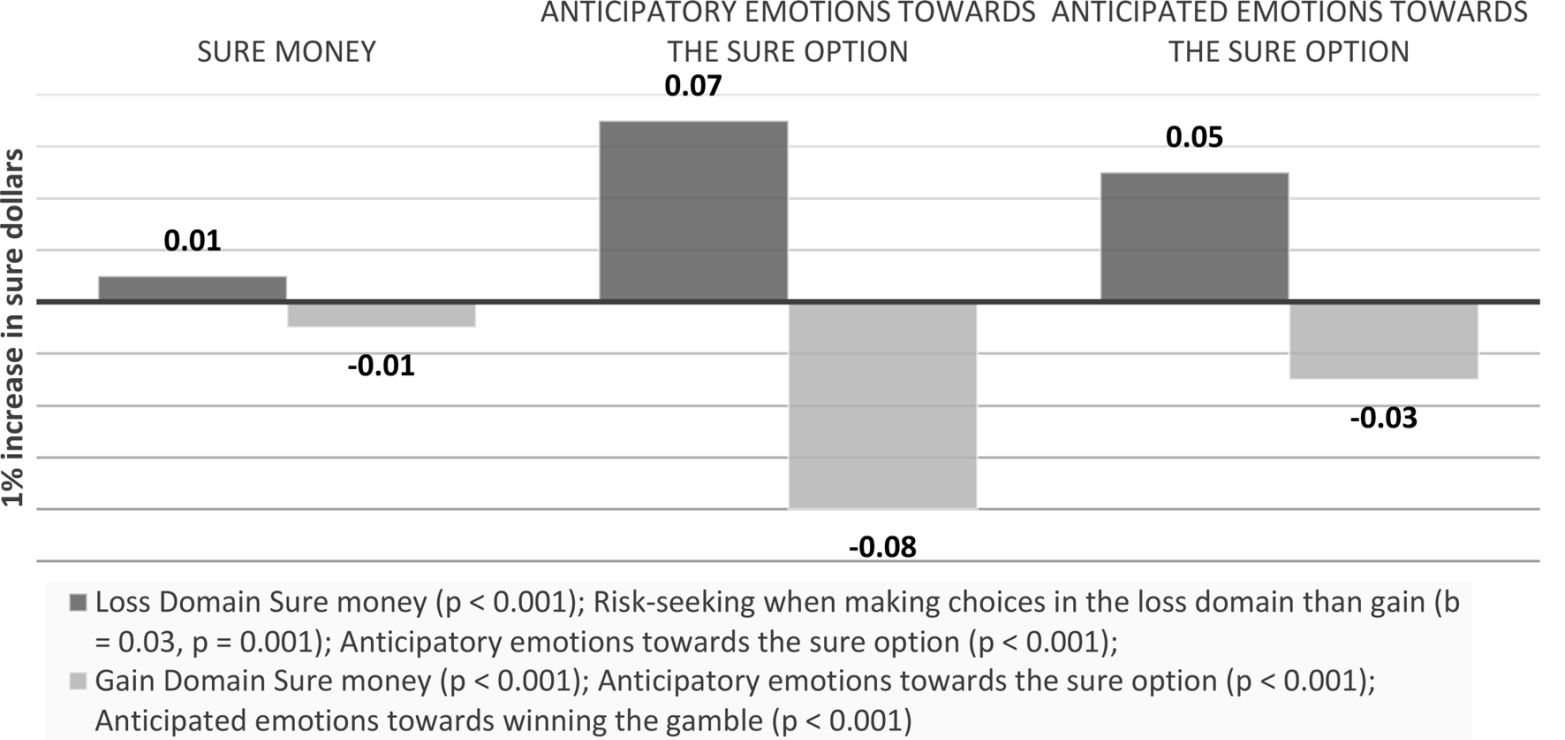
Visual depiction of the effects emotions as a mediator of outcome values effects on choice

Sure money. In the gain domain, anticipated emotions towards the sure also mediated the effects of gamble dollars on choice (indirect effect: -0.01, *p* < 0.001). In the loss domain, anticipated emotions towards the sure also mediated the effects of gamble dollars on choice (indirect effect: 0.01, *p* < 0.001) (see Fig. 18).

Gamble money. In the gain domain, anticipated emotions towards winning the gamble didn’t significantly mediate the effects of gamble dollars on choice (indirect effect: 0.00, *p* = 0.959), and anticipated emotions towards losing the gamble did not mediate the effects of gamble dollars on choice (indirect effect: -0.0007, *p* = 0.064). In loss, anticipated emotions towards winning the gamble (and losing the prospect amount) mediated the effects of gamble dollars on choice (indirect effect: -0.004, *p* = 0.006), and anticipated emotions towards losing the gamble (and not losing the prospect amount) was not a significant mediator (indirect effect: 0.0007, *p* = 0.980).

#### Emotions as a mediator of gain and loss domain effect on choice

We found that the same individual is more risk-seeking when making choices in the loss domain than gain (b = 0.03, *p* = 0.001). The loss condition increases risk-seeking indirectly via anticipatory emotions towards the sure option (indirect effect: 0.07, *p* < 0.001) as well as anticipated emotions towards the sure option (indirect effect: 0.05, *p* < 0.001).

**Fig. 19:**
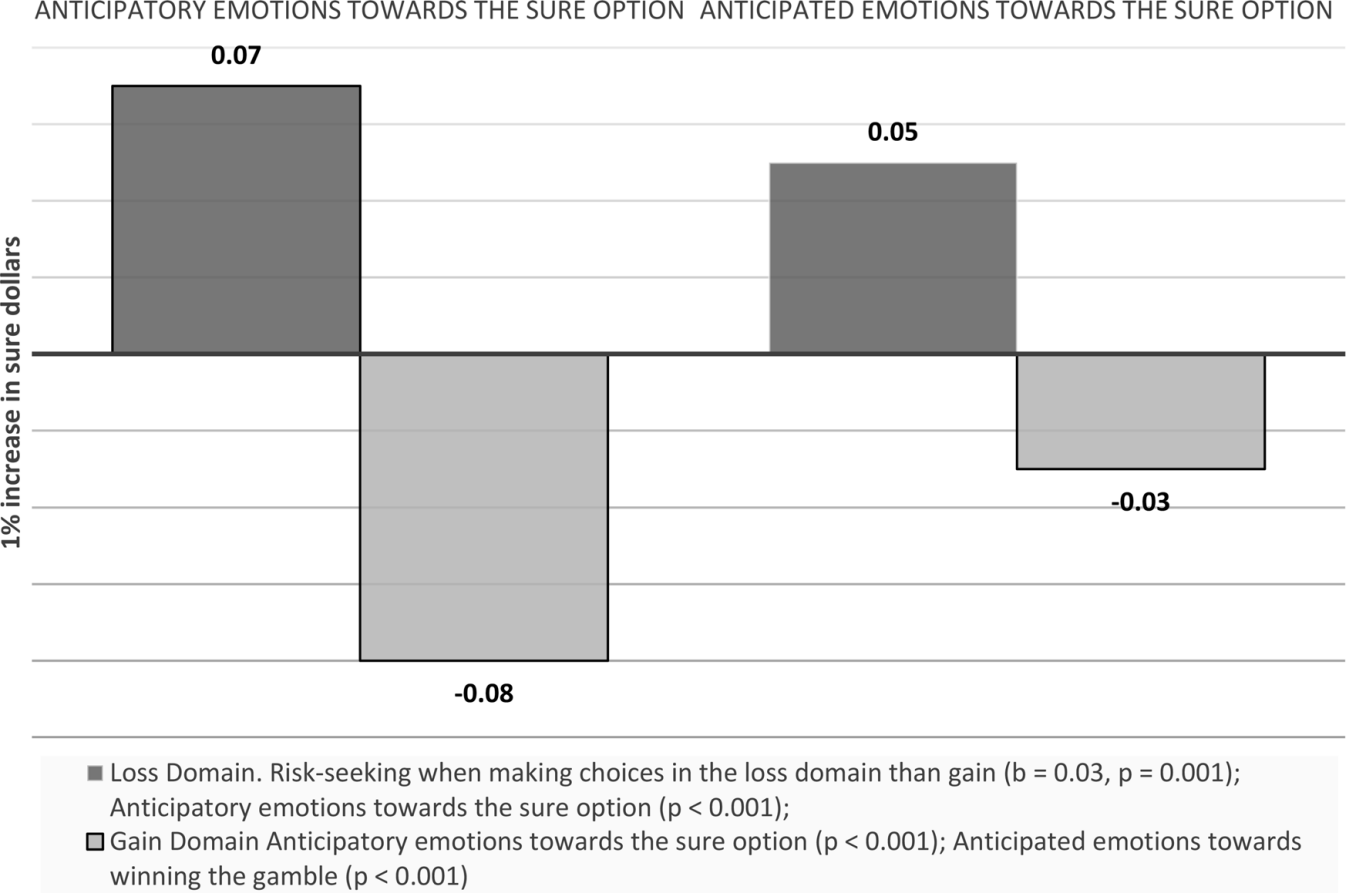
Visual depiction of the effects emotions as a mediator of outcome values effects on choice

Emotions towards the gamble, however, yielded an opposite influence on risk-taking. Anticipatory emotions towards the gamble was lower in loss domain than gain, which in turn, decreased risk taking (indirect effect: -0.08, *p* < 0.001). The results were the same for anticipated emotions towards winning the gamble (and receiving the prospect) (indirect effect: -0.03, *p* < 0.001). Anticipated emotions towards losing the gamble didn’t have significant mediation effects (indirect effect: 0.01, *p* = 0.147) (see Fig. 19).

## General discussion

### The effects of outcome values on emotions

Anticipatory emotions responded consistently to outcome values and the effects were in the expected direction. In general, higher values increased valence in gain domain and decreased valence in the loss domain. In Study 2 though, anticipatory emotions for the gamble in the loss domain didn’t change with value. For the gamble option, the effects were larger in the loss domain than gain in both studies. This finding suggests that anticipatory emotions respond more to risky losses (vs. gain): the fear from the possibility of losing loom greater than the excitement for the possibility of winning. For the sure option, however, the effects seemed larger in the gain domain than loss but only by a small margin in Study 2.

Anticipated emotions also increased with value in the gain domain, and decreased with value in the loss domain. We don’t observe any domain-dependent differences in anticipated emotions for the sure option in either studies. For the gamble, on the other hand, anticipated emotions changed more with value in the loss compared to gain, especially when the gambles were incentivized. This contradicts the findings in prior research where loss aversion was not reflected in anticipated emotion ratings but only in the way these emotions were weighed in decision-making (Charpentier, 2016; Charpentier et al., 2016). This could be due to methodological reasons. According to Charpentier et al. (2016), who studied the loss aversion, the payments were much lower than the current study. Our results are not consistent with Harinck et al. (2007) and Yechiam et al. (2014). These studies found a positivity bias for anticipated emotions, whereas our results are consistent with gain-loss neutrality. In our results, loss aversion also does not appear to be due to an asymmetry in feeling, inconsistent with other studies on high (though not small) hypothetical amounts (McGraw et al., 2010). Alternative explanations should be considered. As our results show an asymmetry between gains and losses for the risky but not safe choices, it is possible that risk and not loss aversion explain the results – and future research could seek to disentangle these.

Overall, anticipatory and anticipated emotions responded very similarly to changes in value for the sure gains in both studies (1 and 2). We can thus learn that, generally, the valence results for the non-incentivized amounts are likely to extend to incentive compatible scenarios. The responses were very similar for sure losses in the second study too, although anticipated emotions changed more with sure losses in the first non-incentivized study. It may thus be that that this result on anticipated emotions lacks ecological validity. In the incentivized study, anticipatory emotions didn’t respond to changes in the value of risky gains and responded to risky losses less strongly compared to anticipated emotions. Anticipated emotions, on the other hand, responded very consistently to the changes in the value of risky gains across the studies and models, and very strongly to changes in the value of risky losses in the incentivized study.

In Study 1, the findings were generally similar for intensity ratings. The only exception was that neither anticipatory emotions nor anticipated emotions for the sure option revealed a significant response to changes in value in the loss domain when they are measured as intensity. Due to resource constraints we did not look at intensity in the second study and future research could explore if similar findings hold for large, incentive compatible choices.

### Emotions as a mediator of outcome values’ effects on choice

The findings indicated that both anticipatory and anticipated emotions explained the effects of the value on choice for the sure gain and sure losses. The proportion mediated was larger for anticipatory emotions compared to anticipated emotions. Results were consistent across studies (1 and 2). For the risky gain, neither anticipatory nor anticipated emotions had a consistent mediating role in the effects of value on choice. For risky losses, anticipatory emotions were a consistent mediator, and anticipated emotions towards the prospect became a significant mediator only in incentivized study, although the proportion mediated was much smaller than anticipatory emotions.

### Emotions as a mediator of gain and loss domain effect on choice

The findings from both studies (1 and 2) indicated that all emotions towards the sure and the gamble option mediated the effect of framing on choice while anticipatory emotions mediated a larger portion of the effect. Previous research by Young et al. (2019) has found that only anticipatory emotions for the sure option explained the effects of framing on choice. We did find that anticipatory emotions towards the sure are indeed the strongest explanatory factor although anticipated emotions also show some mediation.

### Learning and implications

These findings have important implications for marketers and practitioners. Anticipated and anticipatory emotions may support marketers in their communication strategies, as they could increase the effectiveness of one-to-one communication, especially online, based on consumer personality variables, inferred from their online behaviour data. For example, marketers can use social networks likes or language to infer personality traits of agreeableness and conscientiousness, which are correlated to the self-control trait. In addition, in order to make better predictions, marketing researchers should consider a broad range of emotions likely to be salient at different points of the purchase and consumption process (Bee & Madrigal, 2013; Bettiga & Lamberti, 2020). It is important that marketers consider both how a consumer feels when they decide and how they expect to feel as a result of the decision, and how these interplay with each other and features of the decision such its importance and significance.

These findings can also be useful to other practitioners. For example, health interventions, especially those that target prevention behaviours, can include an emotional narrative that makes people believe that they will regret or worry eventually if they do not perform the suggested behaviours. People could be informed that how they feel when they decide affects their choice in addition to the expected outcomes of that choice and its importance and significance, which could alter the influence of emotions. When people are exposed to a risk, the psychological immune system will motivate people to cope with negative feelings and thoughts unconsciously. Since people are often unaware of their psychological defences systems, they tend to exaggerate their future emotional responses but not their current emotional and cognitive reactions (Xu & Guo, 2019).

To conclude, the study lays a critical foundation for understanding how emotions, especially the anticipation of losses, influence risky choices. By integrating these findings with real-world factors such as incentives and consequences, researchers and practitioners can develop targeted strategies to nudge behaviour and promote informed decision-making. For instance, health messages could be framed to emphasize potential benefits rather than risks, thereby encouraging positive behaviour change. Additionally, providing consumers with tools to identify and mitigate emotional biases in their decision-making processes can lead to more informed and rational choices.

### Conclusions and implications

Overall, anticipatory and anticipated emotions responded very similarly to changes in value for the sure gains in both studies (1 and 2). The findings also indicated that both anticipatory and anticipated emotions explained the effects of the value on choice for the sure gain and sure losses, while both mediated the effect of framing on choice towards the sure and the gamble option. Although anticipatory emotions mediated a larger portion of the effect, which was expected according to the existing literature, anticipated emotions also show some mediation. Marketers and other practitioners can use these results to understand and influence people’s choices.

## Electronic supplementary material

Below is the link to the electronic supplementary material.

Supplementary Material 1

## Data Availability

The data presented in this study are available upon reasonable request from the corresponding author.

## Footnotes

1 In general, effect sizes were small to moderate. For example, in this case, F(1,310) = 240.9, *p* < 0.001, r^2^ within = 0.18, r^2^ between = 0.007, overall = 0.10. Further details are available upon request.
