# Correction: How do emotions respond to outcome values and influence choice?

**DOI:** 10.1007/s00426-024-02027-7

**Published:** 2024-10-14

**Authors:** Aikaterini Grimani, Ayse Yemiscigil, Qing Wang, Georgi Kirilov, Laura Kudrna, Ivo Vlaev

**Affiliations:** 1 Warwick Business School, University of Warwick, Scarman Rd, Coventry, CV4 7AL UK; 2 Human Flourishing Program, Harvard University, Boston, USA; 3 Sofia University, Sofia, Bulgaria; 4 University of Birmingham, Birmingham, UK

**Correction: Psychological Research (2024)**

10.1007/s00426-024-02001-3

The funding information section was missing from this article and should have read.

