# Supplementary material for: How do emotions respond to outcome values and influence choice?
Source: Psychol Res. 2024 Jul 10;88(8):2234–50. doi: 10.1007/s00426-024-02001-3 (PMC11522079; doi:10.1007/s00426-024-02001-3)
Supplement: Supplementary file 1 — Supplementary Material 1 [file 426_2024_2001_MOESM1_ESM.docx]

**Supplementary Material**

Below are the lotteries provided to participants for the scenarios (1) anticipated negative (2) anticipated positive (3) anticipatory negative and (4) anticipatory positive.

1. *Lotteries, anticipated negative*

SCENARIO: GAMBLE WITH LOSS
Imagine choosing between "losing $30 for certain" or a "50% chance of losing $100" (and hence there is a 50% chance of not losing anything). Which option would you choose?  Here you would have to imagine making choices between playing a gamble that can make you lose an amount of money and losing a smaller amount for sure. Each pair of options is again presented as two pie charts. Imagine that a spinner is attached to the center of the pie chart and after the spinner is spun you will lose the money written in the region where and the pointer lands. As you can see, the pie charts representing ‘Sure Loss’ will always make you lose a certain amount while the pie charts representing ‘Loss Gamble’ can make you lose either a bigger amount or zero (the two regions of these pie charts represent the probabilities for each loss respectively).

Q134 Imagine you have chosen "sure loss" for  gambling task 1 and lost $16. Rate the valence of your feelings (how positive or negative you feel) on the below scale:

- 1 (Strongly Negative) (1)
- 2 (2)
- 3 (3)
- 4 (Neutral) (4)
- 5 (5)
- 6 (6)
- 7 (Strongly Positive) (7)

Q136 Imagine you have chosen "sure loss" for gambling task 1 and lost $16. Rate the intensity of your feelings on the below scale:

- 1 (Very Low) (1)
- 2 (2)
- 3 (3)
- 4 (4)
- 5 (5)
- 6 (6)
- 7 (Very High) (7)

Q138 Imagine you have already played gambling task 1 and lost $0. Rate the valence of your feelings (how positive or negative you feel) on the below scale:

- 1 (Strongly Negative) (1)
- 2 (2)
- 3 (3)
- 4 (Neutral) (4)
- 5 (5)
- 6 (6)
- 7 (Strongly Positive) (7)

Q140 Imagine you have already played gambling task 1 and lost $0. Rate the intensity of your feelings on the below scale:

- 1 (Very Low) (1)
- 2 (2)
- 3 (3)
- 4 (4)
- 5 (5)
- 0 (6)
- 7 (Very High) (7)

Q142 Imagine you have already played gambling task 1 and lost $100. Rate the valence of your feelings (how positive or negative you feel) on the below scale:

- 1 (Strongly Negative) (1)
- 2 (2)
- 3 (3)
- 4 (Neutral) (4)
- 5 (5)
- 6 (6)
- 7 (Strongly Positive) (7)

Q144 Imagine you have already played gambling task 1 and lost $100. Rate the intensity of your feelings on the below scale:

- 1(Very Low) (1)
- 2 (2)
- 3 (3)
- 4 (4)
- 5 (5)
- 6 (6)
- 7 (Very High) (7)

Q146 Gambling Task 1: Imagine choosing between "losing $30 for certain" or a "50% chance of losing $100" (and hence there is a 50% chance of not losing anything). Which option would you choose?  Here you would have to imagine making choices between playing a gamble that can make you lose an amount of money and losing a smaller amount for sure. Each pair of options is again presented as two pie charts. Imagine that a spinner is attached to the center of the pie chart and after the spinner is spun you will lose the money written in the region where and the pointer lands. As you can see, the pie charts representing ‘Sure Loss’ will always make you lose a certain amount while the pie charts representing ‘Loss Gamble’ can make you lose either a bigger amount or zero (the two regions of these pie charts represent the probabilities for each loss respectively). Please circle the pie chart you would prefer (sure loss or gamble) in each pair. Note that there are no correct answers and your choice is a matter of personal preference, but try to choose which option (sure loss or gamble) you would prefer if this choice was made for real.

- A. Sure Loss (1)
- B. Gamble (2)

End of Block: Gamble With Loss - Task 1

Start of Block: Gamble With Loss - Task 2

Q148 Imagine you have chosen "sure loss" for  gambling task 2 and lost $46. Rate the valence of your feelings (how positive or negative you feel) on the below scale:

- 1 (Strongly Negative) (1)
- 2 (2)
- 3 (3)
- 4 (Neutral) (4)
- 5 (5)
- 6 (6)
- 7 (Strongly Positive) (7)

Q150 Imagine you have chosen "sure loss" for  gambling task 2 and lost $46. Rate the intensity of your feelings on the below scale:

- 1 (Very Low) (1)
- 2 (2)
- 3 (3)
- 4 (4)
- 5 (5)
- 6 (6)
- 7 (Very High) (7)

Q152 Imagine you have already played gambling task 2 and lost $0. Rate the valence of your feelings (how positive or negative you feel) on the below scale:

- 1 (Strongly Negative) (1)
- 2 (2)
- 3 (3)
- 4 (Neutral) (4)
- 5 (5)
- 6 (6)
- 7 (Strongly Positive) (7)

Q154 Imagine you have already played gambling task 2 and lost $0. Rate the intensity of your feelings on the below scale:

- 1 (Very Low) (1)
- 2 (2)
- 3 (3)
- 4 (4)
- 5 (5)
- 0 (6)
- 7 (Very High) (7)

Q156 Imagine you have already played gambling task 2 and lost $100. Rate the valence of your feelings (how positive or negative you feel) on the below scale:

- 1 (Strongly Negative) (1)
- 2 (2)
- 3 (3)
- 4 (Neutral) (4)
- 5 (5)
- 6 (6)
- 7 (Strongly Positive) (7)

Q158 Imagine you have already played gambling task 2 and lost $100. Rate the intensity of your feelings on the below scale:

- 1(Very Low) (1)
- 2 (2)
- 3 (3)
- 4 (4)
- 5 (5)
- 6 (6)
- 7 (Very High) (7)

Q160 Gambling Task 2:  Imagine choosing between "losing $30 for certain" or a "50% chance of losing $100" (and hence there is a 50% chance of not losing anything). Which option would you choose?  Here you would have to imagine making choices between playing a gamble that can make you lose an amount of money and losing a smaller amount for sure. Each pair of options is again presented as two pie charts. Imagine that a spinner is attached to the center of the pie chart and after the spinner is spun you will lose the money written in the region where and the pointer lands. As you can see, the pie charts representing ‘Sure Loss’ will always make you lose a certain amount while the pie charts representing ‘Loss Gamble’ can make you lose either a bigger amount or zero (the two regions of these pie charts represent the probabilities for each loss respectively). Please circle the pie chart you would prefer (sure loss or gamble) in each pair. Note that there are no correct answers and your choice is a matter of personal preference, but try to choose which option (sure loss or gamble) you would prefer if this choice was made for real. 

- A. Sure Loss (1)
- B. Gamble (2)

End of Block: Gamble With Loss - Task 2

Start of Block: Gamble With Loss - Task 3

Q162 Imagine you have chosen "sure loss" for  gambling task 3 and lost $76. Rate the valence of your feelings (how positive or negative you feel) on the below scale:

- 1 (Strongly Negative) (1)
- 2 (2)
- 3 (3)
- 4 (Neutral) (4)
- 5 (5)
- 6 (6)
- 7 (Strongly Positive) (7)

Q164 Imagine you have chosen "sure loss" for  gambling task 3 and lost $76. Rate the intensity of your feelings on the below scale:

- 1 (Very Low) (1)
- 2 (2)
- 3 (3)
- 4 (4)
- 5 (5)
- 6 (6)
- 7 (Very High) (7)

Q166 Imagine you have already played gambling task 3 and lost $0. Rate the valence of your feelings (how positive or negative you feel) on the below scale:

- 1 (Strongly Negative) (1)
- 2 (2)
- 3 (3)
- 4 (Neutral) (4)
- 5 (5)
- 6 (6)
- 7 (Strongly Positive) (7)

Q168 Imagine you have already played gambling task 3 and lost $0. Rate the intensity of your feelings on the below scale:

- 1 (Very Low) (1)
- 2 (2)
- 3 (3)
- 4 (4)
- 5 (5)
- 0 (6)
- 7 (Very High) (7)

Q170 Imagine you have already played gambling task 3 and lost $100. Rate the valence of your feelings (how positive or negative you feel) on the below scale:

- 1 (Strongly Negative) (1)
- 2 (2)
- 3 (3)
- 4 (Neutral) (4)
- 5 (5)
- 6 (6)
- 7 (Strongly Positive) (7)

Q172 Imagine you have already played gambling task 3 and lost $100. Rate the intensity of your feelings on the below scale:

- 1(Very Low) (1)
- 2 (2)
- 3 (3)
- 4 (4)
- 5 (5)
- 6 (6)
- 7 (Very High) (7)

Q174 Gambling Task 3: Imagine choosing between "losing $30 for certain" or a "50% chance of losing $100" (and hence there is a 50% chance of not losing anything). Which option would you choose?  Here you would have to imagine making choices between playing a gamble that can make you lose an amount of money and losing a smaller amount for sure. Each pair of options is again presented as two pie charts. Imagine that a spinner is attached to the center of the pie chart and after the spinner is spun you will lose the money written in the region where and the pointer lands. As you can see, the pie charts representing ‘Sure Loss’ will always make you lose a certain amount while the pie charts representing ‘Loss Gamble’ can make you lose either a bigger amount or zero (the two regions of these pie charts represent the probabilities for each loss respectively). Please circle the pie chart you would prefer (sure loss or gamble) in each pair. Note that there are no correct answers and your choice is a matter of personal preference, but try to choose which option (sure loss or gamble) you would prefer if this choice was made for real. 

- A. Sure Loss (1)
- B. Gamble (2)

End of Block: Gamble With Loss - Task 3

Start of Block: Gamble With Loss - Task 4

Q190 Imagine you have chosen "sure loss" for  gambling task 4 and lost  $15. Rate the valence of your feelings (how positive or negative you feel) on the below scale:

- 1 (Strongly Negative) (1)
- 2 (2)
- 3 (3)
- 4 (Neutral) (4)
- 5 (5)
- 6 (6)
- 7 (Strongly Positive) (7)

Q192 Imagine you have chosen "sure loss" for  gambling task 4 and lost  $15. Rate the intensity of your feelings on the below scale:

- 1 (Very Low) (1)
- 2 (2)
- 3 (3)
- 4 (4)
- 5 (5)
- 6 (6)
- 7 (Very High) (7)

Q194 Imagine you have already played gambling task 4 and lost $0. Rate the intensity of your feelings on the below scale:

- 1 (Strongly Negative) (1)
- 2 (2)
- 3 (3)
- 4 (Neutral) (4)
- 5 (5)
- 6 (6)
- 7 (Strongly Positive) (7)

Q196 :Imagine you have already played gambling task 4 and lost $0. Rate the valence of your feelings (how positive or negative you feel) on the below scale:

- 1 (Very Low) (1)
- 2 (2)
- 3 (3)
- 4 (4)
- 5 (5)
- 0 (6)
- 7 (Very High) (7)

Q244 Imagine you have already played gambling task 4 and lost $200. Rate the valence of your feelings (how positive or negative you feel) on the below scale: on text

- 1 (Strongly Negative) (1)
- 2 (2)
- 3 (3)
- 4 (Neutral) (4)
- 5 (5)
- 6 (6)
- 7 (Strongly Positive) (7)

Q200 Imagine you have already played gambling task 4 and lost $200. Rate the intensity of your feelings on the below scale:

- 1(Very Low) (1)
- 2 (2)
- 3 (3)
- 4 (4)
- 5 (5)
- 6 (6)
- 7 (Very High) (7)

Q202 Gambling Task 4:  Imagine choosing between "losing $30 for certain" or a "50% chance of losing $100" (and hence there is a 50% chance of not losing anything). Which option would you choose?  Here you would have to imagine making choices between playing a gamble that can make you lose an amount of money and losing a smaller amount for sure. Each pair of options is again presented as two pie charts. Imagine that a spinner is attached to the center of the pie chart and after the spinner is spun you will lose the money written in the region where and the pointer lands. As you can see, the pie charts representing ‘Sure Loss’ will always make you lose a certain amount while the pie charts representing ‘Loss Gamble’ can make you lose either a bigger amount or zero (the two regions of these pie charts represent the probabilities for each loss respectively). Please circle the pie chart you would prefer (sure loss or gamble) in each pair. Note that there are no correct answers and your choice is a matter of personal preference, but try to choose which option (sure loss or gamble) you would prefer if this choice was made for real. 

- A. Sure Loss (1)
- B. Gamble (2)

End of Block: Gamble With Loss - Task 4

Start of Block: Gamble With Loss - Task 5

Q204 Imagine you have chosen "sure loss" for  gambling task 5 and lost  $72. Rate the valence of your feelings (how positive or negative you feel) on the below scale:

- 1 (Strongly Negative) (1)
- 2 (2)
- 3 (3)
- 4 (Neutral) (4)
- 5 (5)
- 6 (6)
- 7 (Strongly Positive) (7)

Q206 Imagine you have chosen "sure loss" for  gambling task 5 and lost  $72. Rate the intensity of your feelings on the below scale:

- 1 (Very Low) (1)
- 2 (2)
- 3 (3)
- 4 (4)
- 5 (5)
- 6 (6)
- 7 (Very High) (7)

Q208 Imagine you have already played gambling task 5 and lost $0. Rate the valence of your feelings (how positive or negative you feel) on the below scale:

- 1 (Strongly Negative) (1)
- 2 (2)
- 3 (3)
- 4 (Neutral) (4)
- 5 (5)
- 6 (6)
- 7 (Strongly Positive) (7)

Q210 Imagine you have already played gambling task 5 and lost $0. Rate the intensity of your feelings on the below scale:

- 1 (Very Low) (1)
- 2 (2)
- 3 (3)
- 4 (4)
- 5 (5)
- 0 (6)
- 7 (Very High) (7)

Q212 Imagine you have already played gambling task 5 and lost $200. Rate the valence of your feelings (how positive or negative you feel) on the below scale:

- 1 (Strongly Negative) (1)
- 2 (2)
- 3 (3)
- 4 (Neutral) (4)
- 5 (5)
- 6 (6)
- 7 (Strongly Positive) (7)

Q214 Imagine you have already played gambling task 5 and lost $200. Rate the intensity of your feelings on the below scale:

- 1(Very Low) (1)
- 2 (2)
- 3 (3)
- 4 (4)
- 5 (5)
- 6 (6)
- 7 (Very High) (7)

Q216 Gambling Task 5: Imagine choosing between "losing $30 for certain" or a "50% chance of losing $100" (and hence there is a 50% chance of not losing anything). Which option would you choose?  Here you would have to imagine making choices between playing a gamble that can make you lose an amount of money and losing a smaller amount for sure. Each pair of options is again presented as two pie charts. Imagine that a spinner is attached to the center of the pie chart and after the spinner is spun you will lose the money written in the region where and the pointer lands. As you can see, the pie charts representing ‘Sure Loss’ will always make you lose a certain amount while the pie charts representing ‘Loss Gamble’ can make you lose either a bigger amount or zero (the two regions of these pie charts represent the probabilities for each loss respectively). Please circle the pie chart you would prefer (sure loss or gamble) in each pair. Note that there are no correct answers and your choice is a matter of personal preference, but try to choose which option (sure loss or gamble) you would prefer if this choice was made for real. 

- A. Sure Loss (1)
- B. Gamble (2)

End of Block: Gamble With Loss - Task 5

Start of Block: Gamble With Loss - Task 6

Q232 Imagine you have chosen "sure loss" for  gambling task 6 and lost  $142. Rate the valence of your feelings (how positive or negative you feel) on the below scale:

- 1 (Strongly Negative) (1)
- 2 (2)
- 3 (3)
- 4 (Neutral) (4)
- 5 (5)
- 6 (6)
- 7 (Strongly Positive) (7)

Q234 Imagine you have chosen "sure loss" for  gambling task 6 and lost  $142. Rate the intensity of your feelings on the below scale:

- 1 (Very Low) (1)
- 2 (2)
- 3 (3)
- 4 (4)
- 5 (5)
- 6 (6)
- 7 (Very High) (7)

Q236 Imagine you have already played gambling task 6 and lost $0. Rate the valence of your feelings (how positive or negative you feel) on the below scale:

- 1 (Strongly Negative) (1)
- 2 (2)
- 3 (3)
- 4 (Neutral) (4)
- 5 (5)
- 6 (6)
- 7 (Strongly Positive) (7)

Q238 Imagine you have already played gambling task 6 and lost $0. Rate the intensity of your feelings on the below scale:

- 1 (Very Low) (1)
- 2 (2)
- 3 (3)
- 4 (4)
- 5 (5)
- 0 (6)
- 7 (Very High) (7)

Q240 Imagine you have already played gambling task 6 and lost $200. Rate the valence of your feelings (how positive or negative you feel) on the below scale:

- 1 (Strongly Negative) (1)
- 2 (2)
- 3 (3)
- 4 (Neutral) (4)
- 5 (5)
- 6 (6)
- 7 (Strongly Positive) (7)

Q242 Imagine you have already played gambling task 6 and lost $200. Rate the intensity of your feelings on the below scale:

- 1(Very Low) (1)
- 2 (2)
- 3 (3)
- 4 (4)
- 5 (5)
- 6 (6)
- 7 (Very High) (7)

Q244 Gambling Task 6: Imagine choosing between "losing $30 for certain" or a "50% chance of losing $100" (and hence there is a 50% chance of not losing anything). Which option would you choose?  Here you would have to imagine making choices between playing a gamble that can make you lose an amount of money and losing a smaller amount for sure. Each pair of options is again presented as two pie charts. Imagine that a spinner is attached to the center of the pie chart and after the spinner is spun you will lose the money written in the region where and the pointer lands. As you can see, the pie charts representing ‘Sure Loss’ will always make you lose a certain amount while the pie charts representing ‘Loss Gamble’ can make you lose either a bigger amount or zero (the two regions of these pie charts represent the probabilities for each loss respectively). Please circle the pie chart you would prefer (sure loss or gamble) in each pair. Note that there are no correct answers and your choice is a matter of personal preference, but try to choose which option (sure loss or gamble) you would prefer if this choice was made for real. 

- A. Sure Loss (1)
- B. Gamble (2)

End of Block: Gamble With Loss - Task 6

Start of Block: Gamble With Loss - Task 7

Q246 Imagine you have chosen "sure loss" for  gambling task 7 and lost  $95. Rate the valence of your feelings (how positive or negative you feel) on the below scale:

- 1 (Strongly Negative) (1)
- 2 (2)
- 3 (3)
- 4 (Neutral) (4)
- 5 (5)
- 6 (6)
- 7 (Strongly Positive) (7)

Q248 Imagine you have chosen "sure loss" for  gambling task 7 and lost  $95. Imagine you have already played the gamble and won $100. Rate the intensity of your feelings on the below scale:

- 1 (Very Low) (1)
- 2 (2)
- 3 (3)
- 4 (4)
- 5 (5)
- 6 (6)
- 7 (Very High) (7)

Q250 Imagine you have already played gambling task 7 and lost $0. Rate the valence of your feelings (how positive or negative you feel) on the below scale:

- 1 (Strongly Negative) (1)
- 2 (2)
- 3 (3)
- 4 (Neutral) (4)
- 5 (5)
- 6 (6)
- 7 (Strongly Positive) (7)

Q252 Imagine you have already played gambling task 7 and lost $0. Rate the intensity of your feelings on the below scale:

- 1 (Very Low) (1)
- 2 (2)
- 3 (3)
- 4 (4)
- 5 (5)
- 0 (6)
- 7 (Very High) (7)

Q254 Imagine you have already played gambling task 7 and lost $300. Rate the valence of your feelings (how positive or negative you feel) on the below scale:

- 1 (Strongly Negative) (1)
- 2 (2)
- 3 (3)
- 4 (Neutral) (4)
- 5 (5)
- 6 (6)
- 7 (Strongly Positive) (7)

Q256 Imagine you have already played gambling task 7 and lost $300. Rate the intensity of your feelings on the below scale:

- 1(Very Low) (1)
- 2 (2)
- 3 (3)
- 4 (4)
- 5 (5)
- 6 (6)
- 7 (Very High) (7)

Q258 Gambling Task 7:  Imagine choosing between "losing $30 for certain" or a "50% chance of losing $100" (and hence there is a 50% chance of not losing anything). Which option would you choose?  Here you would have to imagine making choices between playing a gamble that can make you lose an amount of money and losing a smaller amount for sure. Each pair of options is again presented as two pie charts. Imagine that a spinner is attached to the center of the pie chart and after the spinner is spun you will lose the money written in the region where and the pointer lands. As you can see, the pie charts representing ‘Sure Loss’ will always make you lose a certain amount while the pie charts representing ‘Loss Gamble’ can make you lose either a bigger amount or zero (the two regions of these pie charts represent the probabilities for each loss respectively). Please circle the pie chart you would prefer (sure loss or gamble) in each pair. Note that there are no correct answers and your choice is a matter of personal preference, but try to choose which option (sure loss or gamble) you would prefer if this choice was made for real. 

- A. Sure Loss (1)
- B. Gamble (2)

End of Block: Gamble With Loss - Task 7

Start of Block: Gamble With Loss - Task 8

Q260 Imagine you have chosen "sure loss" for  gambling task 8 and lost  $70. Rate the valence of your feelings (how positive or negative you feel) on the below scale:

- 1 (Strongly Negative) (1)
- 2 (2)
- 3 (3)
- 4 (Neutral) (4)
- 5 (5)
- 6 (6)
- 7 (Strongly Positive) (7)

Q262 Imagine you have chosen "sure loss" for  gambling task 8 and lost  $70. Rate the intensity of your feelings on the below scale:

- 1 (Very Low) (1)
- 2 (2)
- 3 (3)
- 4 (4)
- 5 (5)
- 6 (6)
- 7 (Very High) (7)

Q264 Imagine you have already played gambling task 8 and lost $0. Rate the valence of your feelings (how positive or negative you feel) on the below scale:

- 1 (Strongly Negative) (1)
- 2 (2)
- 3 (3)
- 4 (Neutral) (4)
- 5 (5)
- 6 (6)
- 7 (Strongly Positive) (7)

Q266 Imagine you have already played gambling task 8 and lost $0. Rate the intensity of your feelings on the below scale:

- 1 (Very Low) (1)
- 2 (2)
- 3 (3)
- 4 (4)
- 5 (5)
- 0 (6)
- 7 (Very High) (7)

Q268 Imagine you have already played gambling task 8 and lost  $300.  Rate the valence of your feelings (how positive or negative you feel) on the below scale:

- 1 (Strongly Negative) (1)
- 2 (2)
- 3 (3)
- 4 (Neutral) (4)
- 5 (5)
- 6 (6)
- 7 (Strongly Positive) (7)

Q270 Imagine you have already played gambling task 8 and lost  $300. Rate the intensity of your feelings on the below scale:

- 1(Very Low) (1)
- 2 (2)
- 3 (3)
- 4 (4)
- 5 (5)
- 6 (6)
- 7 (Very High) (7)

Q272 Gambling Task 8: Imagine choosing between "losing $30 for certain" or a "50% chance of losing $100" (and hence there is a 50% chance of not losing anything). Which option would you choose?  Here you would have to imagine making choices between playing a gamble that can make you lose an amount of money and losing a smaller amount for sure. Each pair of options is again presented as two pie charts. Imagine that a spinner is attached to the center of the pie chart and after the spinner is spun you will lose the money written in the region where and the pointer lands. As you can see, the pie charts representing ‘Sure Loss’ will always make you lose a certain amount while the pie charts representing ‘Loss Gamble’ can make you lose either a bigger amount or zero (the two regions of these pie charts represent the probabilities for each loss respectively). Please circle the pie chart you would prefer (sure loss or gamble) in each pair. Note that there are no correct answers and your choice is a matter of personal preference, but try to choose which option (sure loss or gamble) you would prefer if this choice was made for real. 

- A. Sure Loss (1)
- B. Gamble (2)

End of Block: Gamble With Loss - Task 8

Start of Block: Gamble With Loss - Task 9

Q274 Imagine you have chosen "sure loss" for  gambling task 9 and lost  $192. Rate the valence of your feelings (how positive or negative you feel) on the below scale:

- 1 (Strongly Negative) (1)
- 2 (2)
- 3 (3)
- 4 (Neutral) (4)
- 5 (5)
- 6 (6)
- 7 (Strongly Positive) (7)

Q276 Imagine you have chosen "sure loss" for  gambling task 9 and lost  $192. Rate the intensity of your feelings on the below scale:

- 1 (Very Low) (1)
- 2 (2)
- 3 (3)
- 4 (4)
- 5 (5)
- 6 (6)
- 7 (Very High) (7)

Q278 Imagine you have already played gambling task 9 and lost $0. Rate the valence of your feelings (how positive or negative you feel) on the below scale:

- 1 (Strongly Negative) (1)
- 2 (2)
- 3 (3)
- 4 (Neutral) (4)
- 5 (5)
- 6 (6)
- 7 (Strongly Positive) (7)

Q280 Imagine you have already played gambling task 9 and lost $0. Rate the intensity of your feelings on the below scale:

- 1 (Very Low) (1)
- 2 (2)
- 3 (3)
- 4 (4)
- 5 (5)
- 0 (6)
- 7 (Very High) (7)

Q282 Imagine you have already played gambling task 9 and lost  $300. Rate the valence of your feelings (how positive or negative you feel) on the below scale:

- 1 (Strongly Negative) (1)
- 2 (2)
- 3 (3)
- 4 (Neutral) (4)
- 5 (5)
- 6 (6)
- 7 (Strongly Positive) (7)

Q284 Imagine you have already played gambling task 9 and lost  $300. Rate the intensity of your feelings on the below scale:

- 1(Very Low) (1)
- 2 (2)
- 3 (3)
- 4 (4)
- 5 (5)
- 6 (6)
- 7 (Very High) (7)

Q286 Gambling Task 9: Imagine choosing between "losing $30 for certain" or a "50% chance of losing $100" (and hence there is a 50% chance of not losing anything). Which option would you choose?  Here you would have to imagine making choices between playing a gamble that can make you lose an amount of money and losing a smaller amount for sure. Each pair of options is again presented as two pie charts. Imagine that a spinner is attached to the center of the pie chart and after the spinner is spun you will lose the money written in the region where and the pointer lands. As you can see, the pie charts representing ‘Sure Loss’ will always make you lose a certain amount while the pie charts representing ‘Loss Gamble’ can make you lose either a bigger amount or zero (the two regions of these pie charts represent the probabilities for each loss respectively). Please circle the pie chart you would prefer (sure loss or gamble) in each pair. Note that there are no correct answers and your choice is a matter of personal preference, but try to choose which option (sure loss or gamble) you would prefer if this choice was made for real. 

- A. Sure Loss (1)
- B. Gamble (2)

End of Block: Gamble With Loss - Task 9

Start of Block: Gamble With Loss - Task 10

Q302 Imagine you have chosen "sure loss" for  gambling task 10 and lost  $211. Rate the valence of your feelings (how positive or negative you feel) on the below scale:

- 1 (Strongly Negative) (1)
- 2 (2)
- 3 (3)
- 4(Neutral) (4)
- 5 (5)
- 6 (6)
- 7 (Strongly Positive) (7)

Q304 Imagine you have chosen "sure loss" for  gambling task 10 and lost  $211. Rate the intensity of your feelings on the below scale:

- 1 (Very Low) (1)
- 2 (2)
- 3 (3)
- 4 (4)
- 5 (5)
- 6 (6)
- 7 (Very High) (7)

Q306 Imagine you have already played gambling task 10 and lost $0. Rate the valence of your feelings (how positive or negative you feel) on the below scale:

- 1(Strongly Negative) (1)
- 2 (2)
- 3 (3)
- 4 (Neutral) (4)
- 5 (5)
- 6 (6)
- 7 (Strongly Positive) (7)

Q308 Imagine you have already played gambling task 10 and lost $0. Rate the intensity of your feelings on the below scale:

- 1 (Very Low) (1)
- 2 (2)
- 3 (3)
- 4 (4)
- 5 (5)
- 0 (6)
- 7 (Very High) (7)

Q310 Imagine you have already played gambling task 10  and lost $400. Rate the valence of your feelings (how positive or negative you feel) on the below scale:

- 1 (Strongly Negative) (1)
- 2 (2)
- 3 (3)
- 4 (Neutral) (4)
- 5 (5)
- 6 (6)
- 7 (Strongly Positive) (7)

Q312 Imagine you have already played gambling task 10 and lost $400. Rate the intensity of your feelings on the below scale:

- 1(Very Low) (1)
- 2 (2)
- 3 (3)
- 4 (4)
- 5 (5)
- 6 (6)
- 7 (Very High) (7)

Q314 Gambling Task 10: Imagine choosing between "losing $30 for certain" or a "50% chance of losing $100" (and hence there is a 50% chance of not losing anything). Which option would you choose?  Here you would have to imagine making choices between playing a gamble that can make you lose an amount of money and losing a smaller amount for sure. Each pair of options is again presented as two pie charts. Imagine that a spinner is attached to the center of the pie chart and after the spinner is spun you will lose the money written in the region where and the pointer lands. As you can see, the pie charts representing ‘Sure Loss’ will always make you lose a certain amount while the pie charts representing ‘Loss Gamble’ can make you lose either a bigger amount or zero (the two regions of these pie charts represent the probabilities for each loss respectively). Please circle the pie chart you would prefer (sure loss or gamble) in each pair. Note that there are no correct answers and your choice is a matter of personal preference, but try to choose which option (sure loss or gamble) you would prefer if this choice was made for real. 

- A. Sure Loss (1)
- B. Gamble (2)

End of Block: Gamble With Loss - Task 10

Start of Block: Gamble With Loss - Task 11

Q316 Imagine you have chosen "sure loss" for  gambling task 11 and lost  $98. Rate the valence of your feelings (how positive or negative you feel) on the below scale:

- 1(Strongly Negative) (1)
- 2 (2)
- 3 (3)
- 4 (Neutral) (4)
- 5 (5)
- 6 (6)
- 7 (Strongly Positive) (7)

Q318 Imagine you have chosen "sure loss" for  gambling task 11 and lost  $98. Rate the intensity of your feelings on the below scale:

- 1 (Very Low) (1)
- 2 (2)
- 3 (3)
- 4 (4)
- 5 (5)
- 6 (6)
- 7 (Very High) (7)

Q320 Imagine you have already played gambling task 11 and lost $0. Rate the valence of your feelings (how positive or negative you feel) on the below scale:

- 1 (Strongly Negative) (1)
- 2 (2)
- 3 (3)
- 4 (Neutral) (4)
- 5 (5)
- 6 (6)
- 7 (Strongly Positive) (7)

Q322 Imagine you have already played gambling task 11 and lost $0. Rate the intensity of your feelings on the below scale:

- 1 (Very Low) (1)
- 2 (2)
- 3 (3)
- 4 (4)
- 5 (5)
- 0 (6)
- 7 (Very High) (7)

Q324 Imagine you have already played gambling task 11 and lost $400. Rate the valence of your feelings (how positive or negative you feel) on the below scale:

- 1 (Strongly Negative) (1)
- 2 (2)
- 3 (3)
- 4 (Neutral) (4)
- 5 (5)
- 6 (6)
- 7 (Strongly Positive) (7)

Q326 Imagine you have already played gambling task 11 and lost $400. Rate the intensity of your feelings on the below scale:

- 1(Very Low) (1)
- 2 (2)
- 3 (3)
- 4 (4)
- 5 (5)
- 6 (6)
- 7 (Very High) (7)

Q328 Gambling Task 11: Imagine choosing between "losing $30 for certain" or a "50% chance of losing $100" (and hence there is a 50% chance of not losing anything). Which option would you choose?  Here you would have to imagine making choices between playing a gamble that can make you lose an amount of money and losing a smaller amount for sure. Each pair of options is again presented as two pie charts. Imagine that a spinner is attached to the center of the pie chart and after the spinner is spun you will lose the money written in the region where and the pointer lands. As you can see, the pie charts representing ‘Sure Loss’ will always make you lose a certain amount while the pie charts representing ‘Loss Gamble’ can make you lose either a bigger amount or zero (the two regions of these pie charts represent the probabilities for each loss respectively). Please circle the pie chart you would prefer (sure loss or gamble) in each pair. Note that there are no correct answers and your choice is a matter of personal preference, but try to choose which option (sure loss or gamble) you would prefer if this choice was made for real. 

- A. Sure Loss (1)
- B. Gamble (2)

End of Block: Gamble With Loss - Task 11

Start of Block: Gamble With Loss - Task 12

Q330 Imagine you have chosen "sure loss" for  gambling task 12 and lost  $211. Rate the valence of your feelings (how positive or negative you feel) on the below scale:

- 1 (Strongly Negative) (1)
- 2 (2)
- 3 (3)
- 4 (Neutral) (4)
- 5 (5)
- 6 (6)
- 7 (Strongly Positive) (7)

Q332 Imagine you have chosen "sure loss" for  gambling task 12 and lost  $211. Rate the intensity of your feelings on the below scale:

- 1 (Very Low) (1)
- 2 (2)
- 3 (3)
- 4 (4)
- 5 (5)
- 6 (6)
- 7 (Very High) (7)

Q334 Imagine you have already played gambling task 12 and lost $0. Rate the valence of your feelings (how positive or negative you feel) on the below scale:

- 1 (Strongly Negative) (1)
- 2 (2)
- 3 (3)
- 4 (Neutral) (4)
- 5 (5)
- 6 (6)
- 7 (Strongly Positive) (7)

Q336 Imagine you have already played gambling task 12 and lost  $0. Rate the intensity of your feelings on the below scale:

- 1 (Very Low) (1)
- 2 (2)
- 3 (3)
- 4 (4)
- 5 (5)
- 0 (6)
- 7 (Very High) (7)

Q338 Imagine you have already played gambling task 12 and lost  $400. Rate the valence of your feelings (how positive or negative you feel) on the below scale:

- 1 (Strongly Negative) (1)
- 2 (2)
- 3 (3)
- 4 (Neutral) (4)
- 5 (5)
- 6 (6)
- 7 (Strongly Positive) (7)

Q340 Imagine you have already played gambling task 12 and lost  $400. Rate the intensity of your feelings on the below scale:

- 1(Very Low) (1)
- 2 (2)
- 3 (3)
- 4 (4)
- 5 (5)
- 6 (6)
- 7 (Very High) (7)

Q342 Gambling Task 12: Imagine choosing between "losing $30 for certain" or a "50% chance of losing $100" (and hence there is a 50% chance of not losing anything). Which option would you choose?  Here you would have to imagine making choices between playing a gamble that can make you lose an amount of money and losing a smaller amount for sure. Each pair of options is again presented as two pie charts. Imagine that a spinner is attached to the center of the pie chart and after the spinner is spun you will lose the money written in the region where and the pointer lands. As you can see, the pie charts representing ‘Sure Loss’ will always make you lose a certain amount while the pie charts representing ‘Loss Gamble’ can make you lose either a bigger amount or zero (the two regions of these pie charts represent the probabilities for each loss respectively). Please circle the pie chart you would prefer (sure loss or gamble) in each pair. Note that there are no correct answers and your choice is a matter of personal preference, but try to choose which option (sure loss or gamble) you would prefer if this choice was made for real.

- A. Sure Loss (1)
- B. Gamble (2)

1. *Lotteries, anticipated positive*

Q259
SCENARIO: GAMBLE WITH GAINImagine choosing between "receiving $30 for certain" or a "50% chance of winning $100". Which option would you choose?  Here you would have to imagine making choices between playing a gamble to receive an amount of money and taking a smaller amount for sure. Each pair of options is presented as two pie charts. Imagine that a spinner is attached to the center of the pie chart and after the spinner is spun you will receive the money written in the region where and the pointer lands. As you can see, the pie charts representing ‘Sure Gain’ will always give you a certain amount while the pie charts representing ‘Gamble’ offer either a bigger amount or zero (the two regions of these pie charts represent the probabilities for each amount respectively).

Q18 Imagine you have chosen "sure gain" for  gambling task 1 and received $16. Rate the valence of your feelings (how positive or negative you feel) on the scale below: 

- 1 (Strongly Negative) (1)
- 2 (2)
- 3 (3)
- 4 (Neutral) (4)
- 5 (5)
- 6 (6)
- 7 ( Strongly Positive) (7)

Q20 Imagine you have chosen "sure gain" for  gambling task 1 and received $16. Rate the intensity of your feelings on the below scale:

- 1 (Very Low) (1)
- 2 (2)
- 3 (3)
- 4 (4)
- 5 (5)
- 6 (6)
- 7 (Very High) (7)

Q22 Imagine you have already played gambling task 1 and won $0. Rate the valence of your feelings (how positive or negative you feel) on the below scale:

- 1 (Strongly Negative) (1)
- 2 (2)
- 3 (3)
- 4 (Neutral) (4)
- 5 (5)
- 6 (6)
- 7(Strongly Positive) (7)

Q24 Imagine you have already played gambling task 1 and won $0. Rate the intensity of your feelings on the below scale:

- 1 (Very Low) (1)
- 2 (2)
- 3 (3)
- 4 (4)
- 5 (5)
- 6 (6)
- 7 (Very High) (7)

Q26 Imagine you have already played gambling task 1 and won $100. Rate the valence of your feelings (how positive or negative you feel) on the below scale:

- 1 (Strongly Negative) (1)
- 2 (2)
- 3 (3)
- 4 (Neutral) (4)
- 5 (5)
- 6 (6)
- 7 (Strongly Positive) (7)

Q28 Imagine you have already played gambling task 1 and won $100. Rate the intensity of your feelings on the below scale:

- 1 (Low) (1)
- 2 (2)
- 3 (3)
- 4 (4)
- 5 (5)
- 6 (6)
- 7 (High) (7)

Q30 Gambling Task 1:Imagine choosing between "receiving $30 for certain" or a "50% chance of winning $100". Which option would you choose?  Here you would have to imagine making choices between playing a gamble to receive an amount of money and taking a smaller amount for sure. Each pair of options is presented as two pie charts. Imagine that a spinner is attached to the center of the pie chart and after the spinner is spun you will receive the money written in the region where and the pointer lands. As you can see, the pie charts representing ‘Sure Gain’ will always give you a certain amount while the pie charts representing ‘Gamble’ offer either a bigger amount or zero (the two regions of these pie charts represent the probabilities for each amount respectively). Please circle the pie chart you would prefer (the sure amount or the gamble) in each pair. Note that there are no correct answers and your choice is a matter of personal preference, but try to choose which option (sure amount or a gamble) you would prefer if this choice was made for real.

- A. Sure Gain (1)
- B. Gamble (2)

End of Block: Gamble With Gain - Task 1

Start of Block: Gamble With Gain - Task 2

Q33 Imagine you have chosen "sure gain" for  gambling task 2 and received $46. Rate the valence of your feelings (how positive or negative you feel) on the below scale: 

- 1 (Strongly Negative) (1)
- 2 (2)
- 3 (3)
- 4 (Neutral) (4)
- 5 (5)
- 6 (6)
- 7 (Strongly Positive) (7)

Q35 Imagine you have chosen "sure gain" for  gambling task 2 and received $46. Rate the valence of your feelings (how positive or negative you feel) on the below scale: . Rate the intensity of your feelings on the below scale:

- 1 (Very Low) (1)
- 2 (2)
- 3 (3)
- 4 (4)
- 5 (5)
- 6 (6)
- 7 (Very High) (7)

Q37 Imagine you have already played gambling task 2 and won $0. Rate the valence of your feelings (how positive or negative you feel) on the below scale:

- 1 (Strongly Negative) (1)
- 2 (2)
- 3 (3)
- 4 (Neutral) (4)
- 5 (5)
- 6 (6)
- 7 (Strongly Positive) (7)

Q39 Imagine you have already played gambling task 2 and won $0. Rate the intensity of your feelings on the below scale:

- 1 (Very Low) (1)
- 2 (2)
- 3 (3)
- 4 (4)
- 5 (5)
- 6 (6)
- 7 (Very High) (7)

Q41 Imagine you have already played gambling task 2 and won $100. Rate the valence of your feelings (how positive or negative you feel) on the below scale:

- 1 (Strongly Negative) (1)
- 2 (2)
- 3 (3)
- 4 (Neutral) (4)
- 5 (5)
- 6 (6)
- 7 (Strongly Positive) (7)

Q43 Imagine you have already played gambling task 2 and won $100. Rate the intensity of your feelings on the below scale:

- 1 (Low) (1)
- 2 (2)
- 3 (3)
- 4 (4)
- 5 (5)
- 6 (6)
- 7 (High) (7)

Q45 Gambling Task 2:
Imagine choosing between "receiving $30 for certain" or a "50% chance of winning $100". Which option would you choose?  Here you would have to imagine making choices between playing a gamble to receive an amount of money and taking a smaller amount for sure. Each pair of options is presented as two pie charts. Imagine that a spinner is attached to the center of the pie chart and after the spinner is spun you will receive the money written in the region where and the pointer lands. As you can see, the pie charts representing ‘Sure Gain’ will always give you a certain amount while the pie charts representing ‘Gamble’ offer either a bigger amount or zero (the two regions of these pie charts represent the probabilities for each amount respectively). Please circle the pie chart you would prefer (the sure amount or the gamble) in each pair. Note that there are no correct answers and your choice is a matter of personal preference, but try to choose which option (sure amount or a gamble) you would prefer if this choice was made for real.

- A. Sure Gain (1)
- B. Gamble (2)

End of Block: Gamble With Gain - Task 2

Start of Block: Gamble With Gain - Task 3

Q48 Imagine you have chosen "sure gain" for  gambling task 3 and received $76. Rate the valence of your feelings (how positive or negative you feel) on the below scale:

- 1 (Strongly Negative) (1)
- 2 (2)
- 3 (3)
- 4 (Neutral) (4)
- 5 (5)
- 6 (6)
- 7 (Strongly Positive) (7)

Q50 Imagine you have chosen "sure gain" for  gambling task 3 and received $76. Rate the intensity of your feelings on the below scale:

- 1 (Very Low) (1)
- 2 (2)
- 3 (3)
- 4 (4)
- 5 (5)
- 6 (6)
- 7 (Very High) (7)

Q52 Imagine you have already played gambling task 3 and won $0. Rate the valence of your feelings (how positive or negative you feel) on the below scale:

- 1 (Strongly Negative) (1)
- 2 (2)
- 3 (3)
- 4 (Neutral) (4)
- 5 (5)
- 6 (6)
- 7 (Strongly Positive) (7)

Q54 Imagine you have already played gambling task 3 and won $0. Rate the intensity of your feelings on the below scale:

- 1 (Very Low) (1)
- 2 (2)
- 3 (3)
- 4 (4)
- 5 (5)
- 6 (6)
- 7 (Very High) (7)

Q56 Imagine you have already played gambling task 3 and won $100. Rate the valence of your feelings (how positive or negative you feel) on the below scale:

- 1 (Strongly Negative) (1)
- 2 (2)
- 3 (3)
- 4 (Neutral) (4)
- 5 (5)
- 6 (6)
- 7 (Strongly Positive) (7)

Q58 Imagine you have already played gambling task 3 and won $100. Rate the intensity of your feelings on the below scale:

- 1 (Low) (1)
- 2 (2)
- 3 (3)
- 4 (4)
- 5 (5)
- 6 (6)
- 7 (High) (7)

Q60 Gambling Task 3: Imagine choosing between "receiving $30 for certain" or a "50% chance of winning $100". Which option would you choose?  Here you would have to imagine making choices between playing a gamble to receive an amount of money and taking a smaller amount for sure. Each pair of options is presented as two pie charts. Imagine that a spinner is attached to the center of the pie chart and after the spinner is spun you will receive the money written in the region where and the pointer lands. As you can see, the pie charts representing ‘Sure Gain’ will always give you a certain amount while the pie charts representing ‘Gamble’ offer either a bigger amount or zero (the two regions of these pie charts represent the probabilities for each amount respectively). Please circle the pie chart you would prefer (the sure amount or the gamble) in each pair. Note that there are no correct answers and your choice is a matter of personal preference, but try to choose which option (sure amount or a gamble) you would prefer if this choice was made for real.

- A. Sure Gain (1)
- B. Gamble (2)

End of Block: Gamble With Gain - Task 3

Start of Block: Gamble With Gain - Task 4

Q76 Imagine you have chosen "sure gain" for  gambling task 4 and received $15. Rate the valence of your feelings (how positive or negative you feel) on the below scale: 

- 1 (Strongly Negative) (1)
- 2 (2)
- 3 (3)
- 4 (Neutral) (4)
- 5 (5)
- 6 (6)
- 7 (Strongly Positive) (7)

Q78 Imagine you have chosen "sure gain" for  gambling task 4 and received $15. Rate the intensity of your feelings on the below scale:

- 1 (Very Low) (1)
- 2 (2)
- 3 (3)
- 4 (4)
- 5 (5)
- 6 (6)
- 7 (Very High) (7)

Q80 Imagine you have already played gambling task 4 and won $0. Rate the valence of your feelings (how positive or negative you feel) on the below scale:

- 1 (Strongly Negative) (1)
- 2 (2)
- 3 (3)
- 4 (Neutral) (4)
- 5 (5)
- 6 (6)
- 7 (Strongly Positive) (7)

Q82 Imagine you have already played gambling task 4 and won $0. Rate the intensity of your feelings on the below scale:

- 1 (Very Low) (1)
- 2 (2)
- 3 (3)
- 4 (4)
- 5 (5)
- 6 (6)
- 7 (Very High) (7)

Q84 Imagine you have already played gambling task 4 and won $200. Rate the valence of your feelings (how positive or negative you feel) on the below scale:

- 1 (Strongly Negative) (1)
- 2 (2)
- 3 (3)
- 4 (Neutral) (4)
- 5 (5)
- 6 (6)
- 7 (Strongly Positive) (7)

Q86 Imagine you have already played gambling task 4 and won $200. Rate the intensity of your feelings on the below scale:

- 1 (Low) (1)
- 2 (2)
- 3 (3)
- 4 (4)
- 5 (5)
- 6 (6)
- 7 (High) (7)

Q88 Gambling Task 4: Imagine choosing between "receiving $30 for certain" or a "50% chance of winning $100". Which option would you choose?  Here you would have to imagine making choices between playing a gamble to receive an amount of money and taking a smaller amount for sure. Each pair of options is presented as two pie charts. Imagine that a spinner is attached to the center of the pie chart and after the spinner is spun you will receive the money written in the region where and the pointer lands. As you can see, the pie charts representing ‘Sure Gain’ will always give you a certain amount while the pie charts representing ‘Gamble’ offer either a bigger amount or zero (the two regions of these pie charts represent the probabilities for each amount respectively). Please circle the pie chart you would prefer (the sure amount or the gamble) in each pair. Note that there are no correct answers and your choice is a matter of personal preference, but try to choose which option (sure amount or a gamble) you would prefer if this choice was made for real.

- A. Sure Gain (1)
- B. Gamble (2)

End of Block: Gamble With Gain - Task 4

Start of Block: Gamble With Gain - Task 5

Q90 Imagine you have chosen "sure gain" for  gambling task 5 and received $72. Rate the valence of your feelings (how positive or negative you feel) on the below scale: 

- 1 (Strongly Negative) (1)
- 2 (2)
- 3 (3)
- 4 (Neutral) (4)
- 5 (5)
- 6 (6)
- 7 (Strongly Positive) (7)

Q92 Imagine you have chosen "sure gain" for  gambling task 5 and received $72. Rate the intensity of your feelings on the below scale:

- 1 (Very Low) (1)
- 2 (2)
- 3 (3)
- 4 (4)
- 5 (5)
- 6 (6)
- 7 (Very High) (7)

Q94 Imagine you have already played gambling task 5 and won $0. Rate the valence of your feelings (how positive or negative you feel) on the below scale:

- 1 (Strongly Negative) (1)
- 2 (2)
- 3 (3)
- 4 (Neutral) (4)
- 5 (5)
- 6 (6)
- 7 (Strongly Positive) (7)

Q96 Imagine you have already played gambling task 5 and won $0. Rate the intensity of your feelings on the below scale:

- 1 (Very Low) (1)
- 2 (2)
- 3 (3)
- 4 (4)
- 5 (5)
- 6 (6)
- 7 (Very High) (7)

Q98 Imagine you have already played gambling task 5 and won $200 Rate the valence of your feelings (how positive or negative you feel) on the below scale:

- 1 (Strongly Negative) (1)
- 2 (2)
- 3 (3)
- 4 (Neutral) (4)
- 5 (5)
- 6 (6)
- 7 (Strongly Positive) (7)

Q100 Imagine you have already played gambling task 5 and won $200. Rate the intensity of your feelings on the below scale:

- 1 (Low) (1)
- 2 (2)
- 3 (3)
- 4 (4)
- 5 (5)
- 6 (6)
- 7 (High) (7)

Q102 Gambling Task 5: Imagine choosing between "receiving $30 for certain" or a "50% chance of winning $100". Which option would you choose?  Here you would have to imagine making choices between playing a gamble to receive an amount of money and taking a smaller amount for sure. Each pair of options is presented as two pie charts. Imagine that a spinner is attached to the center of the pie chart and after the spinner is spun you will receive the money written in the region where and the pointer lands. As you can see, the pie charts representing ‘Sure Gain’ will always give you a certain amount while the pie charts representing ‘Gamble’ offer either a bigger amount or zero (the two regions of these pie charts represent the probabilities for each amount respectively). Please circle the pie chart you would prefer (the sure amount or the gamble) in each pair. Note that there are no correct answers and your choice is a matter of personal preference, but try to choose which option (sure amount or a gamble) you would prefer if this choice was made for real.

- A. Sure Gain (1)
- B. Gamble (2)

End of Block: Gamble With Gain - Task 5

Start of Block: Gamble with Gain - Task 6

Q105 Imagine you have chosen "sure gain" for  gambling task 6 and received $142. Rate the valence of your feelings (how positive or negative you feel) on the below scale: 

- 1 (Strongly Negative) (1)
- 2 (2)
- 3 (3)
- 4 (Neutral) (4)
- 5 (5)
- 6 (6)
- 7 (Strongly Positive) (7)

Q107 Imagine you have chosen "sure gain" for  gambling task 6 and received $142. Rate the intensity of your feelings on the below scale:

- 1 (Very Low) (1)
- 2 (2)
- 3 (3)
- 4 (4)
- 5 (5)
- 6 (6)
- 7 (Very High) (7)

Q109 Imagine you have already played gambling task 6 and won $0. Rate the valence of your feelings (how positive or negative you feel) on the below scale:

- 1 (Strongly Negative) (1)
- 2 (2)
- 3 (3)
- 4 (Neutral) (4)
- 5 (5)
- 6 (6)
- 7(Strongly Positive) (7)

Q111 Imagine you have already played gambling task 6 and won $0. Rate the intensity of your feelings on the below scale:

- 1 (Very Low) (1)
- 2 (2)
- 3 (3)
- 4 (4)
- 5 (5)
- 6 (6)
- 7 (Very High) (7)

Q113 Imagine you have already played gambling task 6 and won $200. Rate the valence of your feelings (how positive or negative you feel) on the below scale:

- 1 (Strongly Negative) (1)
- 2 (2)
- 3 (3)
- 4 (Neutral) (4)
- 5 (5)
- 6 (6)
- 7 (Strongly Positive) (7)

Q115 Imagine you have already played gambling task 6 and won $200. Rate the intensity of your feelings on the below scale:

- 1 (Low) (1)
- 2 (2)
- 3 (3)
- 4 (4)
- 5 (5)
- 6 (6)
- 7 (High) (7)

Q117 Gambling Task 6: Imagine choosing between "receiving $30 for certain" or a "50% chance of winning $100". Which option would you choose?  Here you would have to imagine making choices between playing a gamble to receive an amount of money and taking a smaller amount for sure. Each pair of options is presented as two pie charts. Imagine that a spinner is attached to the center of the pie chart and after the spinner is spun you will receive the money written in the region where and the pointer lands. As you can see, the pie charts representing ‘Gain’ will always give you a certain amount while the pie charts representing ‘Gamble’ offer either a bigger amount or zero (the two regions of these pie charts represent the probabilities for each amount respectively). Please circle the pie chart you would prefer (the sure amount or the gamble) in each pair. Note that there are no correct answers and your choice is a matter of personal preference, but try to choose which option (sure amount or a gamble) you would prefer if this choice was made for real.

- A. Sure Gain (1)
- B. Gamble (2)

End of Block: Gamble with Gain - Task 6

Start of Block: Gamble With Gain - Task 7

Q133 Imagine you have chosen "sure gain" for  gambling task 7 and received $95. Rate the valence of your feelings (how positive or negative you feel) on the below scale: 

- 1 (Strongly Negative) (1)
- 2 (2)
- 3 (3)
- 4 (Neutral) (4)
- 5 (5)
- 6 (6)
- 7 (Strongly Positive) (7)

Q135 Imagine you have chosen "sure gain" for  gambling task 7 and received $95. Rate the intensity of your feelings on the below scale:

- 1 (Very Low) (1)
- 2 (2)
- 3 (3)
- 4 (4)
- 5 (5)
- 6 (6)
- 7 (Very High) (7)

Q137 Imagine you have already played gambling task 7 and won $0. Rate the valence of your feelings (how positive or negative you feel) on the below scale:

- 1 (Strongly Negative) (1)
- 2 (2)
- 3 (3)
- 4 (Neutral) (4)
- 5 (5)
- 6 (6)
- 7 (Strongly Positive) (7)

Q139 Imagine you have already played gambling task 7 and won $0. Rate the intensity of your feelings on the below scale:

- 1 (Very Low) (1)
- 2 (2)
- 3 (3)
- 4 (4)
- 5 (5)
- 6 (6)
- 7 (Very High) (7)

Q141 Imagine you have already played gambling task 7 and won $300. Rate the valence of your feelings (how positive or negative you feel) on the below scale:

- 1 (Strongly Negative) (1)
- 2 (2)
- 3 (3)
- 4 (Neutral) (4)
- 5 (5)
- 6 (6)
- 7 (Strongly Positive) (7)

Q143 Imagine you have already played gambling task 7 and won $300. Rate the intensity of your feelings on the below scale:

- 1 (Low) (1)
- 2 (2)
- 3 (3)
- 4 (4)
- 5 (5)
- 6 (6)
- 7 (High) (7)

Q145 Gambling Task 7: Imagine choosing between "receiving $30 for certain" or a "50% chance of winning $100". Which option would you choose?  Here you would have to imagine making choices between playing a gamble to receive an amount of money and taking a smaller amount for sure. Each pair of options is presented as two pie charts. Imagine that a spinner is attached to the center of the pie chart and after the spinner is spun you will receive the money written in the region where and the pointer lands. As you can see, the pie charts representing ‘Sure Gain’ will always give you a certain amount while the pie charts representing ‘Gamble’ offer either a bigger amount or zero (the two regions of these pie charts represent the probabilities for each amount respectively). Please circle the pie chart you would prefer (the sure amount or the gamble) in each pair. Note that there are no correct answers and your choice is a matter of personal preference, but try to choose which option (sure amount or a gamble) you would prefer if this choice was made for real.

- A. Sure Gain (1)
- B. Gamble (2)

End of Block: Gamble With Gain - Task 7

Start of Block: Gamble With Gain - Task 8

Q147 Imagine you have chosen "sure gain" for  gambling task 8 and received $70. Rate the valence of your feelings (how positive or negative you feel) on the below scale: 

- 1 (Strongly Negative) (1)
- 2 (2)
- 3 (3)
- 4 (Neutral) (4)
- 5 (5)
- 6 (6)
- 7 (Strongly Positive) (7)

Q149 Imagine you have chosen "sure gain" for  gambling task 8 and received $70. Rate the intensity of your feelings on the below scale:

- 1 (Very Low) (1)
- 2 (2)
- 3 (3)
- 4 (4)
- 5 (5)
- 6 (6)
- 7 (Very High) (7)

Q151 Imagine you have already played gambling task 8 and won $0. Rate the valence of your feelings (how positive or negative you feel) on the below scale:

- 1 (Strongly Negative) (1)
- 2 (2)
- 3 (3)
- 4 (Neutral) (4)
- 5 (5)
- 6 (6)
- 7 (Strongly Positive) (7)

Q153 Imagine you have already played gambling task 8 and won $0. Rate the intensity of your feelings on the below scale:

- 1 (Very Low) (1)
- 2 (2)
- 3 (3)
- 4 (4)
- 5 (5)
- 6 (6)
- 7 (Very High) (7)

Q155 Imagine you have already played gambling task 8 and won $300. Rate the valence of your feelings (how positive or negative you feel) on the below scale:

- 1 (Strongly Negative) (1)
- 2 (2)
- 3 (3)
- 4 (Neutral) (4)
- 5 (5)
- 6 (6)
- 7 (Strongly Positive) (7)

Q157 Imagine you have already played gambling task 8 and won $300. Rate the intensity of your feelings on the below scale:

- 1 (Low) (1)
- 2 (2)
- 3 (3)
- 4 (4)
- 5 (5)
- 6 (6)
- 7 (High) (7)

Q159 Gambling Task 8: Imagine choosing between "receiving $30 for certain" or a "50% chance of winning $100". Which option would you choose?  Here you would have to imagine making choices between playing a gamble to receive an amount of money and taking a smaller amount for sure. Each pair of options is presented as two pie charts. Imagine that a spinner is attached to the center of the pie chart and after the spinner is spun you will receive the money written in the region where and the pointer lands. As you can see, the pie charts representing ‘Sure Gain’ will always give you a certain amount while the pie charts representing ‘Gamble’ offer either a bigger amount or zero (the two regions of these pie charts represent the probabilities for each amount respectively). Please circle the pie chart you would prefer (the sure amount or the gamble) in each pair. Note that there are no correct answers and your choice is a matter of personal preference, but try to choose which option (sure amount or a gamble) you would prefer if this choice was made for real.

- A. Sure Gain (1)
- B. Gamble (2)

End of Block: Gamble With Gain - Task 8

Start of Block: Gamble With Gain - Task 9

Q162 Imagine you have chosen "sure gain" for  gambling task 9 and received $192. Rate the valence of your feelings (how positive or negative you feel) on the below scale: 

- 1 (Strongly Negative) (1)
- 2 (2)
- 3 (3)
- 4 (Neutral) (4)
- 5 (5)
- 6 (6)
- 7 (Strongly Positive) (7)

Q164 Imagine you have chosen "sure gain" for  gambling task 9 and received $192. Rate the intensity of your feelings on the below scale:

- 1 (Very Low) (1)
- 2 (2)
- 3 (3)
- 4 (4)
- 5 (5)
- 6 (6)
- 7 (Very High) (7)

Q166 Imagine you have already played gambling task 9 and won $0. Rate the valence of your feelings (how positive or negative you feel) on the below scale:

- 1 (Strongly Negative) (1)
- 2 (2)
- 3 (3)
- 4 (Neutral) (4)
- 5 (5)
- 6 (6)
- 7 (Strongly Positive) (7)

Q168 Imagine you have already played gambling task 9 and won $0. Rate the intensity of your feelings on the below scale:

- 1 (Very Low) (1)
- 2 (2)
- 3 (3)
- 4 (4)
- 5 (5)
- 6 (6)
- 7 (Very High) (7)

Q170 Imagine you have already played gambling task 9 and won $300. Rate the valence of your feelings (how positive or negative you feel) on the below scale:

- 1 (Strongly Negative) (1)
- 2 (2)
- 3 (3)
- 4 (Neutral) (4)
- 5 (5)
- 6 (6)
- 7 (Strongly Positive) (7)

Q172 Imagine you have already played gambling task 9 and won $300. Rate the intensity of your feelings on the below scale:

- 1 (Low) (1)
- 2 (2)
- 3 (3)
- 4 (4)
- 5 (5)
- 6 (6)
- 7 (High) (7)

Q174 Gambling Task 9: Imagine choosing between "receiving $30 for certain" or a "50% chance of winning $100". Which option would you choose?  Here you would have to imagine making choices between playing a gamble to receive an amount of money and taking a smaller amount for sure. Each pair of options is presented as two pie charts. Imagine that a spinner is attached to the center of the pie chart and after the spinner is spun you will receive the money written in the region where and the pointer lands. As you can see, the pie charts representing ‘Sure Gain’ will always give you a certain amount while the pie charts representing ‘Gamble’ offer either a bigger amount or zero (the two regions of these pie charts represent the probabilities for each amount respectively). Please circle the pie chart you would prefer (the sure amount or the gamble) in each pair. Note that there are no correct answers and your choice is a matter of personal preference, but try to choose which option (sure amount or a gamble) you would prefer if this choice was made for real.

- A. Sure Gain (1)
- B. Gamble (2)

End of Block: Gamble With Gain - Task 9

Start of Block: Gamble With Gain - Task 10

Q190 Imagine you have chosen "sure gain" for  gambling task 10 and received $98. Rate the valence of your feelings (how positive or negative you feel) on the below scale: 

- 1 (Strongly Negative) (1)
- 2 (2)
- 3 (3)
- 4 (Neutral) (4)
- 5 (5)
- 6 (6)
- 7 (Strongly Positive) (7)

Q192 Imagine you have chosen "sure gain" for  gambling task 10 and received $98. Rate the intensity of your feelings on the below scale:

- 1 (Very Low) (1)
- 2 (2)
- 3 (3)
- 4 (4)
- 5 (5)
- 6 (6)
- 7 (Very High) (7)

Q194 Imagine you have already played gambling task 10 and won $0. Rate the valence of your feelings (how positive or negative you feel) on the below scale:

- 1 (Strongly Negative) (1)
- 2 (2)
- 3 (3)
- 4(Neutral) (4)
- 5 (5)
- 6 (6)
- 7 (Strongly Positive) (7)

Q196 Imagine you have already played gambling task 10 and won $0. Rate the intensity of your feelings on the below scale:

- 1 (Very Low) (1)
- 2 (2)
- 3 (3)
- 4 (4)
- 5 (5)
- 6 (6)
- 7 (Very High) (7)

Q198 Imagine you have already played gambling task 10 and won $400. Rate the valence of your feelings (how positive or negative you feel) on the below scale:

- 1 (Strongly Negative) (1)
- 2 (2)
- 3 (3)
- 4 (Neutral) (4)
- 5 (5)
- 6 (6)
- 7 (Strongly Positive) (7)

Q200 Imagine you have already played gambling task 10 and won $400. Rate the intensity of your feelings on the below scale:

- 1 (Low) (1)
- 2 (2)
- 3 (3)
- 4 (4)
- 5 (5)
- 6 (6)
- 7 (High) (7)

Q202 Gambling Task 10: Imagine choosing between "receiving $30 for certain" or a "50% chance of winning $100". Which option would you choose?  Here you would have to imagine making choices between playing a gamble to receive an amount of money and taking a smaller amount for sure. Each pair of options is presented as two pie charts. Imagine that a spinner is attached to the center of the pie chart and after the spinner is spun you will receive the money written in the region where and the pointer lands. As you can see, the pie charts representing ‘Sure Gain’ will always give you a certain amount while the pie charts representing ‘Gamble’ offer either a bigger amount or zero (the two regions of these pie charts represent the probabilities for each amount respectively). Please circle the pie chart you would prefer (the sure amount or the gamble) in each pair. Note that there are no correct answers and your choice is a matter of personal preference, but try to choose which option (sure amount or a gamble) you would prefer if this choice was made for real.

- A. Sure Gain (1)
- B. Gamble (2)

End of Block: Gamble With Gain - Task 10

Start of Block: Gamble With Gain - Task 11

Q204 Imagine you have chosen "sure gain" for  gambling task 11 and received $211. Rate the valence of your feelings (how positive or negative you feel) on the below scale: 

- 1 (Strongly Negative) (1)
- 2 (2)
- 3 (3)
- 4 (Neutral) (4)
- 5 (5)
- 6 (6)
- 7 (Strongly Positive) (7)

Q206 Imagine you have chosen "sure gain" for  gambling task 11 and received $211. Rate the intensity of your feelings on the below scale:

- 1 (Very Low) (1)
- 2 (2)
- 3 (3)
- 4 (4)
- 5 (5)
- 6 (6)
- 7 (Very High) (7)

Q208 Imagine you have already played gambling task 11 and won $0. Rate the valence of your feelings (how positive or negative you feel) on the below scale:

- 1 (Strongly Negative) (1)
- 2 (2)
- 3 (3)
- 4 (Neutral) (4)
- 5 (5)
- 6 (6)
- 7 (Strongly Positive) (7)

Q210 Imagine you have already played gambling task 11 and won $0. Rate the intensity of your feelings on the below scale:

- 1 (Very Low) (1)
- 2 (2)
- 3 (3)
- 4 (4)
- 5 (5)
- 6 (6)
- 7 (Very High) (7)

Q212 Imagine you have already played gambling task 11 and won $400. Rate the valence of your feelings (how positive or negative you feel) on the below scale:

- 1 (Strongly Negative) (1)
- 2 (2)
- 3 (3)
- 4 (Neutral) (4)
- 5 (5)
- 6 (6)
- 7 (Strongly Positive) (7)

Q214 Imagine you have already played gambling task 11 and won $400. Rate the intensity of your feelings on the below scale:

- 1 (Low) (1)
- 2 (2)
- 3 (3)
- 4 (4)
- 5 (5)
- 6 (6)
- 7 (High) (7)

Q216 Gambling Task 11: Imagine choosing between "receiving $30 for certain" or a "50% chance of winning $100". Which option would you choose?  Here you would have to imagine making choices between playing a gamble to receive an amount of money and taking a smaller amount for sure. Each pair of options is presented as two pie charts. Imagine that a spinner is attached to the center of the pie chart and after the spinner is spun you will receive the money written in the region where and the pointer lands. As you can see, the pie charts representing ‘Sure Gain’ will always give you a certain amount while the pie charts representing ‘Gamble’ offer either a bigger amount or zero (the two regions of these pie charts represent the probabilities for each amount respectively). Please circle the pie chart you would prefer (the sure amount or the gamble) in each pair. Note that there are no correct answers and your choice is a matter of personal preference, but try to choose which option (sure amount or a gamble) you would prefer if this choice was made for real.

- A. Sure Gain (1)
- B. Gamble (2)

End of Block: Gamble With Gain - Task 11

Start of Block: Gamble With Gain - Task 12

Q218 Imagine you have chosen "sure gain" for  gambling task 12 and received $211. Rate the valence of your feelings (how positive or negative you feel) on the below scale: 

- 1 (Strongly Negative) (1)
- 2 (2)
- 3 (3)
- 4 (Neutral) (4)
- 5 (5)
- 6 (6)
- 7 (Strongly Positive) (7)

Q220 Imagine you have chosen "sure gain" for  gambling task 12 and received $211. Rate the intensity of your feelings on the below scale:

- 1 (Very Low) (1)
- 2 (2)
- 3 (3)
- 4 (4)
- 5 (5)
- 6 (6)
- 7 (Very High) (7)

Q222 Imagine you have already played gambling task 12 and won $0. Rate the valence of your feelings (how positive or negative you feel) on the below scale:

- 1 (Strongly Negative) (1)
- 2 (2)
- 3 (3)
- 4 (Neutral) (4)
- 5 (5)
- 6 (6)
- 7 (Strongly Positive) (7)

Q224 Imagine you have already played gambling task 12 and won $0. Rate the intensity of your feelings on the below scale:

- 1 (Very Low) (1)
- 2 (2)
- 3 (3)
- 4 (4)
- 5 (5)
- 6 (6)
- 7 (Very High) (7)

Q226 Imagine you have already played gambling task 12 and won $400. Rate the valence of your feelings (how positive or negative you feel) on the below scale:

- 1 (Strongly Negative) (1)
- 2 (2)
- 3 (3)
- 4 (Neutral) (4)
- 5 (5)
- 6 (6)
- 7 (Strongly Positive) (7)

Q228 Imagine you have already played gambling task 12 and won $400. Rate the intensity of your feelings on the below scale:

- 1 (Low) (1)
- 2 (2)
- 3 (3)
- 4 (4)
- 5 (5)
- 6 (6)
- 7 (High) (7)

Q230 Gambling Task 12: Imagine choosing between "receiving $30 for certain" or a "50% chance of winning $100". Which option would you choose?  Here you would have to imagine making choices between playing a gamble to receive an amount of money and taking a smaller amount for sure. Each pair of options is presented as two pie charts. Imagine that a spinner is attached to the center of the pie chart and after the spinner is spun you will receive the money written in the region where and the pointer lands. As you can see, the pie charts representing ‘Sure Gain’ will always give you a certain amount while the pie charts representing ‘Gamble’ offer either a bigger amount or zero (the two regions of these pie charts represent the probabilities for each amount respectively). Please circle the pie chart you would prefer (the sure amount or the gamble) in each pair. Note that there are no correct answers and your choice is a matter of personal preference, but try to choose which option (sure amount or a gamble) you would prefer if this choice was made for real.

- A. Sure Gain (1)
- B. Gamble (2)

1. Anticipatory negative

Q134 Imagine you are about to play gambling task 1. How would you feel if you choose the "sure loss" of  $16? Rate the valence of your feelings (how positive or negative you feel) on the below scale:

- 1 (Strongly Negative) (1)
- 2 (2)
- 3 (3)
- 4 (Neutral) (4)
- 5 (5)
- 6 (6)
- 7 (Strongly Positive) (7)

Q136 Imagine you are about to play gambling task 1. How would you feel if you choose the "sure loss" of  $16? Rate the intensity of your feelings on the below scale:

- 1 (Very Low) (1)
- 2 (2)
- 3 (3)
- 4 (4)
- 5 (5)
- 6 (6)
- 7 (Very High) (7)

Q138 Imagine you are about to play gambling task 1. How would you feel if you lose $0? Rate the valence of your feelings (how positive or negative you feel) on the below scale:

- 1 (Strongly Negative) (1)
- 2 (2)
- 3 (3)
- 4 (Neutral) (4)
- 5 (5)
- 6 (6)
- 7 (Strongly Positive) (7)

Q140 Imagine you are about to play gambling task 1. How would you feel if you lose $0? Rate the intensity of your feelings on the below scale:

- 1 (Very Low) (1)
- 2 (2)
- 3 (3)
- 4 (4)
- 5 (5)
- 0 (6)
- 7 (Very High) (7)

Q142 Imagine you are about to play gambling task 1. How would you feel if you lose $100? Rate the valence of your feelings (how positive or negative you feel) on the below scale:

- 1 (Strongly Negative) (1)
- 2 (2)
- 3 (3)
- 4 (Neutral) (4)
- 5 (5)
- 6 (6)
- 7 (Strongly Positive) (7)

Q144 Imagine you are about to play gambling task 1. How would you feel if you lose $100? Rate the intensity of your feelings on the below scale:

- 1(Very Low) (1)
- 2 (2)
- 3 (3)
- 4 (4)
- 5 (5)
- 6 (6)
- 7 (Very High) (7)

Q146 Gambling Task 1: Imagine choosing between "losing $30 for certain" or a "50% chance of losing $100" (and hence there is a 50% chance of not losing anything). Which option would you choose?  Here you would have to imagine making choices between playing a gamble that can make you lose an amount of money and losing a smaller amount for sure. Each pair of options is again presented as two pie charts. Imagine that a spinner is attached to the center of the pie chart and after the spinner is spun you will lose the money written in the region where and the pointer lands. As you can see, the pie charts representing ‘Sure Loss’ will always make you lose a certain amount while the pie charts representing ‘Loss Gamble’ can make you lose either a bigger amount or zero (the two regions of these pie charts represent the probabilities for each loss respectively). Please circle the pie chart you would prefer (sure loss or gamble) in each pair. Note that there are no correct answers and your choice is a matter of personal preference, but try to choose which option (sure loss or gamble) you would prefer if this choice was made for real.

- A. Sure Loss (1)
- B. Gamble (2)

End of Block: Gamble With Loss - Task 1

Start of Block: Gamble With Loss - Task 2

Q148 Imagine you are about to play gambling task 2. How would you feel if you choose the "sure loss" of  $46? Rate the valence of your feelings (how positive or negative you feel) on the below scale:

- 1 (Strongly Negative) (1)
- 2 (2)
- 3 (3)
- 4 (Neutral) (4)
- 5 (5)
- 6 (6)
- 7 (Strongly Positive) (7)

Q150 Imagine you are about to play gambling task 2. How would you feel if you choose the "sure loss" of  $46? Rate the intensity of your feelings on the below scale:

- 1 (Very Low) (1)
- 2 (2)
- 3 (3)
- 4 (4)
- 5 (5)
- 6 (6)
- 7 (Very High) (7)

Q152 Imagine you are about to play gambling task 2. How would you feel if you lose $0? Rate the valence of your feelings (how positive or negative you feel) on the below scale:

- 1 (Strongly Negative) (1)
- 2 (2)
- 3 (3)
- 4 (Neutral) (4)
- 5 (5)
- 6 (6)
- 7 (Strongly Positive) (7)

Q154 Imagine you are about to play gambling task 2. How would you feel if you lose $0? Rate the intensity of your feelings on the below scale:

- 1 (Very Low) (1)
- 2 (2)
- 3 (3)
- 4 (4)
- 5 (5)
- 0 (6)
- 7 (Very High) (7)

Q156 Imagine you are about to play gambling task 2. How would you feel if you lose $100? Rate the valence of your feelings (how positive or negative you feel) on the below scale:

- 1 (Strongly Negative) (1)
- 2 (2)
- 3 (3)
- 4 (Neutral) (4)
- 5 (5)
- 6 (6)
- 7 (Strongly Positive) (7)

Q158 Imagine you are about to play gambling task 2. How would you feel if you lose $100?  Rate the intensity of your feelings on the below scale:

- 1(Very Low) (1)
- 2 (2)
- 3 (3)
- 4 (4)
- 5 (5)
- 6 (6)
- 7 (Very High) (7)

Q160 Gambling Task 2:  Imagine choosing between "losing $30 for certain" or a "50% chance of losing $100" (and hence there is a 50% chance of not losing anything). Which option would you choose?  Here you would have to imagine making choices between playing a gamble that can make you lose an amount of money and losing a smaller amount for sure. Each pair of options is again presented as two pie charts. Imagine that a spinner is attached to the center of the pie chart and after the spinner is spun you will lose the money written in the region where and the pointer lands. As you can see, the pie charts representing ‘Sure Loss’ will always make you lose a certain amount while the pie charts representing ‘Loss Gamble’ can make you lose either a bigger amount or zero (the two regions of these pie charts represent the probabilities for each loss respectively). Please circle the pie chart you would prefer (sure loss or gamble) in each pair. Note that there are no correct answers and your choice is a matter of personal preference, but try to choose which option (sure loss or gamble) you would prefer if this choice was made for real. 

- A. Sure Loss (1)
- B. Gamble (2)

End of Block: Gamble With Loss - Task 2

Start of Block: Gamble With Loss - Task 3

Q162 Imagine you are about to play gambling task 3. How would you feel if you choose the "sure loss" of  $76? Rate the valence of your feelings (how positive or negative you feel) on the below scale:

- 1 (Strongly Negative) (1)
- 2 (2)
- 3 (3)
- 4 (Neutral) (4)
- 5 (5)
- 6 (6)
- 7 (Strongly Positive) (7)

Q164 Imagine you are about to play gambling task 3. How would you feel if you choose the "sure loss" of  $76?  Rate the intensity of your feelings on the below scale:

- 1 (Very Low) (1)
- 2 (2)
- 3 (3)
- 4 (4)
- 5 (5)
- 6 (6)
- 7 (Very High) (7)

Q166 Imagine you are about to play gambling task 3. How would you feel if you lose $0? Rate the valence of your feelings (how positive or negative you feel) on the below scale:

- 1 (Strongly Negative) (1)
- 2 (2)
- 3 (3)
- 4 (Neutral) (4)
- 5 (5)
- 6 (6)
- 7 (Strongly Positive) (7)

Q168 Imagine you are about to play gambling task 3. How would you feel if you lose $0? Rate the intensity of your feelings on the below scale:

- 1 (Very Low) (1)
- 2 (2)
- 3 (3)
- 4 (4)
- 5 (5)
- 0 (6)
- 7 (Very High) (7)

Q170 Imagine you are about to play gambling task 3. How would you feel if you lose $100? Rate the valence of your feelings (how positive or negative you feel) on the below scale:

- 1(Strongly Negative) (1)
- 2 (2)
- 3 (3)
- 4 (Neutral) (4)
- 5 (5)
- 6 (6)
- 7 (Strongly Positive) (7)

Q172 Imagine you are about to play gambling task 3. How would you feel if you lose $100? Rate the intensity of your feelings on the below scale:

- 1(Very Low) (1)
- 2 (2)
- 3 (3)
- 4 (4)
- 5 (5)
- 6 (6)
- 7 (Very High) (7)

Q174 Gambling Task 3: Imagine choosing between "losing $30 for certain" or a "50% chance of losing $100" (and hence there is a 50% chance of not losing anything). Which option would you choose?  Here you would have to imagine making choices between playing a gamble that can make you lose an amount of money and losing a smaller amount for sure. Each pair of options is again presented as two pie charts. Imagine that a spinner is attached to the center of the pie chart and after the spinner is spun you will lose the money written in the region where and the pointer lands. As you can see, the pie charts representing ‘Sure Loss’ will always make you lose a certain amount while the pie charts representing ‘Loss Gamble’ can make you lose either a bigger amount or zero (the two regions of these pie charts represent the probabilities for each loss respectively). Please circle the pie chart you would prefer (sure loss or gamble) in each pair. Note that there are no correct answers and your choice is a matter of personal preference, but try to choose which option (sure loss or gamble) you would prefer if this choice was made for real. 

- A. Sure Loss (1)
- B. Gamble (2)

End of Block: Gamble With Loss - Task 3

Start of Block: Gamble With Loss - Task 4

Q190 Imagine you are about to play gambling task 4. How would you feel if you choose the "sure loss" of  $15? Rate the valence of your feelings (how positive or negative you feel) on the below scale:

- 1 (Strongly Negative) (1)
- 2 (2)
- 3 (3)
- 4 (Neutral) (4)
- 5 (5)
- 6 (6)
- 7 (Strongly Positive) (7)

Q192 Imagine you are about to play gambling task 4. How would you feel if you choose the "sure loss" of  $15? Rate the intensity of your feelings on the below scale:

- 1 (Very Low) (1)
- 2 (2)
- 3 (3)
- 4 (4)
- 5 (5)
- 6 (6)
- 7 (Very High) (7)

Q194 Imagine you are about to play gambling task 4. How would you feel if you lose $0? Rate the valence of your feelings (how positive or negative you feel) on the below scale:

- 1 (Strongly Negative) (1)
- 2 (2)
- 3 (3)
- 4 (Neutral) (4)
- 5 (5)
- 6 (6)
- 7 (Strongly Positive) (7)

Q196 Imagine you are about to play gambling task 4. How would you feel if you lose $0? Rate the intensity of your feelings on the below scale:

- 1 (Very Low) (1)
- 2 (2)
- 3 (3)
- 4 (4)
- 5 (5)
- 0 (6)
- 7 (Very High) (7)

Q198 Imagine you are about to play gambling task 4. How would you feel if you lose $200? Rate the valence of your feelings (how positive or negative you feel) on the below scale:

- 1 (Strongly Negative) (1)
- 2 (2)
- 3 (3)
- 4 (Neutral) (4)
- 5 (5)
- 6 (6)
- 7 (Strongly Positive) (7)

Q200 Imagine you are about to play gambling task 4. How would you feel if you lose $200? Rate the intensity of your feelings on the below scale:

- 1(Very Low) (1)
- 2 (2)
- 3 (3)
- 4 (4)
- 5 (5)
- 6 (6)
- 7 (Very High) (7)

Q202 Gambling Task 4:  Imagine choosing between "losing $30 for certain" or a "50% chance of losing $100" (and hence there is a 50% chance of not losing anything). Which option would you choose?  Here you would have to imagine making choices between playing a gamble that can make you lose an amount of money and losing a smaller amount for sure. Each pair of options is again presented as two pie charts. Imagine that a spinner is attached to the center of the pie chart and after the spinner is spun you will lose the money written in the region where and the pointer lands. As you can see, the pie charts representing ‘Sure Loss’ will always make you lose a certain amount while the pie charts representing ‘Loss Gamble’ can make you lose either a bigger amount or zero (the two regions of these pie charts represent the probabilities for each loss respectively). Please circle the pie chart you would prefer (sure loss or gamble) in each pair. Note that there are no correct answers and your choice is a matter of personal preference, but try to choose which option (sure loss or gamble) you would prefer if this choice was made for real. 

- A. Sure Loss (1)
- B. Gamble (2)

End of Block: Gamble With Loss - Task 4

Start of Block: Gamble With Loss - Task 5

Q204 Imagine you are about to play gambling task 5. How would you feel if you choose the "sure loss" of  $72? Rate the valence of your feelings (how positive or negative you feel) on the below scale:

- 1 (Strongly Negative) (1)
- 2 (2)
- 3 (3)
- 4 (Neutral) (4)
- 5 (5)
- 6 (6)
- 7 (Strongly Positive) (7)

Q206 Imagine you are about to play gambling task 5. How would you feel if you choose the "sure loss" of  $72? Rate the intensity of your feelings on the below scale:

- 1 (Very Low) (1)
- 2 (2)
- 3 (3)
- 4 (4)
- 5 (5)
- 6 (6)
- 7 (Very High) (7)

Q208 Imagine you are about to play gambling task 5. How would you feel if you lose $0? Rate the valence of your feelings (how positive or negative you feel) on the below scale:

- 1 (Strongly Negative) (1)
- 2 (2)
- 3 (3)
- 4 (Neutral) (4)
- 5 (5)
- 6 (6)
- 7 (Strongly Positive) (7)

Q210 Imagine you are about to play gambling task 5. How would you feel if you lose $0? Rate the intensity of your feelings on the below scale:

- 1 (Very Low) (1)
- 2 (2)
- 3 (3)
- 4 (4)
- 5 (5)
- 0 (6)
- 7 (Very High) (7)

Q212 Imagine you are about to play gambling task 5. How would you feel if you lose $200? Rate the valence of your feelings (how positive or negative you feel) on the below scale:

- 1 (Strongly Negative) (1)
- 2 (2)
- 3 (3)
- 4 (Neutral) (4)
- 5 (5)
- 6 (6)
- 7 (Strongly Positive) (7)

Q214 Imagine you are about to play gambling task 5. How would you feel if you lose $200? Rate the intensity of your feelings on the below scale:

- 1(Very Low) (1)
- 2 (2)
- 3 (3)
- 4 (4)
- 5 (5)
- 6 (6)
- 7 (Very High) (7)

Q216 Gambling Task 5: Imagine choosing between "losing $30 for certain" or a "50% chance of losing $100" (and hence there is a 50% chance of not losing anything). Which option would you choose?  Here you would have to imagine making choices between playing a gamble that can make you lose an amount of money and losing a smaller amount for sure. Each pair of options is again presented as two pie charts. Imagine that a spinner is attached to the center of the pie chart and after the spinner is spun you will lose the money written in the region where and the pointer lands. As you can see, the pie charts representing ‘Sure Loss’ will always make you lose a certain amount while the pie charts representing ‘Loss Gamble’ can make you lose either a bigger amount or zero (the two regions of these pie charts represent the probabilities for each loss respectively). Please circle the pie chart you would prefer (sure loss or gamble) in each pair. Note that there are no correct answers and your choice is a matter of personal preference, but try to choose which option (sure loss or gamble) you would prefer if this choice was made for real. 

- A. Sure Loss (1)
- B. Gamble (2)

End of Block: Gamble With Loss - Task 5

Start of Block: Gamble With Loss - Task 6

Q232 Imagine you are about to play gambling task 6. How would you feel if you choose the "sure loss" of  $142? Rate the valence of your feelings (how positive or negative you feel) on the below scale:

- 1 (Strongly Negative) (1)
- 2 (2)
- 3 (3)
- 4 (Neutral) (4)
- 5 (5)
- 6 (6)
- 7 (Strongly Positive) (7)

Q234 Imagine you are about to play gambling task 6. How would you feel if you choose the "sure loss" of  $142? Rate the intensity of your feelings on the below scale:

- 1 (Very Low) (1)
- 2 (2)
- 3 (3)
- 4 (4)
- 5 (5)
- 6 (6)
- 7 (Very High) (7)

Q236 Imagine you are about to play gambling task 6. How would you feel if you lose $0? Rate the valence of your feelings (how positive or negative you feel) on the below scale:

- 1 (Strongly Negative) (1)
- 2 (2)
- 3 (3)
- 4 (Neutral) (4)
- 5 (5)
- 6 (6)
- 7 (Strongly Positive) (7)

Q238 Imagine you are about to play gambling task 6. How would you feel if you lose $0? Rate the intensity of your feelings on the below scale:

- 1 (Very Low) (1)
- 2 (2)
- 3 (3)
- 4 (4)
- 5 (5)
- 0 (6)
- 7 (Very High) (7)

Q240 Imagine you are about to play gambling task 6. How would you feel if you lose $200? Rate the valence of your feelings (how positive or negative you feel) on the below scale:

- 1 (Strongly Negative) (1)
- 2 (2)
- 3 (3)
- 4 (Neutral) (4)
- 5 (5)
- 6 (6)
- 7 (Strongly Positive) (7)

Q242 Imagine you are about to play gambling task 6. How would you feel if you lose $200? Rate the intensity of your feelings on the below scale:

- 1(Very Low) (1)
- 2 (2)
- 3 (3)
- 4 (4)
- 5 (5)
- 6 (6)
- 7 (Very High) (7)

Q244 Gambling Task 6: Imagine choosing between "losing $30 for certain" or a "50% chance of losing $100" (and hence there is a 50% chance of not losing anything). Which option would you choose?  Here you would have to imagine making choices between playing a gamble that can make you lose an amount of money and losing a smaller amount for sure. Each pair of options is again presented as two pie charts. Imagine that a spinner is attached to the center of the pie chart and after the spinner is spun you will lose the money written in the region where and the pointer lands. As you can see, the pie charts representing ‘Sure Loss’ will always make you lose a certain amount while the pie charts representing ‘Loss Gamble’ can make you lose either a bigger amount or zero (the two regions of these pie charts represent the probabilities for each loss respectively). Please circle the pie chart you would prefer (sure loss or gamble) in each pair. Note that there are no correct answers and your choice is a matter of personal preference, but try to choose which option (sure loss or gamble) you would prefer if this choice was made for real. 

- A. Sure Loss (1)
- B. Gamble (2)

End of Block: Gamble With Loss - Task 6

Start of Block: Gamble With Loss - Task 7

Q246 Imagine you are about to play gambling task 7. How would you feel if you choose the "sure loss" of  $95?  Rate the valence of your feelings (how positive or negative you feel) on the below scale:

- 1 (Strongly Negative) (1)
- 2 (2)
- 3 (3)
- 4 (Neutral) (4)
- 5 (5)
- 6 (6)
- 7 (Strongly Positive) (7)

Q248 Imagine you are about to play gambling task 7. How would you feel if you choose the "sure loss" of  $95? Imagine you have already played the gamble and won $100. Rate the intensity of your feelings on the below scale:

- 1 (Very Low) (1)
- 2 (2)
- 3 (3)
- 4 (4)
- 5 (5)
- 6 (6)
- 7 (Very High) (7)

Q250 Imagine you are about to play gambling task 7. How would you feel if you lose $0? Rate the valence of your feelings (how positive or negative you feel) on the below scale:

- 1 (Strongly Negative) (1)
- 2 (2)
- 3 (3)
- 4 (Neutral) (4)
- 5 (5)
- 6 (6)
- 7 (Strongly Positive) (7)

Q252 Imagine you are about to play gambling task 7. How would you feel if you lose $0? Rate the intensity of your feelings on the below scale:

- 1 (Very Low) (1)
- 2 (2)
- 3 (3)
- 4 (4)
- 5 (5)
- 0 (6)
- 7 (Very High) (7)

Q254 Imagine you are about to play gambling task 7. How would you feel if you lose $300? Rate the valence of your feelings (how positive or negative you feel) on the below scale:

- 1 (Strongly Negative) (1)
- 2 (2)
- 3 (3)
- 4 (Neutral) (4)
- 5 (5)
- 6 (6)
- 7 (Strongly Positive) (7)

Q256 Imagine you are about to play gambling task 7. How would you feel if you lose $300? Rate the intensity of your feelings on the below scale:

- 1(Very Low) (1)
- 2 (2)
- 3 (3)
- 4 (4)
- 5 (5)
- 6 (6)
- 7 (Very High) (7)

Q258 Gambling Task 7:  Imagine choosing between "losing $30 for certain" or a "50% chance of losing $100" (and hence there is a 50% chance of not losing anything). Which option would you choose?  Here you would have to imagine making choices between playing a gamble that can make you lose an amount of money and losing a smaller amount for sure. Each pair of options is again presented as two pie charts. Imagine that a spinner is attached to the center of the pie chart and after the spinner is spun you will lose the money written in the region where and the pointer lands. As you can see, the pie charts representing ‘Sure Loss’ will always make you lose a certain amount while the pie charts representing ‘Loss Gamble’ can make you lose either a bigger amount or zero (the two regions of these pie charts represent the probabilities for each loss respectively). Please circle the pie chart you would prefer (sure loss or gamble) in each pair. Note that there are no correct answers and your choice is a matter of personal preference, but try to choose which option (sure loss or gamble) you would prefer if this choice was made for real. 

- A. Sure Loss (1)
- B. Gamble (2)

End of Block: Gamble With Loss - Task 7

Start of Block: Gamble With Loss - Task 8

Q260 Imagine you are about to play gambling task 8. How would you feel if you choose the "sure loss" of  $70? Rate the valence of your feelings (how positive or negative you feel) on the below scale:

- 1 (Strongly Negative) (1)
- 2 (2)
- 3 (3)
- 4 (Neutral) (4)
- 5 (5)
- 6 (6)
- 7 (Strongly Positive) (7)

Q262 Imagine you are about to play gambling task 8. How would you feel if you choose the "sure loss" of  $70? Rate the intensity of your feelings on the below scale:

- 1 (Very Low) (1)
- 2 (2)
- 3 (3)
- 4 (4)
- 5 (5)
- 6 (6)
- 7 (Very High) (7)

Q264 Imagine you are about to play gambling task 8. How would you feel if you lose $0? Rate the valence of your feelings (how positive or negative you feel) on the below scale:

- 1 (Strongly Negative) (1)
- 2 (2)
- 3 (3)
- 4 (Neutral) (4)
- 5 (5)
- 6 (6)
- 7 (Strongly Positive) (7)

Q266 Imagine you are about to play gambling task 8. How would you feel if you lose $0? Rate the intensity of your feelings on the below scale:

- 1 (Very Low) (1)
- 2 (2)
- 3 (3)
- 4 (4)
- 5 (5)
- 0 (6)
- 7 (Very High) (7)

Q268 Imagine you are about to play gambling task 8. How would you feel if you lose $300? Rate the valence of your feelings (how positive or negative you feel) on the below scale:

- 1 (Strongly Negative) (1)
- 2 (2)
- 3 (3)
- 4 (Neutral) (4)
- 5 (5)
- 6 (6)
- 7 (Strongly Positive) (7)

Q270 Imagine you are about to play gambling task 8. How would you feel if you lose $300? Rate the intensity of your feelings on the below scale:

- 1(Very Low) (1)
- 2 (2)
- 3 (3)
- 4 (4)
- 5 (5)
- 6 (6)
- 7 (Very High) (7)

Q272 Gambling Task 8: Imagine choosing between "losing $30 for certain" or a "50% chance of losing $100" (and hence there is a 50% chance of not losing anything). Which option would you choose?  Here you would have to imagine making choices between playing a gamble that can make you lose an amount of money and losing a smaller amount for sure. Each pair of options is again presented as two pie charts. Imagine that a spinner is attached to the center of the pie chart and after the spinner is spun you will lose the money written in the region where and the pointer lands. As you can see, the pie charts representing ‘Sure Loss’ will always make you lose a certain amount while the pie charts representing ‘Loss Gamble’ can make you lose either a bigger amount or zero (the two regions of these pie charts represent the probabilities for each loss respectively). Please circle the pie chart you would prefer (sure loss or gamble) in each pair. Note that there are no correct answers and your choice is a matter of personal preference, but try to choose which option (sure loss or gamble) you would prefer if this choice was made for real. 

- A. Sure Loss (1)
- B. Gamble (2)

End of Block: Gamble With Loss - Task 8

Start of Block: Gamble With Loss - Task 9

Q274 Imagine you are about to play gambling task 9. How would you feel if you choose the "sure loss" of  $192? Rate the valence of your feelings (how positive or negative you feel) on the below scale:

- 1 (Strongly Negative) (1)
- 2 (2)
- 3 (3)
- 4 (Neutral) (4)
- 5 (5)
- 6 (6)
- 7 (Strongly Positive) (7)

Q276 Imagine you are about to play gambling task 9. How would you feel if you choose the "sure loss" of  $192? Rate the intensity of your feelings on the below scale:

- 1 (Very Low) (1)
- 2 (2)
- 3 (3)
- 4 (4)
- 5 (5)
- 6 (6)
- 7 (Very High) (7)

Q278 Imagine you are about to play gambling task 9. How would you feel if you lose $0? Rate the valence of your feelings (how positive or negative you feel) on the below scale:

- 1 (Strongly Negative) (1)
- 2 (2)
- 3 (3)
- 4 (Neutral) (4)
- 5 (5)
- 6 (6)
- 7 (Strongly Positive) (7)

Q280 Imagine you are about to play gambling task 9. How would you feel if you lose $0? Rate the intensity of your feelings on the below scale:

- 1 (Very Low) (1)
- 2 (2)
- 3 (3)
- 4 (4)
- 5 (5)
- 0 (6)
- 7 (Very High) (7)

Q282 Imagine you are about to play gambling task 9. How would you feel if you lose $300? Rate the valence of your feelings (how positive or negative you feel) on the below scale:

- 1 (Strongly Negative) (1)
- 2 (2)
- 3 (3)
- 4 (Neutral) (4)
- 5 (5)
- 6 (6)
- 7 (Strongly Positive) (7)

Q284 Imagine you are about to play gambling task 9. How would you feel if you lose $300? Rate the intensity of your feelings on the below scale:

- 1(Very Low) (1)
- 2 (2)
- 3 (3)
- 4 (4)
- 5 (5)
- 6 (6)
- 7 (Very High) (7)

Q286 Gambling Task 9: Imagine choosing between "losing $30 for certain" or a "50% chance of losing $100" (and hence there is a 50% chance of not losing anything). Which option would you choose?  Here you would have to imagine making choices between playing a gamble that can make you lose an amount of money and losing a smaller amount for sure. Each pair of options is again presented as two pie charts. Imagine that a spinner is attached to the center of the pie chart and after the spinner is spun you will lose the money written in the region where and the pointer lands. As you can see, the pie charts representing ‘Sure Loss’ will always make you lose a certain amount while the pie charts representing ‘Loss Gamble’ can make you lose either a bigger amount or zero (the two regions of these pie charts represent the probabilities for each loss respectively). Please circle the pie chart you would prefer (sure loss or gamble) in each pair. Note that there are no correct answers and your choice is a matter of personal preference, but try to choose which option (sure loss or gamble) you would prefer if this choice was made for real. 

- A. Sure Loss (1)
- B. Gamble (2)

End of Block: Gamble With Loss - Task 9

Start of Block: Gamble With Loss - Task 10

Q302 Imagine you are about to play gambling task 10. How would you feel if you choose the "sure loss" of  $211? Rate the valence of your feelings (how positive or negative you feel) on the below scale:

- 1 (Strongly Negative) (1)
- 2 (2)
- 3 (3)
- 4 (Neutral) (4)
- 5 (5)
- 6 (6)
- 7 (Strongly Positive) (7)

Q304 Imagine you are about to play gambling task 10. How would you feel if you choose the "sure loss" of  $211?  Rate the intensity of your feelings on the below scale:

- 1 (Very Low) (1)
- 2 (2)
- 3 (3)
- 4 (4)
- 5 (5)
- 6 (6)
- 7 (Very High) (7)

Q306 Imagine you are about to play gambling task 10. How would you feel if you lose $0?  Rate the valence of your feelings (how positive or negative you feel) on the below scale:

- 1 (Strongly Negative) (1)
- 2 (2)
- 3 (3)
- 4 (Neutral) (4)
- 5 (5)
- 6 (6)
- 7 (Strongly Positive) (7)

Q308 Imagine you are about to play gambling task 10. How would you feel if you lose $0?  Rate the intensity of your feelings on the below scale:

- 1 (Very Low) (1)
- 2 (2)
- 3 (3)
- 4 (4)
- 5 (5)
- 0 (6)
- 7 (Very High) (7)

Q310 Imagine you are about to play gambling task 10. How would you feel if you lose $400?  Rate the valence of your feelings (how positive or negative you feel) on the below scale:

- 1 (Strongly Negative) (1)
- 2 (2)
- 3 (3)
- 4 (Neutral) (4)
- 5 (5)
- 6 (6)
- 7 (Strongly Positive) (7)

Q312 Imagine you are about to play gambling task 10. How would you feel if you lose $400? Rate the intensity of your feelings on the below scale:

- 1(Very Low) (1)
- 2 (2)
- 3 (3)
- 4 (4)
- 5 (5)
- 6 (6)
- 7 (Very High) (7)

Q314 Gambling Task 10: Imagine choosing between "losing $30 for certain" or a "50% chance of losing $100" (and hence there is a 50% chance of not losing anything). Which option would you choose?  Here you would have to imagine making choices between playing a gamble that can make you lose an amount of money and losing a smaller amount for sure. Each pair of options is again presented as two pie charts. Imagine that a spinner is attached to the center of the pie chart and after the spinner is spun you will lose the money written in the region where and the pointer lands. As you can see, the pie charts representing ‘Sure Loss’ will always make you lose a certain amount while the pie charts representing ‘Loss Gamble’ can make you lose either a bigger amount or zero (the two regions of these pie charts represent the probabilities for each loss respectively). Please circle the pie chart you would prefer (sure loss or gamble) in each pair. Note that there are no correct answers and your choice is a matter of personal preference, but try to choose which option (sure loss or gamble) you would prefer if this choice was made for real. 

- A. Sure Loss (1)
- B. Gamble (2)

End of Block: Gamble With Loss - Task 10

Start of Block: Gamble With Loss - Task 11

Q316 Imagine you are about to play gambling task 11. How would you feel if you choose the "sure loss" of  $98?  Rate the valence of your feelings (how positive or negative you feel) on the below scale:

- 1 (Strongly Negative) (1)
- 2 (2)
- 3 (3)
- 4 (Neutral) (4)
- 5 (5)
- 6 (6)
- 7 (Strongly Positive) (7)

Q318 Imagine you are about to play gambling task 11. How would you feel if you choose the "sure loss" of  $98?  Rate the intensity of your feelings on the below scale:

- 1 (Very Low) (1)
- 2 (2)
- 3 (3)
- 4 (4)
- 5 (5)
- 6 (6)
- 7 (Very High) (7)

Q320 Imagine you are about to play gambling task 11. How would you feel if you lose $0?  Rate the valence of your feelings (how positive or negative you feel) on the below scale:

- 1 (Strongly Negative) (1)
- 2 (2)
- 3 (3)
- 4 (Neutral) (4)
- 5 (5)
- 6 (6)
- 7 (Strongly Positive) (7)

Q322 Imagine you are about to play gambling task 11. How would you feel if you lose $0?  Rate the intensity of your feelings on the below scale:

- 1 (Very Low) (1)
- 2 (2)
- 3 (3)
- 4 (4)
- 5 (5)
- 0 (6)
- 7 (Very High) (7)

Q324 Imagine you are about to play gambling task 11. How would you feel if you lose $400? Rate the valence of your feelings (how positive or negative you feel) on the below scale:

- 1 (Strongly Negative) (1)
- 2 (2)
- 3 (3)
- 4 (Neutral) (4)
- 5 (5)
- 6 (6)
- 7 (Strongly Positive) (7)

Q326 Imagine you are about to play gambling task 11. How would you feel if you lose $400?  Rate the intensity of your feelings on the below scale:

- 1(Very Low) (1)
- 2 (2)
- 3 (3)
- 4 (4)
- 5 (5)
- 6 (6)
- 7 (Very High) (7)

Q328 Gambling Task 11: Imagine choosing between "losing $30 for certain" or a "50% chance of losing $100" (and hence there is a 50% chance of not losing anything). Which option would you choose?  Here you would have to imagine making choices between playing a gamble that can make you lose an amount of money and losing a smaller amount for sure. Each pair of options is again presented as two pie charts. Imagine that a spinner is attached to the center of the pie chart and after the spinner is spun you will lose the money written in the region where and the pointer lands. As you can see, the pie charts representing ‘Sure Loss’ will always make you lose a certain amount while the pie charts representing ‘Loss Gamble’ can make you lose either a bigger amount or zero (the two regions of these pie charts represent the probabilities for each loss respectively). Please circle the pie chart you would prefer (sure loss or gamble) in each pair. Note that there are no correct answers and your choice is a matter of personal preference, but try to choose which option (sure loss or gamble) you would prefer if this choice was made for real. 

- A. Sure Loss (1)
- B. Gamble (2)

End of Block: Gamble With Loss - Task 11

Start of Block: Gamble With Loss - Task 12

Q330 Imagine you are about to play gambling task 12. How would you feel if you choose the "sure loss" of  $211?  Rate the valence of your feelings (how positive or negative you feel) on the below scale:

- 1 (Strongly Negative) (1)
- 2 (2)
- 3 (3)
- 4 (Neutral) (4)
- 5 (5)
- 6 (6)
- 7 (Strongly Positive) (7)

Q332 Imagine you are about to play gambling task 12. How would you feel if you choose the "sure loss" of  $211?  Rate the intensity of your feelings on the below scale:

- 1 (Very Low) (1)
- 2 (2)
- 3 (3)
- 4 (4)
- 5 (5)
- 6 (6)
- 7 (Very High) (7)

Q334 Imagine you are about to play gambling task 12. How would you feel if you lose $0?  Rate the valence of your feelings (how positive or negative you feel) on the below scale:

- 1 (Strongly Negative) (1)
- 2 (2)
- 3 (3)
- 4 (Neutral) (4)
- 5 (5)
- 6 (6)
- 7 (Strongly Positive) (7)

Q336 Imagine you are about to play gambling task 12. How would you feel if you lose $0? Rate the intensity of your feelings on the below scale:

- 1 (Very Low) (1)
- 2 (2)
- 3 (3)
- 4 (4)
- 5 (5)
- 0 (6)
- 7 (Very High) (7)

Q338 Imagine you are about to play gambling task 12. How would you feel if you lose $400? Rate the valence of your feelings (how positive or negative you feel) on the below scale:

- 1 (Strongly Negative) (1)
- 2 (2)
- 3 (3)
- 4 (Neutral) (4)
- 5 (5)
- 6 (6)
- 7 (Strongly Positive) (7)

Q340 Imagine you are about to play gambling task 12. How would you feel if you lose $400? Rate the intensity of your feelings on the below scale:

- 1(Very Low) (1)
- 2 (2)
- 3 (3)
- 4 (4)
- 5 (5)
- 6 (6)
- 7 (Very High) (7)

Q342 Gambling Task 12: Imagine choosing between "losing $30 for certain" or a "50% chance of losing $100" (and hence there is a 50% chance of not losing anything). Which option would you choose?  Here you would have to imagine making choices between playing a gamble that can make you lose an amount of money and losing a smaller amount for sure. Each pair of options is again presented as two pie charts. Imagine that a spinner is attached to the center of the pie chart and after the spinner is spun you will lose the money written in the region where and the pointer lands. As you can see, the pie charts representing ‘Sure Loss’ will always make you lose a certain amount while the pie charts representing ‘Loss Gamble’ can make you lose either a bigger amount or zero (the two regions of these pie charts represent the probabilities for each loss respectively). Please circle the pie chart you would prefer (sure loss or gamble) in each pair. Note that there are no correct answers and your choice is a matter of personal preference, but try to choose which option (sure loss or gamble) you would prefer if this choice was made for real.

- A. Sure Loss (1)
- B. Gamble (2)

*(4) Anticipatory positive*

SCENARIO: GAMBLE WITH GAINImagine choosing between "receiving $30 for certain" or a "50% chance of winning $100". Which option would you choose?  Here you would have to imagine making choices between playing a gamble to receive an amount of money and taking a smaller amount for sure. Each pair of options is presented as two pie charts. Imagine that a spinner is attached to the center of the pie chart and after the spinner is spun you will receive the money written in the region where and the pointer lands. As you can see, the pie charts representing ‘Sure Gain’ will always give you a certain amount while the pie charts representing ‘Gamble’ offer either a bigger amount or zero (the two regions of these pie charts represent the probabilities for each amount respectively).

Q18 Imagine you are about to play gambling task 1. How would you feel if you choose the "sure gain" of  $16? Rate the valence of your feelings (how positive or negative you feel) on the below scale: 

- 1 (Strongly Negative) (1)
- 2 (2)
- 3 (3)
- 4 (Neutral) (4)
- 5 (5)
- 6 (6)
- 7 (Strongly Positive) (7)

Q20 Imagine you are about to play gambling task 1. How would you feel if you choose the "sure gain" of  $16? Rate the intensity of your feelings on the below scale:

- 1 (Very Low) (1)
- 2 (2)
- 3 (3)
- 4 (4)
- 5 (5)
- 6 (6)
- 7 (Very High) (7)

Q22 Imagine you are about to play gambling task 1. How would you feel if you win $0? Rate the valence of your feelings (how positive or negative you feel) on the below scale:

- 1 (Strongly Negative) (1)
- 2 (2)
- 3 (3)
- 4 (Neutral) (4)
- 5 (5)
- 6 (6)
- 7 (Strongly Positive) (7)

Q24 Imagine you are about to play gambling task 1. How would you feel if you win $0? Rate the intensity of your feelings on the below scale

- 1 (Very Low) (1)
- 2 (2)
- 3 (3)
- 4 (4)
- 5 (5)
- 6 (6)
- 7 (Very High) (7)

Q26 Imagine you are about to play gambling task 1. How would you feel if you win $100? Rate the valence of your feelings (how positive or negative you feel) on the below scale:

- 1 (Strongly Negative) (1)
- 2 (2)
- 3 (3)
- 4 (Neutral) (4)
- 5 (5)
- 6 (6)
- 7 (Strongly Positive) (7)

Q28 Imagine you are about to play gambling task 1. How would you feel if you win $100?  Rate the intensity of your feelings on the below scale:

- 1 (Low) (1)
- 2 (2)
- 3 (3)
- 4 (4)
- 5 (5)
- 6 (6)
- 7 (High) (7)

Q30 Gambling Task 1:Imagine choosing between "receiving $30 for certain" or a "50% chance of winning $100". Which option would you choose?  Here you would have to imagine making choices between playing a gamble to receive an amount of money and taking a smaller amount for sure. Each pair of options is presented as two pie charts. Imagine that a spinner is attached to the center of the pie chart and after the spinner is spun you will receive the money written in the region where and the pointer lands. As you can see, the pie charts representing ‘Sure Gain’ will always give you a certain amount while the pie charts representing ‘Gamble’ offer either a bigger amount or zero (the two regions of these pie charts represent the probabilities for each amount respectively). Please circle the pie chart you would prefer (the sure amount or the gamble) in each pair. Note that there are no correct answers and your choice is a matter of personal preference, but try to choose which option (sure amount or a gamble) you would prefer if this choice was made for real.

- A. Sure Gain (1)
- B. Gamble (2)

End of Block: Gamble With Gain - Task 1

Start of Block: Gamble With Gain - Task 2

Q33 Imagine you are about to play gambling task 2. How would you feel if you choose the "sure gain" of $46? Rate the valence of your feelings (how positive or negative you feel) on the below scale: 

- 1 (Strongly Negative) (1)
- 2 (2)
- 3 (3)
- 4 (Neutral) (4)
- 5 (5)
- 6 (6)
- 7 (Strongly Positive) (7)

Q35 Imagine you are about to play gambling task 2. How would you feel if you choose the "sure gain" of $46? Rate the valence of your feelings (how positive or negative you feel) on the below scale:

- 1 (Very Low) (1)
- 2 (2)
- 3 (3)
- 4 (4)
- 5 (5)
- 6 (6)
- 7 (Very High) (7)

Q37 Imagine you are about to play gambling task 2. How would you feel if you win $0? Rate the valence of your feelings (how positive or negative you feel) on the below scale:

- 1 (Strongly Negative) (1)
- 2 (2)
- 3 (3)
- 4 (Neutral) (4)
- 5 (5)
- 6 (6)
- 7 (Strongly Positive) (7)

Q39 Imagine you are about to play gambling task 2. How would you feel if you win $0? Rate the intensity of your feelings on the below scale:

- 1 (Very Low) (1)
- 2 (2)
- 3 (3)
- 4 (4)
- 5 (5)
- 6 (6)
- 7 (Very High) (7)

Q41 Imagine you are about to play gambling task 2. How would you feel if you win $100? Rate the valence of your feelings (how positive or negative you feel) on the below scale:

- 1 (Strongly Negative) (1)
- 2 (2)
- 3 (3)
- 4 (Neutral) (4)
- 5 (5)
- 6 (6)
- 7 (Strongly Positive) (7)

Q43 Imagine you are about to play gambling task 2. How would you feel if you win $100? Rate the intensity of your feelings on the below scale:

- 1 (Low) (1)
- 2 (2)
- 3 (3)
- 4 (4)
- 5 (5)
- 6 (6)
- 7 (High) (7)

Q45 Gambling Task 2:
Imagine choosing between "receiving $30 for certain" or a "50% chance of winning $100". Which option would you choose?  Here you would have to imagine making choices between playing a gamble to receive an amount of money and taking a smaller amount for sure. Each pair of options is presented as two pie charts. Imagine that a spinner is attached to the center of the pie chart and after the spinner is spun you will receive the money written in the region where and the pointer lands. As you can see, the pie charts representing ‘Sure Gain’ will always give you a certain amount while the pie charts representing ‘Gamble’ offer either a bigger amount or zero (the two regions of these pie charts represent the probabilities for each amount respectively). Please circle the pie chart you would prefer (the sure amount or the gamble) in each pair. Note that there are no correct answers and your choice is a matter of personal preference, but try to choose which option (sure amount or a gamble) you would prefer if this choice was made for real.

- A. Sure Gain (1)
- B. Gamble (2)

End of Block: Gamble With Gain - Task 2

Start of Block: Gamble With Gain - Task 3

Q48 Imagine you are about to play gambling task 3. How would you feel if you choose the "sure gain" of  $76? Rate the valence of your feelings (how positive or negative you feel) on the below scale:

- 1 (Strongly Negative) (1)
- 2 (2)
- 3 (3)
- 4 (Neutral) (4)
- 5 (5)
- 6 (6)
- 7 (Strongly Positive) (7)

Q50 Imagine you are about to play gambling task 3. How would you feel if you choose the "sure gain" of  $76? Rate the intensity of your feelings on the below scale:

- 1 (Very Low) (1)
- 2 (2)
- 3 (3)
- 4 (4)
- 5 (5)
- 6 (6)
- 7 (Very High) (7)

Q52 Imagine you are about to play gambling task 3. How would you feel if you win $0? Rate the valence of your feelings (how positive or negative you feel) on the below scale:

- 1 (Strongly Negative) (1)
- 2 (2)
- 3 (3)
- 4 (Neutral) (4)
- 5 (5)
- 6 (6)
- 7 (Strongly Positive) (7)

Q54 Imagine you are about to play gambling task 3. How would you feel if you win $0? Rate the intensity of your feelings on the below scale:

- 1 (Very Low) (1)
- 2 (2)
- 3 (3)
- 4 (4)
- 5 (5)
- 6 (6)
- 7 (Very High) (7)

Q56 Imagine you are about to play gambling task 3. How would you feel if you win $100? Rate the valence of your feelings (how positive or negative you feel) on the below scale:

- 1 (Strongly Negative) (1)
- 2 (2)
- 3 (3)
- 4 (Neutral) (4)
- 5 (5)
- 6 (6)
- 7 (Strongly Positive) (7)

Q58 Imagine you are about to play gambling task 3. How would you feel if you win $100? Rate the intensity of your feelings on the below scale:

- 1 (Low) (1)
- 2 (2)
- 3 (3)
- 4 (4)
- 5 (5)
- 6 (6)
- 7 (High) (7)

Q60 Gambling Task 3: Imagine choosing between "receiving $30 for certain" or a "50% chance of winning $100". Which option would you choose?  Here you would have to imagine making choices between playing a gamble to receive an amount of money and taking a smaller amount for sure. Each pair of options is presented as two pie charts. Imagine that a spinner is attached to the center of the pie chart and after the spinner is spun you will receive the money written in the region where and the pointer lands. As you can see, the pie charts representing ‘Sure Gain’ will always give you a certain amount while the pie charts representing ‘Gamble’ offer either a bigger amount or zero (the two regions of these pie charts represent the probabilities for each amount respectively). Please circle the pie chart you would prefer (the sure amount or the gamble) in each pair. Note that there are no correct answers and your choice is a matter of personal preference, but try to choose which option (sure amount or a gamble) you would prefer if this choice was made for real.

- A. Sure Gain (1)
- B. Gamble (2)

End of Block: Gamble With Gain - Task 3

Start of Block: Gamble With Gain - Task 4

Q76 Imagine you are about to play gambling task 4. How would you feel if you choose the "sure gain" of  $15? Rate the valence of your feelings (how positive or negative you feel) on the below scale: 

- 1 (Strongly Negative) (1)
- 2 (2)
- 3 (3)
- 4 (Neutral) (4)
- 5 (5)
- 6 (6)
- 7 (Strongly Positive) (7)

Q78 Imagine you are about to play gambling task 4. How would you feel if you choose the "sure gain" of  $15? Rate the intensity of your feelings on the below scale:

- 1 (Very Low) (1)
- 2 (2)
- 3 (3)
- 4 (4)
- 5 (5)
- 6 (6)
- 7 (Very High) (7)

Q80 Imagine you are about to play gambling task 4. How would you feel if you win $0?  Rate the valence of your feelings (how positive or negative you feel) on the below scale:

- 1 (Strongly Negative) (1)
- 2 (2)
- 3 (3)
- 4 (Neutral) (4)
- 5 (5)
- 6 (6)
- 7 (Strongly Positive) (7)

Q82 Imagine you are about to play gambling task 4. How would you feel if you win $0?  Rate the intensity of your feelings on the below scale:

- 1 (Very Low) (1)
- 2 (2)
- 3 (3)
- 4 (4)
- 5 (5)
- 6 (6)
- 7 (Very High) (7)

Q84 Imagine you are about to play gambling task 4. How would you feel if you win $200? Rate the valence of your feelings (how positive or negative you feel) on the below scale:

- 1 (Strongly Negative) (1)
- 2 (2)
- 3 (3)
- 4 (Neutral) (4)
- 5 (5)
- 6 (6)
- 7(Strongly Positive) (7)

Q86 Imagine you are about to play gambling task 4. How would you feel if you win $200? Rate the intensity of your feelings on the below scale:

- 1 (Low) (1)
- 2 (2)
- 3 (3)
- 4 (4)
- 5 (5)
- 6 (6)
- 7 (High) (7)

Q88 Gambling Task 4: Imagine choosing between "receiving $30 for certain" or a "50% chance of winning $100". Which option would you choose?  Here you would have to imagine making choices between playing a gamble to receive an amount of money and taking a smaller amount for sure. Each pair of options is presented as two pie charts. Imagine that a spinner is attached to the center of the pie chart and after the spinner is spun you will receive the money written in the region where and the pointer lands. As you can see, the pie charts representing ‘Sure Gain’ will always give you a certain amount while the pie charts representing ‘Gamble’ offer either a bigger amount or zero (the two regions of these pie charts represent the probabilities for each amount respectively). Please circle the pie chart you would prefer (the sure amount or the gamble) in each pair. Note that there are no correct answers and your choice is a matter of personal preference, but try to choose which option (sure amount or a gamble) you would prefer if this choice was made for real.

- A. Sure Gain (1)
- B. Gamble (2)

End of Block: Gamble With Gain - Task 4

Start of Block: Gamble With Gain - Task 5

Q90 Imagine you are about to play gambling task 5. How would you feel if you choose the "sure gain" of  $72? Rate the valence of your feelings (how positive or negative you feel) on the below scale: 

- 1 (Strongly Negative) (1)
- 2 (2)
- 3 (3)
- 4 (Neutral) (4)
- 5 (5)
- 6 (6)
- 7 (Strongly Positive) (7)

Q92 Imagine you are about to play gambling task 5. How would you feel if you choose the "sure gain" of  $72? Rate the intensity of your feelings on the below scale:

- 1 (Very Low) (1)
- 2 (2)
- 3 (3)
- 4 (4)
- 5 (5)
- 6 (6)
- 7 (Very High) (7)

Q94 Imagine you are about to play gambling task 5. How would you feel if you win $0? Rate the valence of your feelings (how positive or negative you feel) on the below scale:

- 1 (Strongly Negative) (1)
- 2 (2)
- 3 (3)
- 4 (Neutral) (4)
- 5 (5)
- 6 (6)
- 7 (Strongly Positive) (7)

Q96 Imagine you are about to play gambling task 5. How would you feel if you win $0? Rate the intensity of your feelings on the below scale:

- 1 (Very Low) (1)
- 2 (2)
- 3 (3)
- 4 (4)
- 5 (5)
- 6 (6)
- 7 (Very High) (7)

Q98 Imagine you are about to play gambling task 5. How would you feel if you win $200? Rate the valence of your feelings (how positive or negative you feel) on the below scale:

- 1 (Strongly Negative) (1)
- 2 (2)
- 3 (3)
- 4 (Neutral) (4)
- 5 (5)
- 6 (6)
- 7 (Strongly Positive) (7)

Q100 Imagine you are about to play gambling task 5. How would you feel if you win $200?  Rate the intensity of your feelings on the below scale:

- 1 (Low) (1)
- 2 (2)
- 3 (3)
- 4 (4)
- 5 (5)
- 6 (6)
- 7 (High) (7)

Q102 Gambling Task 5: Imagine choosing between "receiving $30 for certain" or a "50% chance of winning $100". Which option would you choose?  Here you would have to imagine making choices between playing a gamble to receive an amount of money and taking a smaller amount for sure. Each pair of options is presented as two pie charts. Imagine that a spinner is attached to the center of the pie chart and after the spinner is spun you will receive the money written in the region where and the pointer lands. As you can see, the pie charts representing ‘Sure Gain’ will always give you a certain amount while the pie charts representing ‘Gamble’ offer either a bigger amount or zero (the two regions of these pie charts represent the probabilities for each amount respectively). Please circle the pie chart you would prefer (the sure amount or the gamble) in each pair. Note that there are no correct answers and your choice is a matter of personal preference, but try to choose which option (sure amount or a gamble) you would prefer if this choice was made for real.

- A. Sure Gain (1)
- B. Gamble (2)

End of Block: Gamble With Gain - Task 5

Start of Block: Gamble with Gain - Task 6

Q105 Imagine you are about to play gambling task 6. How would you feel if you choose the "sure gain" of  $142? Rate the valence of your feelings (how positive or negative you feel) on the below scale:

- 1 (Strongly Negative) (1)
- 2 (2)
- 3 (3)
- 4 (Neutral) (4)
- 5 (5)
- 6 (6)
- 7 (Strongly Positive) (7)

Q107 Imagine you are about to play gambling task 6. How would you feel if you choose the "sure gain" of  $142? Rate the intensity of your feelings on the below scale:

- 1 (Very Low) (1)
- 2 (2)
- 3 (3)
- 4 (4)
- 5 (5)
- 6 (6)
- 7 (Very High) (7)

Q109 Imagine you are about to play gambling task 6. How would you feel if you win $0? Rate the valence of your feelings (how positive or negative you feel) on the below scale:

- 1 Strongly Negative) (1)
- 2 (2)
- 3 (3)
- 4 (Neutral) (4)
- 5 (5)
- 6 (6)
- 7 (Strongly Positive) (7)

Q111 Imagine you are about to play gambling task 6. How would you feel if you win $0? Rate the intensity of your feelings on the below scale:

- 1 (Very Low) (1)
- 2 (2)
- 3 (3)
- 4 (4)
- 5 (5)
- 6 (6)
- 7 (Very High) (7)

Q113 Imagine you are about to play gambling task 6. How would you feel if you win $200? Rate the valence of your feelings (how positive or negative you feel) on the below scale:

- 1 (Strongly Negative) (1)
- 2 (2)
- 3 (3)
- 4 (Neutral) (4)
- 5 (5)
- 6 (6)
- 7 (Strongly Positive) (7)

Q115 Imagine you are about to play gambling task 6. How would you feel if you win $200? Rate the intensity of your feelings on the below scale:

- 1 (Low) (1)
- 2 (2)
- 3 (3)
- 4 (4)
- 5 (5)
- 6 (6)
- 7 (High) (7)

Q117 Gambling Task 6: Imagine choosing between "receiving $30 for certain" or a "50% chance of winning $100". Which option would you choose?  Here you would have to imagine making choices between playing a gamble to receive an amount of money and taking a smaller amount for sure. Each pair of options is presented as two pie charts. Imagine that a spinner is attached to the center of the pie chart and after the spinner is spun you will receive the money written in the region where and the pointer lands. As you can see, the pie charts representing ‘Gain’ will always give you a certain amount while the pie charts representing ‘Gamble’ offer either a bigger amount or zero (the two regions of these pie charts represent the probabilities for each amount respectively). Please circle the pie chart you would prefer (the sure amount or the gamble) in each pair. Note that there are no correct answers and your choice is a matter of personal preference, but try to choose which option (sure amount or a gamble) you would prefer if this choice was made for real.

- A. Sure Gain (1)
- B. Gamble (2)

End of Block: Gamble with Gain - Task 6

Start of Block: Gamble With Gain - Task 7

Q133 Imagine you are about to play gambling task 7. How would you feel if you choose the "sure gain" of  $95? Rate the valence of your feelings (how positive or negative you feel) on the below scale: 

- 1 (Strongly Negative) (1)
- 2 (2)
- 3 (3)
- 4 (Neutral) (4)
- 5 (5)
- 6 (6)
- 7 (Strongly Positive) (7)

Q135 Imagine you are about to play gambling task 7. How would you feel if you choose the "sure gain" of  $95?  Rate the intensity of your feelings on the below scale:

- 1 (Very Low) (1)
- 2 (2)
- 3 (3)
- 4 (4)
- 5 (5)
- 6 (6)
- 7 (Very High) (7)

Q137 Imagine you are about to play gambling task 7. How would you feel if you win $0? Rate the valence of your feelings (how positive or negative you feel) on the below scale:

- 1 (Strongly Negative) (1)
- 2 (2)
- 3 (3)
- 4 (Neutral) (4)
- 5 (5)
- 6 (6)
- 7 (Strongly Positive) (7)

Q139 Imagine you are about to play gambling task 7. How would you feel if you win $0? Rate the intensity of your feelings on the below scale:

- 1 (Very Low) (1)
- 2 (2)
- 3 (3)
- 4 (4)
- 5 (5)
- 6 (6)
- 7 (Very High) (7)

Q141 Imagine you are about to play gambling task 7. How would you feel if you win $300? Rate the valence of your feelings (how positive or negative you feel) on the below scale:

- 1 (Strongly Negative) (1)
- 2 (2)
- 3 (3)
- 4 (Neutral) (4)
- 5 (5)
- 6 (6)
- 7 (Strongly Positive) (7)

Q143 Imagine you are about to play gambling task 7. How would you feel if you win $300? Rate the intensity of your feelings on the below scale:

- 1 (Low) (1)
- 2 (2)
- 3 (3)
- 4 (4)
- 5 (5)
- 6 (6)
- 7 (High) (7)

Q145 Gambling Task 7: Imagine choosing between "receiving $30 for certain" or a "50% chance of winning $100". Which option would you choose?  Here you would have to imagine making choices between playing a gamble to receive an amount of money and taking a smaller amount for sure. Each pair of options is presented as two pie charts. Imagine that a spinner is attached to the center of the pie chart and after the spinner is spun you will receive the money written in the region where and the pointer lands. As you can see, the pie charts representing ‘Sure Gain’ will always give you a certain amount while the pie charts representing ‘Gamble’ offer either a bigger amount or zero (the two regions of these pie charts represent the probabilities for each amount respectively). Please circle the pie chart you would prefer (the sure amount or the gamble) in each pair. Note that there are no correct answers and your choice is a matter of personal preference, but try to choose which option (sure amount or a gamble) you would prefer if this choice was made for real.

- A. Sure Gain (1)
- B. Gamble (2)

End of Block: Gamble With Gain - Task 7

Start of Block: Gamble With Gain - Task 8

Q147 Imagine you are about to play gambling task 8. How would you feel if you choose the "sure gain" of  $70?  Rate the valence of your feelings (how positive or negative you feel) on the below scale: 

- 1 (Strongly Negative) (1)
- 2 (2)
- 3 (3)
- 4 (Neutral) (4)
- 5 (5)
- 6 (6)
- 7 (Strongly Positive) (7)

Q149 Imagine you are about to play gambling task 8. How would you feel if you choose the "sure gain" of  $70? Rate the intensity of your feelings on the below scale:

- 1 (Very Low) (1)
- 2 (2)
- 3 (3)
- 4 (4)
- 5 (5)
- 6 (6)
- 7 (Very High) (7)

Q151 Imagine you are about to play gambling task 8. How would you feel if you win $0?  Rate the valence of your feelings (how positive or negative you feel) on the below scale:

- 1 (Strongly Negative) (1)
- 2 (2)
- 3 (3)
- 4 (Neutral) (4)
- 5 (5)
- 6 (6)
- 7 (Strongly Positive) (7)

Q153 Imagine you are about to play gambling task 8. How would you feel if you win $0? Rate the intensity of your feelings on the below scale:

- 1 (Very Low) (1)
- 2 (2)
- 3 (3)
- 4 (4)
- 5 (5)
- 6 (6)
- 7 (Very High) (7)

Q155 Imagine you are about to play gambling task 8. How would you feel if you win $300? Rate the valence of your feelings (how positive or negative you feel) on the below scale:

- 1 (Strongly Negative) (1)
- 2 (2)
- 3 (3)
- 4 (Neutral) (4)
- 5 (5)
- 6 (6)
- 7 (Strongly Positive) (7)

Q157 Imagine you are about to play gambling task 8. How would you feel if you win $300? Rate the intensity of your feelings on the below scale:

- 1 (Low) (1)
- 2 (2)
- 3 (3)
- 4 (4)
- 5 (5)
- 6 (6)
- 7 (High) (7)

Q159 Gambling Task 8: Imagine choosing between "receiving $30 for certain" or a "50% chance of winning $100". Which option would you choose?  Here you would have to imagine making choices between playing a gamble to receive an amount of money and taking a smaller amount for sure. Each pair of options is presented as two pie charts. Imagine that a spinner is attached to the center of the pie chart and after the spinner is spun you will receive the money written in the region where and the pointer lands. As you can see, the pie charts representing ‘Sure Gain’ will always give you a certain amount while the pie charts representing ‘Gamble’ offer either a bigger amount or zero (the two regions of these pie charts represent the probabilities for each amount respectively). Please circle the pie chart you would prefer (the sure amount or the gamble) in each pair. Note that there are no correct answers and your choice is a matter of personal preference, but try to choose which option (sure amount or a gamble) you would prefer if this choice was made for real.

- A. Sure Gain (1)
- B. Gamble (2)

End of Block: Gamble With Gain - Task 8

Start of Block: Gamble With Gain - Task 9

Q162 Imagine you are about to play gambling task 9. How would you feel if you choose the "sure gain" of  $192? Rate the valence of your feelings (how positive or negative you feel) on the below scale: 

- 1 (Strongly Negative) (1)
- 2 (2)
- 3 (3)
- 4 (Neutral) (4)
- 5 (5)
- 6 (6)
- 7 (Strongly Positive) (7)

Q164 Imagine you are about to play gambling task 9. How would you feel if you choose the "sure gain" of  $192? Rate the intensity of your feelings on the below scale:

- 1 (Very Low) (1)
- 2 (2)
- 3 (3)
- 4 (4)
- 5 (5)
- 6 (6)
- 7 (Very High) (7)

Q166 Imagine you are about to play gambling task 9. How would you feel if you win $0?  Rate the valence of your feelings (how positive or negative you feel) on the below scale:

- 1 (Strongly Negative) (1)
- 2 (2)
- 3 (3)
- 4 (Neutral) (4)
- 5 (5)
- 6 (6)
- 7 (Strongly Positive) (7)

Q168 Imagine you are about to play gambling task 9. How would you feel if you win $0? Rate the intensity of your feelings on the below scale:

- 1 (Very Low) (1)
- 2 (2)
- 3 (3)
- 4 (4)
- 5 (5)
- 6 (6)
- 7 (Very High) (7)

Q170 Imagine you are about to play gambling task 9. How would you feel if you win $300? Rate the valence of your feelings (how positive or negative you feel) on the below scale:

- 1 (Strongly Negative) (1)
- 2 (2)
- 3 (3)
- 4 (Neutral) (4)
- 5 (5)
- 6 (6)
- 7 (Strongly Positive) (7)

Q172 Imagine you are about to play gambling task 9. How would you feel if you win $300?  Rate the intensity of your feelings on the below scale:

- 1 (Low) (1)
- 2 (2)
- 3 (3)
- 4 (4)
- 5 (5)
- 6 (6)
- 7 (High) (7)

Q174 Gambling Task 9: Imagine choosing between "receiving $30 for certain" or a "50% chance of winning $100". Which option would you choose?  Here you would have to imagine making choices between playing a gamble to receive an amount of money and taking a smaller amount for sure. Each pair of options is presented as two pie charts. Imagine that a spinner is attached to the center of the pie chart and after the spinner is spun you will receive the money written in the region where and the pointer lands. As you can see, the pie charts representing ‘Sure Gain’ will always give you a certain amount while the pie charts representing ‘Gamble’ offer either a bigger amount or zero (the two regions of these pie charts represent the probabilities for each amount respectively). Please circle the pie chart you would prefer (the sure amount or the gamble) in each pair. Note that there are no correct answers and your choice is a matter of personal preference, but try to choose which option (sure amount or a gamble) you would prefer if this choice was made for real.

- A. Sure Gain (1)
- B. Gamble (2)

End of Block: Gamble With Gain - Task 9

Start of Block: Gamble With Gain - Task 10

Q190 Imagine you are about to play gambling task 10. How would you feel if you choose the "sure gain" of  $98? Rate the valence of your feelings (how positive or negative you feel) on the below scale: 

- 1 (Strongly Negative) (1)
- 2 (2)
- 3 (3)
- 4 (Neutral) (4)
- 5 (5)
- 6 (6)
- 7 (Strongly Positive) (7)

Q192 Imagine you are about to play gambling task 10. How would you feel if you choose the "sure gain" of  $98? Rate the intensity of your feelings on the below scale:

- 1 (Very Low) (1)
- 2 (2)
- 3 (3)
- 4 (4)
- 5 (5)
- 6 (6)
- 7 (Very High) (7)

Q194 Imagine you have already played gambling task 10 and won $0. Rate the valence of your feelings (how positive or negative you feel) on the below scale:

- 1 (Strongly Negative) (1)
- 2 (2)
- 3 (3)
- 4 (Neutral) (4)
- 5 (5)
- 6 (6)
- 7 (Strongly Positive) (7)

Q196 Imagine you have already played gambling task 10 and won $0. Rate the intensity of your feelings on the below scale:

- 1 (Very Low) (1)
- 2 (2)
- 3 (3)
- 4 (4)
- 5 (5)
- 6 (6)
- 7 (Very High) (7)

Q198 Imagine you have already played gambling task 10 and won $400. Rate the valence of your feelings (how positive or negative you feel) on the below scale:

- 1 (Strongly Negative) (1)
- 2 (2)
- 3 (3)
- 4 (Neutral) (4)
- 5 (5)
- 6 (6)
- 7 (Strongly Positive) (7)

Q200 Imagine you have already played gambling task 10 and won $400. Rate the intensity of your feelings on the below scale:

- 1 (Low) (1)
- 2 (2)
- 3 (3)
- 4 (4)
- 5 (5)
- 6 (6)
- 7 (High) (7)

Q202 Gambling Task 10: Imagine choosing between "receiving $30 for certain" or a "50% chance of winning $100". Which option would you choose?  Here you would have to imagine making choices between playing a gamble to receive an amount of money and taking a smaller amount for sure. Each pair of options is presented as two pie charts. Imagine that a spinner is attached to the center of the pie chart and after the spinner is spun you will receive the money written in the region where and the pointer lands. As you can see, the pie charts representing ‘Sure Gain’ will always give you a certain amount while the pie charts representing ‘Gamble’ offer either a bigger amount or zero (the two regions of these pie charts represent the probabilities for each amount respectively). Please circle the pie chart you would prefer (the sure amount or the gamble) in each pair. Note that there are no correct answers and your choice is a matter of personal preference, but try to choose which option (sure amount or a gamble) you would prefer if this choice was made for real.

- A. Sure Gain (1)
- B. Gamble (2)

End of Block: Gamble With Gain - Task 10

Start of Block: Gamble With Gain - Task 11

Q204 Imagine you are about to play gambling task 11. How would you feel if you choose the "sure gain" of  $211? Rate the valence of your feelings (how positive or negative you feel) on the below scale: 

- 1 (Strongly Negative) (1)
- 2 (2)
- 3 (3)
- 4 (Neutral) (4)
- 5 (5)
- 6 (6)
- 7 (Strongly Positive) (7)

Q206 Imagine you are about to play gambling task 11. How would you feel if you choose the "sure gain" of  $211? Rate the intensity of your feelings on the below scale:

- 1 (Very Low) (1)
- 2 (2)
- 3 (3)
- 4 (4)
- 5 (5)
- 6 (6)
- 7 (Very High) (7)

Q208 Imagine you are about to play gambling task 11. How would you feel if you win $0? Rate the valence of your feelings (how positive or negative you feel) on the below scale:

- 1 (Strongly Negative) (1)
- 2 (2)
- 3 (3)
- 4 (Neutral) (4)
- 5 (5)
- 6 (6)
- 7 (Strongly Positive) (7)

Q210 Imagine you are about to play gambling task 11. How would you feel if you win $0? Rate the intensity of your feelings on the below scale:

- 1 (Very Low) (1)
- 2 (2)
- 3 (3)
- 4 (4)
- 5 (5)
- 6 (6)
- 7 (Very High) (7)

Q212 Imagine you are about to play gambling task 11. How would you feel if you win $400? Rate the valence of your feelings (how positive or negative you feel) on the below scale:

- 1 (Strongly Negative) (1)
- 2 (2)
- 3 (3)
- 4 (Neutral) (4)
- 5 (5)
- 6 (6)
- 7 (Strongly Positive) (7)

Q214 Imagine you are about to play gambling task 11. How would you feel if you win $400? Rate the intensity of your feelings on the below scale:

- 1 (Low) (1)
- 2 (2)
- 3 (3)
- 4 (4)
- 5 (5)
- 6 (6)
- 7 (High) (7)

Q216 Gambling Task 11: Imagine choosing between "receiving $30 for certain" or a "50% chance of winning $100". Which option would you choose?  Here you would have to imagine making choices between playing a gamble to receive an amount of money and taking a smaller amount for sure. Each pair of options is presented as two pie charts. Imagine that a spinner is attached to the center of the pie chart and after the spinner is spun you will receive the money written in the region where and the pointer lands. As you can see, the pie charts representing ‘Sure Gain’ will always give you a certain amount while the pie charts representing ‘Gamble’ offer either a bigger amount or zero (the two regions of these pie charts represent the probabilities for each amount respectively). Please circle the pie chart you would prefer (the sure amount or the gamble) in each pair. Note that there are no correct answers and your choice is a matter of personal preference, but try to choose which option (sure amount or a gamble) you would prefer if this choice was made for real.

- A. Sure Gain (1)
- B. Gamble (2)

End of Block: Gamble With Gain - Task 11

Start of Block: Gamble With Gain - Task 12

Q218 Imagine you are about to play gambling task 12. How would you feel if you choose the "sure gain" of  $211? Rate the valence of your feelings (how positive or negative you feel) on the below scale: 

- 1 (Strongly Negative) (1)
- 2 (2)
- 3 (3)
- 4 (Neutral) (4)
- 5 (5)
- 6 (6)
- 7 (Strongly Positive) (7)

Q220 Imagine you are about to play gambling task 12. How would you feel if you choose the "sure gain" of  $211? Rate the intensity of your feelings on the below scale:

- 1 (Very Low) (1)
- 2 (2)
- 3 (3)
- 4 (4)
- 5 (5)
- 6 (6)
- 7 (Very High) (7)

Q222 Imagine you are about to play gambling task 12. How would you feel if you win $0? Rate the valence of your feelings (how positive or negative you feel) on the below scale:

- 1 (Strongly Negative) (1)
- 2 (2)
- 3 (3)
- 4 (Neutral) (4)
- 5 (5)
- 6 (6)
- 7 (Strongly Positive) (7)

Q224 Imagine you are about to play gambling task 12. How would you feel if you win $0? Rate the intensity of your feelings on the below scale:

- 1 (Very Low) (1)
- 2 (2)
- 3 (3)
- 4 (4)
- 5 (5)
- 6 (6)
- 7 (Very High) (7)

Q226 Imagine you are about to play gambling task 12. How would you feel if you win $400? Rate the valence of your feelings (how positive or negative you feel) on the below scale:

- 1 (Strongly Negative) (1)
- 2 (2)
- 3 (3)
- 4 (Neutral) (4)
- 5 (5)
- 6 (6)
- 7 (Strongly Positive) (7)

Q228 Imagine you are about to play gambling task 12. How would you feel if you win $400? Rate the intensity of your feelings on the below scale:

- 1 (Low) (1)
- 2 (2)
- 3 (3)
- 4 (4)
- 5 (5)
- 6 (6)
- 7 (High) (7)

Q230 Gambling Task 12: Imagine choosing between "receiving $30 for certain" or a "50% chance of winning $100". Which option would you choose?  Here you would have to imagine making choices between playing a gamble to receive an amount of money and taking a smaller amount for sure. Each pair of options is presented as two pie charts. Imagine that a spinner is attached to the center of the pie chart and after the spinner is spun you will receive the money written in the region where and the pointer lands. As you can see, the pie charts representing ‘Sure Gain’ will always give you a certain amount while the pie charts representing ‘Gamble’ offer either a bigger amount or zero (the two regions of these pie charts represent the probabilities for each amount respectively). Please circle the pie chart you would prefer (the sure amount or the gamble) in each pair. Note that there are no correct answers and your choice is a matter of personal preference, but try to choose which option (sure amount or a gamble) you would prefer if this choice was made for real.

- A. Sure Gain (1)
- B. Gamble (2)
